# Non-canonical NOTCH1 signaling regulates ferroptosis vulnerability in dormant lung cancer cells with stable resistance

**DOI:** 10.1038/s41419-025-08355-9

**Published:** 2025-12-26

**Authors:** Hongli Huang, Yihan Chai, Xuewei Wu, Sen Wang, Binglin Li, Yanyan Wang, Wen Zheng, Yuefeng Wu, Di Meng, Hua Wang, Zhengliang Tu, Chengli Du, Xiayi Lyu, Guiying Li, Wei Guo

**Affiliations:** 1 Key Laboratory for Molecular Enzymology and Engineering of the Ministry of Education, School of Life Sciences, Jilin University, Changchun, China; 2 Zhejiang University-University of Edinburgh Institute, School of Medicine, Zhejiang University, Jiaxing, China; 3 Edinburgh Medical School: Biomedical Sciences, College of Medicine and Veterinary Medicine, The University of Edinburgh, Edinburgh, UK; 4 Jiangzhong Cancer Research Center, Jiangxi University of Chinese Medicine, Nanchang, China; 5 School of Integrated Traditional Chinese and Western Medicine, Nanchang Medical College, Nanchang, China; 6 Westlake Laboratory of Life Sciences and Biomedicine, School of Life Sciences, Westlake University, Hangzhou, China; 7 Department of Thoracic Surgery, First Affiliated Hospital, School of Medicine, Zhejiang University, Hangzhou, China; 8 International Business School, Zhejiang University, Jiaxing, China; 9 Biomedical and Health Translational Research Centre of Zhejiang Province, Jiaxing, China; 10 Department of Hemotology, First Affiliated Hospital, School of Medicine, Zhejiang University, Hangzhou, China

**Keywords:** Non-small-cell lung cancer, Cancer therapeutic resistance

## Abstract

Non-genetic resistance of cancer remains poorly understood in clinical research and practice. To better understand resistant cancer cell heterogeneity, we isolated a novel riboflavin^+^NOTCH1^+^ population from cisplatin-naïve and -resistant lung cancer cell lines and patient specimens with or without immunotherapy and chemotherapy. This population was also identified as *SLC52A2* (one of the riboflavin transporters)^+^*NOTCH1*^*+*^ cells in single-cell RNA sequencing (scRNA-seq) data derived from advanced lung tumors before therapy. Despite its therapy-naïve origin, the population, designated as stably resistant cancer cells (SRCC), exhibited the epithelial state, innate and stable resistance to therapy (chemotherapy, targeted therapy and immunotherapy), cell dormancy, elevated reactive oxygen species (ROS), and anti-apoptotic and anti-ferroptotic survival. These cellular and molecular characteristics distinguished SRCC from other resistant populations, including cancer stem-like cells (CSC), epithelial-mesenchymal transition (EMT) cells, and drug-tolerant persisters (DTP). The non-canonical NOTCH1 pathway, but not the inactivated canonical NOTCH1 pathway, played a critical role in the resistance of SRCC. Specifically, it modulates cell cycle, iron metabolism, EMT, and ferroptosis vulnerability in SRCC at the transcriptional level. It also controls the initiation of ferroptosis in lysosomes via a posttranslational NOTCH1-AKT-BAX axis. Inhibition of the non-canonical NOTCH1 pathway re-sensitizes these dormant and resistant cells to cisplatin-induced cell death in vitro and in vivo, including ferroptosis, apoptosis, and necroptosis. Our study contributes to a deeper understanding of cancer resistance and promotes the development of more effective therapeutic strategies against resistant cancer cells.

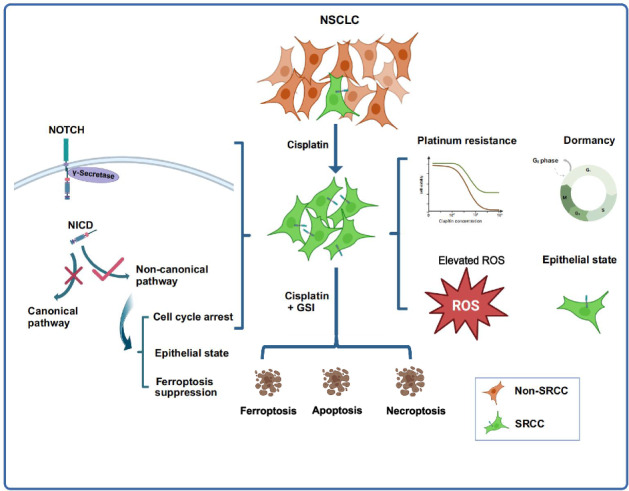

## Introduction

Lung cancer has long been the leading cause of cancer-related mortality, as the five-year survival rate for patients with the stage IV non-small cell lung cancer (NSCLC) is as low as 5.8% [1]. This clinical challenge is largely attributed to therapeutic resistance in cancer [2], which encompasses target mutation resistance [3] and non-target mutation resistance (or non-genetic resistance, reviewed by Marine et al. [4]).

Non-genetic resistance is hypothesized to be associated with heterogeneous resistant cancer cell populations, including CSC [5], EMT and partial EMT cells [6, 7], and DTP [8]. Rare CSC are responsible for tumor initiation and therapeutic resistance [9, 10]. Our research has identified c-Kit^mid^CD3^+^Lin^-^ leukemia stem cells in *Pten*-null T acute leukemia mice, CD166^+^CD49f^hi^CD104^-^Lin^-^ CSC in human NSCLC tumors, and CDC50A^+^Lin^-^ CSC in human ovarian tumors [11–13]. Invasive EMT cells, as well as partial EMT cells, contribute to metastasis, therapeutic resistance, stemness, and the development of immunosuppressive microenvironments [6, 7]. DTP exhibit tolerance to targeted therapy but resume drug sensitivity following the cessation of therapy [8]. This reversible resistance of DTP is associated with the benefits of drug rechallenge in some patients with rapidly relapsed tumors [14]. Conversely, SRCC have recently been proposed to be responsible for the futile effects of drug rechallenge on many other patients with relapsed and resistant tumors [4]. Unfortunately, the SRCC are poorly defined and characterized, partially due to the technical challenges in enriching or purifying the population for functional assessment.

It is established that NOTCH signaling plays a pivotal role in regulating stem cell self-renewal, differentiation and tumorigenesis [15, 16]. For example, NOTCH signaling is hyperactivated in lung adenocarcinoma (LUAD) [17, 18]. The ablation of *NOTCH1* in *Kras*-induced LUAD mice resulted in p53-mediated apoptosis and growth retardation of tumors [19]. However, the role of NOTCH receptors in cancer is highly complex and context-dependent. Specifically, NOTCH signaling functions as a proto-oncogene in LUAD, but as a suppressor in lung squamous carcinoma and small cell lung cancer [16].

NOTCH signaling also confers therapeutic resistance in cancer cells, which eventually leads to cancer relapse. For instance, the overexpression of NOTCH1 or NOTCH3 in LUAD has been demonstrated to induce resistance to EGFR tyrosine kinase inhibitors [20, 21]. Our recent study has demonstrated that canonical and non-canonical NOTCH1 signaling play distinct roles in cancer resistance [12]. The canonical pathway regulates the self-renewal of resistant lung CSC, while the non-canonical pathway modulates their chemoresistance. The distinct roles of NOTCH signaling in other resistance populations remain to be elucidated.

To better understand the heterogeneity of resistant cancer cells, we developed in vitro clinically relevant models for platinum-resistant lung cancer cells and revealed a mixture of distinct resistant cancer populations. Among these populations, a novel riboflavin^+^NOTCH1^+^ population was isolated. This population (or the analogous *SLC52A2*^+^*NOTCH1*^+^ population in scRNA-seq data), was also present in clinical NSCLC specimens before therapy, but innately resistant to chemotherapy and immunotherapy. The riboflavin^+^NOTCH1^+^ cells were determined to be SRCC because they exhibited intrinsic and stable resistance, dormancy, elevated ROS levels and an epithelial phenotype. Our mechanistic investigation demonstrated that, despite the inactivation of the canonical NOTCH1 pathway, the non-canonical NOTCH1 pathway regulated cell cycle, iron metabolism, EMT, and ferroptosis vulnerability in SRCC at the transcriptional level. It also suppresses the initiation of ferroptosis via a posttranslational NOTCH1-AKT-BAX axis. The inhibition of the non-canonical pathway rendered SRCC susceptible to cisplatin-induced cell death. The combination of a γ-secretase inhibitor (GSI) and cisplatin induces ferroptosis, apoptosis, and necroptosis in vitro and in vivo.

## Results

### Riboflavin^+^NOTCH1^+^ cells in lung cancer cell lines and specimens are resistant to therapy

**Fig. 1:**
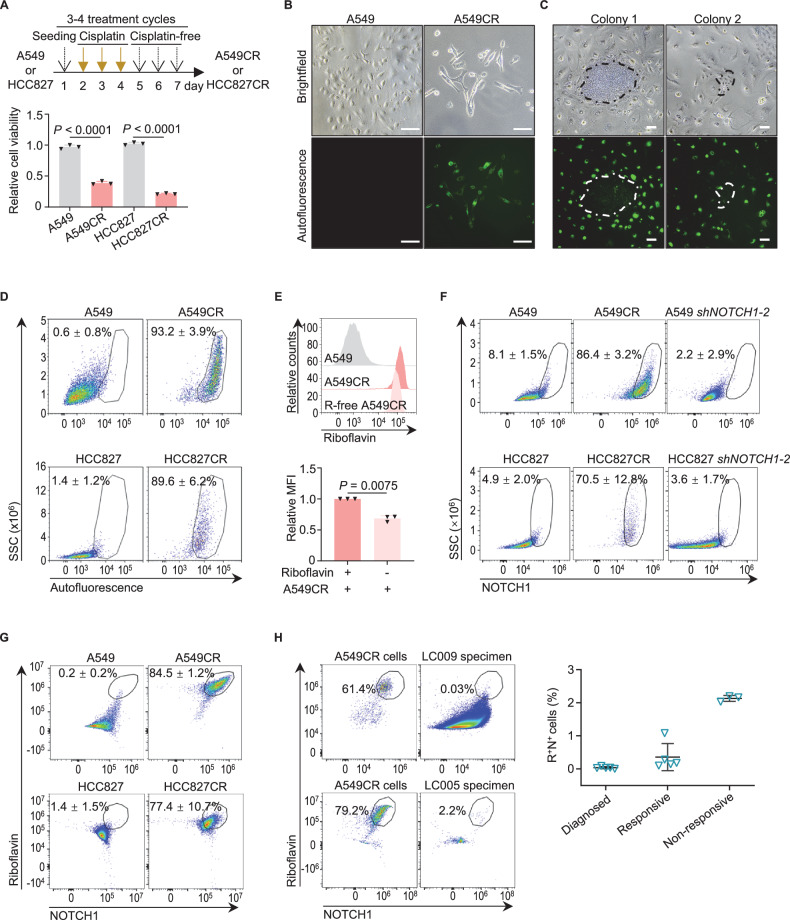
The riboflavin^+^NOTCH1^+^ resistant cancer cells are identified in NSCLC cell lines and specimens. **A** Establishment of in vitro cisplatin-resistant NSCLC models. The models were established according to the schematic diagram in the upper panel and validated with proliferation assays shown in the lower panel (n = 3 independent experiments). See cisplatin response assays for the IC50 of cisplatin in Fig. S1A. **B** Representative images of autofluorescence in A549 and A549CR cells. Scale bar, 100 μm. **C** Representative brightfield and autofluorescence images of cisplatin-exposed A549CR cells cultured without passaging in cisplatin-free medium for 98 days. Colonies are indicated by dashed outlines. Scale bar, 50 μm. **D** Flow cytometric analysis of autofluorescence in A549/A549CR and HCC827/HCC827CR cells (n = 4 independent experiments). Results are summarized in Fig. S1F. **E** Representative flow cytometric analysis and quantification of the riboflavin fluorescence in A549CR cells cultured in riboflavin-containing or -free (or R-free) medium for 28 days (n = 3 independent experiments). MFI, mean fluorescence intensity. The experimental design is shown in Fig. S1H. **F** Flow cytometric analysis of NOTCH1^+^7-AAD^-^ fractions in A549/A549CR and HCC827/HCC827CR cells, and in A549 and HCC287 clones with the sh*NOTCH1-2* knockdown (n = 3 independent experiments). Results are summarized in Fig. S1K. 7-AAD, 7-Aminoactinomycin D. **G** Flow cytometric analysis of the riboflavin^+^NOTCH1^+^7-AAD^-^ population in both A549/A549CR and HCC827/HCC827CR cells (n = 3 independent experiments). Results are summarized in Fig. S1L. **H** Representative flow cytometric analysis (left panel) and quantification (right panel) of the riboflavin^+^NOTCH1^+^7-AAD^-^ (or R^+^N^+^) population in clinical NSCLC specimens before and after therapy (n = 3 or 5 patients per group, Table S1). The responsive group includes patients with a complete pathologic response or a major pathologic response, whereas the non-responsive group encompasses all other treated patients. SSC, side scatter. the data are presented as mean ± standard deviation (SD). *P* values were calculated using Student’s unpaired *t*-tests (**A**), or paired *t*-tests (**E**).

The presence of the distinct resistant cancer cell populations mentioned above argues that heterogeneous resistant cancer cell populations are capable of surviving chemotherapy, targeted therapy, and/or immunotherapy within a tumor. To better elucidate the mechanisms underlying this heterogeneity, we first established clinically relevant platinum resistance models in vitro [22], as outlined in Fig. 1A (upper panel). Few A549 and HCC827 cells survived after 4 cycles of treatment with 20 µmol/L and 10 µmol/L cisplatin, which were equivalent to the half-maximal inhibitory concentrations (IC_50_) for these cells (Fig. S1A), respectively. The majority of surviving A549 (or A549CR) and HCC827 (or HCC827CR) cells showed cisplatin resistance, as indicated by their elevated IC_50_ (Fig. S1A). The resistance of A549CR and HCC827CR cells was not attributable to contamination with other cell lines or mycoplasma [23] (Fig. S1B, C).

A549CR and HCC827CR cells were phenotypically different from their parental cells. As compared to their parental cells, these resistant cells demonstrated a significantly diminished growth rate in both in vitro culture and in vivo grafted immunodeficient mouse model without cisplatin treatment (Fig. 1A, lower panel, and Fig. S1D). Remarkably, these cells also displayed strong autofluorescence (Figs. 1B and S1E). Even after 98 days of cisplatin-free culture without passaging, the vast majority of A549CR cells maintained autofluorescence and growth retardation, in contrast to the reversible DTP [8]. However, very few cells restored proliferation activity and lost autofluorescence, resulting in non-fluorescent colonies with distinct cell density (Fig. 1C). Of the 45 colonies detected, 69% were high-density clumps, suggesting their origin from non-fluorescent DTP-like cells with reversible resistance. The remaining 31% of colonies were of low density, which were possibly attributable to DTP-like clones with weak proliferative capacity or to re-awakened cells that were previously autofluorescent and dormant. These observations suggest that a mixture of resistant cancer cells with distinct characteristics survives after cisplatin treatment (or chemotherapy).

Given that the autofluorescent cancer cells behaved like the previously proposed SRCC [4], we employed stem cell technology to further characterize them. More than 89% of A549CR and HCC827CR cells showed an autofluorescence with an emission peak at 533-550 nm, which was excited by both 405 nm and 488 nm lasers, but not by 561 nm and 640 nm lasers (Figs. 1D and S1F, G). This emission pattern was distinct from the 600 nm emission of the lipofuscin-like red fluorescence observed in senescent cells [24], the 460 nm emission of NADH, and the 348 nm emission of tryptophan in cells [25]. Alternatively, the green autofluorescence resembled the fluorescence of riboflavin and its derivatives, with an emission at 533 nm and excitation by 360-465 nm light [25, 26]. Furthermore, the depletion of the culture supplement riboflavin resulted in a significantly reduced autofluorescence in A549CR cells, despite the increased expression of SLC52A1, a riboflavin transporter [27] (Figs. 1E and S1H, I). Together with the evidence that riboflavin contributes to cisplatin resistance [28, 29], these findings support the hypothesis that the fluorescence of riboflavin and/or its derivatives (hereafter referred to as “riboflavin fluorescence”) accumulates in the resistant cells.

As NOTCH1 was expressed on the surface of A549CR and HCC827CR cells, both the riboflavin fluorescence and NOTCH1 were able to enrich a novel and dominant riboflavin^+^NOTCH1^+^ population in A549CR, HCC827CR, and PC-9CR cells (Figs. 1F, G and S1J–M). Surprisingly, in spite of its rarity, this population was also present in their cisplatin-naïve parental cells. Of particular significance is the isolation of the riboflavin^+^NOTCH1^+^ population, which ranged from 0.004% to 2.20%, from the clinical specimens of 13 NSCLC patients, including 8 patients undergoing immunotherapy and chemotherapy (or targeted therapy) and 5 diagnosed (untreated) patients (Fig. 1H and Table S1). The roughly 100-fold increase in the riboflavin^+^NOTCH1^+^ fraction derived from non-responsive NSCLC tumors suggests that this population is resistant to both immunotherapy and chemotherapy. In addition, the riboflavin^low^NOTCH1^low^ population of unknown function was also detected in A549CR and PC-9CR cells and certain tumor specimens. A small fraction of riboflavin^-^NOTCH1^-^ cells likely contributed to the expansion of high-density colonies in cisplatin-free culture for 98 days (Fig. 1C). Taken together, riboflavin^+^NOTCH1^+^ cells represent a resistant population in lung cancer cell lines and specimens regardless of immunotherapy and chemotherapy.

### Riboflavin^+^NOTCH1^+^ cells display intrinsic and stable resistance, dormancy and oxidative stress

**Fig. 2:**
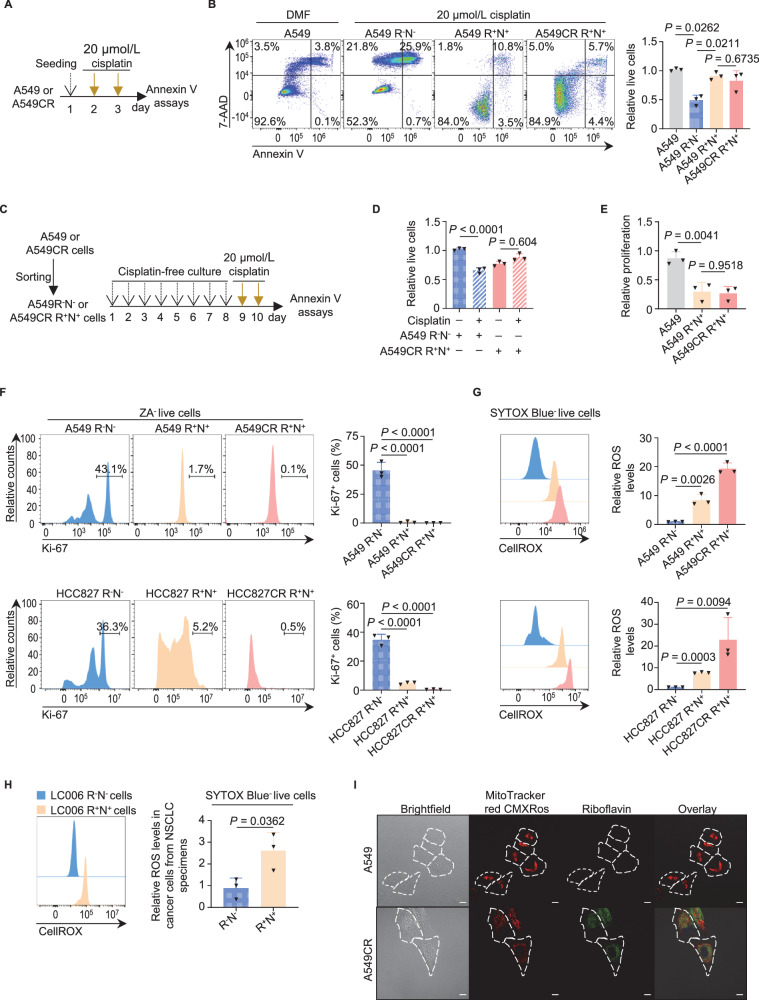
Both chemotherapy-naïve and chemotherapy-exposed riboflavin^+^NOTCH1^+^ populations exhibit stable chemoresistance, cellular dormancy, and elevated ROS. **A** Schematic design for investigating the effect of cisplatin on the cisplatin-naïve and cisplatin-exposed riboflavin^+^NOTCH1^+^ populations derived from A549 and A549CR cells, respectively. **B** Representative Annexin V assays (left panel) and quantification (right panel) for the response of the cisplatin-naïve and cisplatin-exposed riboflavin^+^NOTCH1^+^ populations to cisplatin (n = 3 independent experiments). DMF (N, N-dimethylformamide) served as the diluent for cisplatin. **C,** **D** Assessment (**D**) of the irreversible chemoresistance of riboflavin^+^NOTCH1^+^ A549CR cells after 8 days of cisplatin-free culture (n = 3 independent experiments), according to the schematic design (**C**). Please also see representative Annexin V assays in Fig. S2A. **E** CCK-8 assays for assessing the proliferation of 1 × 10^3^ cisplatin-naïve and cisplatin-exposed riboflavin^+^NOTCH1^+^ cells sorted from the cell lines A549 and A549CR, respectively (n = 3 independent experiments). **F** Representative protein-flow analysis (left panel) and quantification (right panel) with an anti-Ki-67 antibody for the Ki-67^+^ cycling fractions in cisplatin-naïve and -exposed riboflavin^+^NOTCH1^+^ cells (n = 3 independent experiments). **G** Representative flow cytometric analyses (left panel) and quantification (right panel) of ROS levels in cisplatin-naïve and -exposed riboflavin^+^NOTCH1^+^ cells (n = 3 independent experiments). **H** Representative flow cytometric analysis (left panel) and quantification (right panel) of relative ROS levels in the riboflavin^+^NOTCH1^+^ or riboflavin^-^NOTCH1^-^ populations from NSCLC specimens treated with chemotherapy and immunotherapy (n = 3 independent experiments). **I** Representative confocal images of mitochondria in A549 and A549CR cells, which were stained with MitoTracker red CMXRos. The fluorescence of riboflavin is shown in green and that of mitochondria in red. R^-^N^-^ riboflavin^-^NOTCH1^-^; R^+^N^+^, riboflavin^+^NOTCH1^+^; ZA, Zombie Aqua; scale bar, 5 µm; error bars, mean ± SD. *P* values were calculated using one way ANOVA with Tukey’s tests (**B**, **D**–**G**) or Student’s unpaired *t*-tests (**H**).

Our previous study has demonstrated that lung CSC are capable of intrinsic resistance to cisplatin [12]. In contrast, DTP hold inducible and reversible resistance [8]. It was thus of interest to characterize the riboflavin^+^NOTCH1^+^ populations isolated from untreated lung cancer cells (hereafter referred to as the cisplatin-naïve population), and the surviving cells after being exposed to 4 cycles of cisplatin treatment (hereafter referred to as the cisplatin-exposed population). As anticipated, both populations showed comparable resistance to cisplatin-induced cytotoxicity (Fig. 2A, B), thereby substantiating the notion that the riboflavin^+^NOTCH1^+^ populations possess intrinsic resistance, independent of cisplatin pretreatment. This intrinsic resistance was sustained in riboflavin^+^NOTCH1^+^ sorted cells, even after 8 or 73 days of cisplatin-free culture (Figs. 2C, D and S2A, B). The intrinsic and sustained resistance distinguished the riboflavin^+^NOTCH1^+^ populations from the resistance-reversible DTP [8, 30].

In accordance with the observed growth retardation of A549CR and HCC827CR cells, the cisplatin-naïve and cisplatin-exposed riboflavin^+^NOTCH1^+^ populations sorted respectively from A549 and A549CR cells proliferated approximately 3-fold slower than A549 cells (Fig. 2E). The slow proliferation in riboflavin^+^NOTCH1^+^ populations was attributed to cell cycle arrest at G0 phase, as evidenced by their little expression of the cycling marker Ki-67 and lack of incorporation of the thymidine analogue 5-ethynyl-2′-deoxyuridine (EdU, Figs. 2F and S2C). These findings indicate that the riboflavin^+^NOTCH1^+^ cells, irrespective of their cisplatin-naïve or cisplatin-exposed origin, are in a state of dormancy or quiescence.

Given that CSC maintain low ROS levels for their survival and radioresistance [31], we next investigated ROS levels in the riboflavin^+^NOTCH1^+^ populations. It was unexpected that in contrast to CSC with low ROS, riboflavin^+^NOTCH1^+^ cells exhibited approximately 13.5–44.7 times higher ROS levels than riboflavin^-^NOTCH1^-^ cells (Fig. 2G). The increased ROS levels were also observed in riboflavin^+^NOTCH1^+^ cells derived from clinical NSCLC tumors exposed to chemotherapy and immunotherapy (Fig. 2H). This oxidative stress did not appear to result from accumulated ferrous ions labeled with FerroOrange dye, in riboflavin^+^NOTCH1^+^ cells, because the excess iron was stored predorminantly in lysosomes, a safe hub for ferrous ions [32] (Fig. S2D, E). Nevertheless, oxidative stress did not impair mitochondria, which showed the clear fluorescence signals of MitoTracker red CMXRos (Fig. 2I). Taken together, the riboflavin^+^NOTCH1^+^ populations exhibited functional characteristics that were distinct from both CSC and DTP, but similar to the proposed SRCC [4].

### Resistant and dormant programs are activated in SRCC

**Fig. 3:**
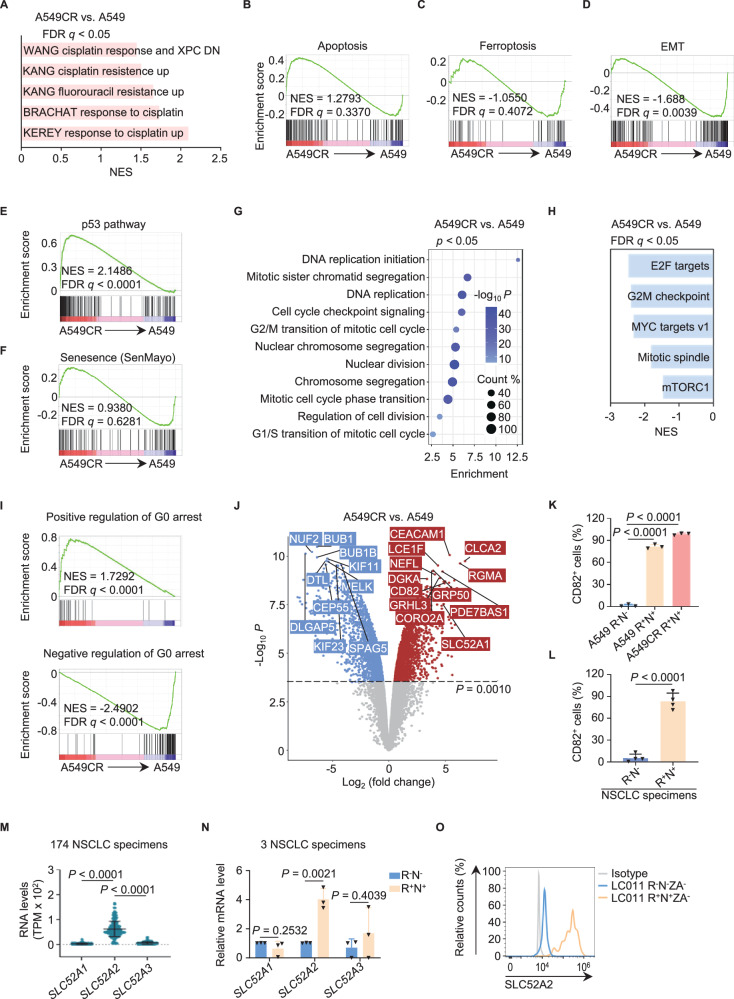
The molecular programs of platinum resistance, EMT and dormancy are activated in riboflavin^+^NOTCH1^+^ SRCC. **A** enrichment of the chemoresistance program for A549CR cells, but not for A549 cells, was determined by GSEA with multiple chemoresistance signatures (n=3 independent experiments for Fig.3A-J, Table S2). **B,** **C** GSEA plots illustrated no enrichment of apoptosis (**B**) and ferroptosis (**C**) regulatory programs for A549CR cells and A549 cells. **D** GSEA demonstrated that the EMT program was correlated with A549 cells, but not with A549CR cells. **E,** **F** GSEA revealed that the p53 pathway (**E**) was positively enriched in A549CR cells, whereas the SenMayo senescence program (**F**) was not. **G** Gene ontology (GO) analysis of DEG revealed a downregulation of cell cycle programs in A549CR cells compared to A549 cells. **H** GSEA demonstrated that the cell cycle regulatory programs were inhibited in A549CR cells, but not in A549 cells. **I** GSEA plots showed a significant correlation of the G0 program with A549CR cells, but not with A549 cells. **J** Volcano plot illustrated the significantly upregulated (red) and downregulated (blue) genes in a comparison between A549CR and A549 cells. A dashed line represents the threshold of *p* < 0.0010 for the DEG. The top 10 DEG are labeled with their gene names. See also the top 10 DEG in Table S4. **K** Flow cytometric quantification of the CD82^+^ fractions in the cisplatin-naïve and cisplatin-exposed riboflavin^+^NOTCH1^+^7-AAD^-^ cells (n = 3 independent experiments). See representative flow cytometric analyses in Fig. S3F. **L** Flow cytometric quantification of the CD82^+^ fractions in the riboflavin^-^NOTCH1^-^7-AAD^-^ and riboflavin^+^NOTCH1^+^7-AAD^-^ cells from NSCLC specimens treated with immunotherapy and chemotherapy (n = 4 independent experiments). See representative flow cytometric analysis in Fig. S3G. **M** Assessment of the mRNA levels of *SLC52A1*, *SLC52A2* and *SLC52A3* in the RNA-seq dataset phs000178 derived from 174 NSCLC tumors. **N** RT-PCR analysis detected the mRNA levels of *SLC52A1*, *SLC52A2*, *SLC52A3* in riboflavin^+^NOTCH1^+^ and riboflavin^-^NOTCH1^-^ cells sorted from the clinical NSCLC specimens LC012, LC013, and LC014 (n = 3 independent experiments). **O** Protein-flow analysis with an anti-SLC52A2 antibody detected SLC52A2 expression in riboflavin^+^NOTCH1^+^ZA^-^ and riboflavin^-^NOTCH1^-^ZA^-^ cells gated from the clinical NSCLC specimen LC011 (n = 1 independent experiment). R^-^N^-^, riboflavin^-^NOTCH1^-^; R^+^N^+^, riboflavin^+^NOTCH1^+^; ZA, Zombie Aqua; TPM, transcripts per million; error bars, mean ± SD. *P* values were calculated using one way ANOVA with Tukey’s tests (**K**) or Student’s unpaired *t*-tests (**L,** **N**). The adjusted *P* values were calculated using Wald tests with the Benjamini-Hochberg correction in DEseq2 (**M**).

In order to gain insight into the molecular regulation of SRCC, bulk RNA-sequencing (RNA-seq) analysis was employed to compare the transcriptomic differences between A549CR cells and A549 cells, as the majority of A549CR cells were riboflavin^+^NOTCH1^+^ right after cisplatin selection. In accordance with the observed stable resistance of SRCC, the gene set enrichment analysis (GSEA) of the RNA-seq data revealed the activation of chemoresistance programs [33–36] in A549CR cells (Fig. 3A and Table S2). Consistently, 126 differentially expressed genes (DEG) in SRCC were implicated in these chemoresistance programs [37] (Fig. S3A and Table S3). The resistance and survival of SRCC with elevated ROS apparently benefited from the repression of apoptotic and ferroptotic programs, which was revealed by GSEA, DEG analysis (e.g., *GSN, HSPB1*, and *FSP1*), and protein-flow analysis for FSP1 (Figs. 3B, C and S3B, C). Inhibition of the EMT program (Fig. 3D) also contributed to the reduced vulnerability of SRCC to ferroptosis [38]. These observed survival mechanisms are of vital importance for the resistance of SRCC.

Bulk RNA-seq analysis also revealed that the dormancy of SRCC is attributed to the suppression of cell cycle programs. GSEA revealed a positive correlation of the p53 pathway with A549CR cells (Fig. 3E, Table S2). The p53 pathway involving *p21* was not associated with the inactivated apoptotic program [39] (Fig. 3B, C), and the inactivated senescence program without *p16* expression [40, 41], but with the suppression of cell cycle machinery (Figs. 3F and S3D). The suppressed cell cycle programs included cell cycle checkpoints (G1/S and G2/M checkpoints), DNA replication, and mitotic controls (mitotic spindle, chromosome segregation, nuclear division, and cell division), as well as repressed positive regulators (e.g., E2F, c-myc, and mTORC1) [42–44] and activated negative regulators (e.g., p53 and *p21*) (Figs. 3E, G, H, and S3D). Furthermore, the dormant state of Ki-67^-^ SRCC observed above was corroborated by the transcriptomic correlation with the G0 quiescence program [45], and the mRNA and protein expression of the dormancy marker CD82 [46, 47] in the SRCC derived from A549, A549CR, and NSCLC specimens (Figs. 3I-L, S3E-G, and Tables S2, S4). These molecular findings served to reinforce the hypothesis that SRCC reside in a state of dormancy or quiescence.

Riboflavin import is mediated by the SLC52A1-3 transporter family in most cells [27] and by ABCG2 in CSC [29]. Interestingly, in contrast to the decreased expression of *ABCG2* (Fig. S3H), the mRNA and protein expression of the gene *SLC52A1* was notably elevated in A549CR and HCC827CR cells (Figs. 3J and S1H, S3I, J). Similarly, the *SLC52A2* gene was highly expressed in A549CR and HCC827CR cells, as well as in chemotherapy-treated lung tumors (Figs. 3M, S3I, J and Table S5). These results indicate that the SLC52A1-3 transporters, rather than ABCG2, play an important role in the observed accumulation of riboflavin in SRCC (Figs. 1B and S1E). More importantly, the heightened expression of *SLC52A2* was positively correlated with riboflavin accumulation in sorted SRCC, in contrast to non-SRCC (Fig. 3N, O). The accumulated riboflavin (and derivatives) appeared to be crucial for the survival of SRCC with elevated ROS, as the riboflavin derivatives flavin adenine dinucleotide and flavin mononucleotide function as cofactors for essential enzymes in the ROS-generating mitochondrial respiratory chain (i.e., respiratory chain complexes I and II) and in two parallel antioxidant pathways: TXNRD and GSR in the glutathione [GSH]-GPX4 pathway, and CoQ6, DHODH, and FSP1 in the coenzyme Q10 [CoQ10]/vitamin K-FSP1 pathway [48]. Among these genes, *CoQ6* and *FSP1* were significantly upregulated in A594CR cells (Fig. S3K).

### *SLC52A2*^*+*^*NOTCH1*^*+*^ cells are the SRCC population detected by scRNA-seq analysis

**Fig. 4:**
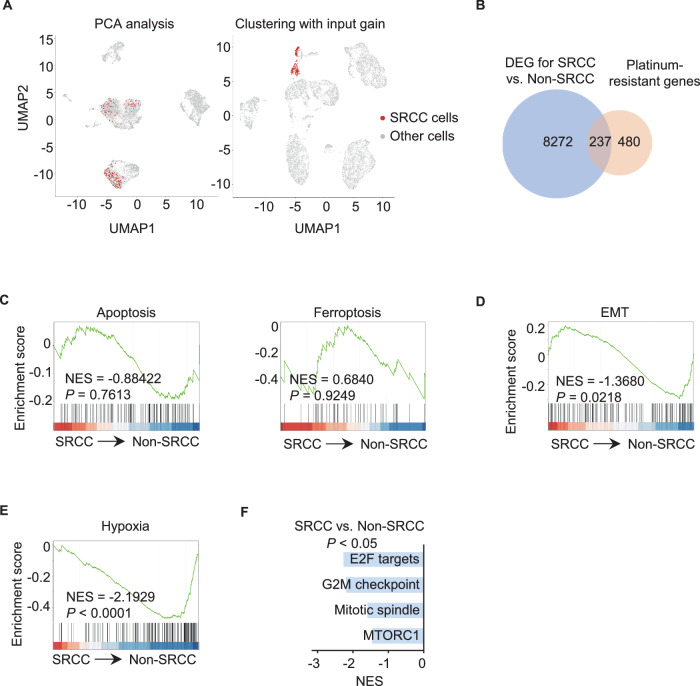
The *SLC52A2*^*+*^*NOTCH1*^*+*^ SRCC population in diagnosed NSCLC specimens is identified using scRNA-seq analysis. **A** Clustering of CNV^+^ cancer cells in the GSE131907 dataset using PCA analysis (left panel) and modified PCA analysis with *SLC52A2* and *NOTCH1* expression pre-amplified 5 folds (right panel). See also PCA clustering analysis and inferCNV analysis of total cells in the GSE131907 dataset in Fig. S4A–D, and analytic pipeline for modified PCA clustering analysis with input gain (Fig. S4G). **B** Venn diagram illustrating the association of 237 DEG with platinum resistance (Table S3). **C** GSEA using the tools *GSEABase* and *fgsea* showed no enrichment of apoptotic and ferroptotic programs in SRCC, as compared to non-SRCC. **D**–**F** GSEA using the tools *GSEABase* and *fgsea* showed that the EMT (**D**), hypoxia (**E**), and cell cycle (**F**) regulatory programs were significantly suppressed in SRCC, as compared to non-SRCC. NES normalized enrichment score.

We next wanted to characterize the population using the scRNA-seq dataset GSE131907 derived from 44 diagnosed LUAD tumors at advanced stages [49]. However, principal component analysis (PCA), as well as the rare cell analysis tools CellSIUS, EDGE, and scCAD [50], failed to enrich the SRCC population (e.g., the *SLC52A2*^*+*^*NOTCH1*^*+*^ population) from 208,506 cells or 11,763 cancer cells harboring copy number variation (CNV) on uniform manifold approximation and projection plots (Fig. 4A, left panel, Fig. S4A–F, Table S6).

In order to address this difficulty in cancer cell state clustering [51], the PCA clustering technique was implemented with an input gain setting (e.g., the preamplified expression of *SLC52A2* and *NOTCH1* by 5 folds), which effectively improved the clustering of rare populations (Fig. S4G, see technical details in the Methods section). As a result, 285 *SLC52A2*^*+*^*NOTCH1*^*+*^ cells were clustered from CNV^+^ cancer cells in GSE131907, representing 65.2% of all the *SLC52A2*^*+*^*NOTCH1*^*+*^ cells selected by Seurat’s *WhichCells* module (Fig. 4A, right panel, Fig. S4H, I, Table S7). The population constituted roughly 0.1% of total cells, which was in the fractional range of riboflavin^+^NOTCH1^+^ SRCC detected in NSCLC samples (Fig. 1H).

As compared to 2,115 *SLC52A2*^*-*^*NOTCH1*^*-*^ non-SRCC, the *SLC52A2*^*+*^*NOTCH1*^*+*^ SRCC cluster was correlated with 237 platinum resistance genes [37] (Fig. 4B and Table S3), but not with the apoptotic and ferroptotic programs (Fig. 4C and Table S2), the pro-ferroptosis EMT program (Fig. 4D) and the hypoxic state (Fig. 4E). The dormant cluster with *CD82* expression in 53.3% of cells, experienced the repression of cell cycle regulatory programs, including E2F- and mTORC1-mediated pathways, the G2M checkpoint and the mitotic spindle checkpoint (Figs. 4F, S4I and Table S7). With these comparable functions, the *SLC52A2*^*+*^*NOTCH1*^*+*^ cluster is analogous to the riboflavin^+^NOTCH1^+^ SRCC population.

### Non-canonical NOTCH1 signaling suppresses the vulnerability of SRCC to ferroptosis

**Fig. 5:**
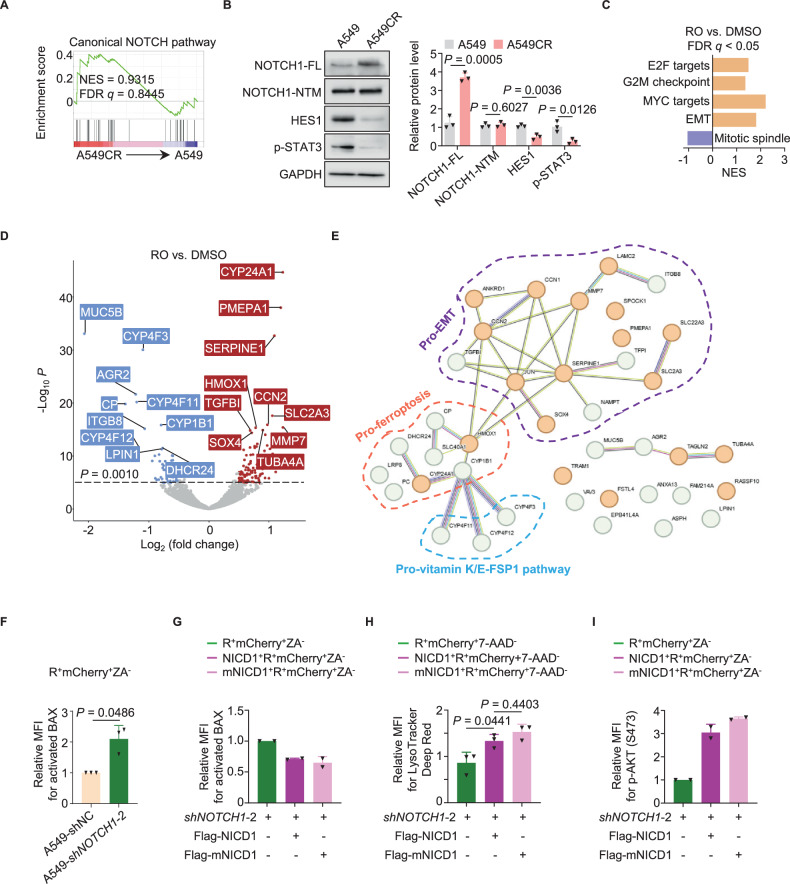
Non-canonical NOTCH signaling regulates the cell cycle, EMT and ferroptosis vulnerability of SRCC. **A** GSEA of bulk RNA-seq data demonstrated that the canonical NOTCH pathway was not enriched in A549 and A549CR cells (n = 3 independent experiments). **B** Representative Western blot analysis (left panel) and quantification (right panel) of key regulators in the canonical NOTCH1 pathway (n = 3 independent experiments). FL, full-length NOTCH1; NTM, NOTCH1 transmembrane and intracellular region. **C** GSEA revealed the activation of cell cycle and EMT programs in A549CR cells in response to RO treatment. **D** Volcano plot showed the upregulated (red) and downregulated (blue) genes in RO- vs. dimethyl sulfoxide (DMSO)-treated A549CR cells. The dashed line represents the significance threshold of *p* < 0.0010. Please see a list of the top 10 DEG in Table S4. **E** STRING protein network analysis revealed protein-protein interactions between the proteins encoded by the top 20 upregulated (orange bubbles) and downregulated (green bubbles) genes in A549CR cells in response to RO treatment. The 3 groups of genes highlighted by dashed circles are associated with pro-EMT, pro-ferroptotic, and pro-vitamin-K/E-FSP1 functions. **F** Protein-flow analysis with the monoclonal antibody 6A7 for BAX activation in riboflavin^+^mCherry^+^ZA^-^ SRCC gated from A549-shNC and A549-*shNOTCH1*-2 cells (n = 3 independent experiments). **G** Protein-flow analysis with the monoclonal antibody 6A7 for BAX activation in riboflavin^+^mCherry^+^ZA^-^ SRCC gated from A549-*shNOTCH1-2* cells with the overexpression of flag-tagged NICD1 or mNICD1 (n = 3 independent experiments). Both A549-*shNC* and A549-*shNOTCH1-2* were positive for mCherry. **H** Flow cytometric analysis for the fluorescence of LysoTracker Deep Red in riboflavin^+^mCherry^+^7-AAD^-^ SRCC gated from A549-*shNOTCH1-2* cells with the overexpression of flag-tagged NICD1 or mNICD1 (n = 3 independent experiments). **I** Protein-flow analysis with the monoclonal antibody SY28-05 for AKT phosphorylation (S473) in riboflavin^+^mCherry^+^ZA^-^ SRCC gated from A549-*shNOTCH1-2* cells with the overexpression of flag-tagged NICD1 or mNICD1 (n = 2 independent experiments). Both A549-shNC and A549-*shNOTCH1-2* are positive for mCherry. ZA, Zombie Aqua fluorescence for dead cells; RO, RO4929097; error bars, mean ± SD. *P* values were calculated using Student’s unpaired *t*-tests (**B**), Student’s paired *t*-tests (**F**), or one way ANOVA with Tukey’s tests (**H**).

Our previous study indicates that NOTCH1 plays a non-canonical role in the platinum resistance of lung CSC [12]. Therefore, it was of great interest to investigate the role of NOTCH1 in the regulation of SRCC. Although the mRNA and protein expression of NOTCH1 was increased in most A549CR cells (Figs. 1F, and S5A), GSEA and Western blot analysis revealed that the canonical NOTCH pathway was not activated in SRCC derived from A549CR, HCC827CR, and tumors with scRNA-seq data (Figs. 5A, B, and S5A–C). NOTCH1 did not appear to regulate SRCC through the canonical pathways of other NOTCH receptors, because the sh*NOTCH1* knockdown had little effect on the expression of NOTCH2, NOTCH3 and NOTCH4 on the surface of SRCC (Fig. S5D). These observations suggest a role of the non-canonical NOTCH1 pathway in the survival and resistance of SRCC [52].

To elucidate its non-canonical functions, we suppressed the protein levels of cleaved NOTCH1 intracellular domain (or NICD1) in SRCC for bulk RNA-seq analysis, using the GSI RO4929097 (or RO) [53] (Fig. S5E, F). Consistent with the decreased levels of full-length NOTCH1 on the surface of treated SRCC, the two-day RO treatment did not activate the canonical NOTCH pathway (Fig. S5G, H). Despite the restoration of some cell cycle processes, the inhibition of the non-canonical NOTCH pathway was not sufficient to re-awaken dormant SRCC (Fig. 5C). Furthermore, although NOTCH1 inhibition by RO did not cause cell death including ferroptosis (Fig. S5I, J), it did enhance ferroptosis vulnerability at the transcriptional level by promoting the activation of the pro-ferroptotic EMT program (e.g., upregulated *SOX4* and *SERPINE1*), the accumulation of ferrous ions (e.g., upregulated *HMOX1* and downregulated *SLC40A1*), and the impairment of the GSH-GPX4 antioxidant pathway (e.g., upregulated *CYP24A1* and downregulated *LRP8*, Fig. 5D, E, Table S4) [38, 54, 55]. The enhanced ferroptosis vulnerability resulting from NOTCH1 inhibition was not associated with the expression control of the key ferroptosis regulators GPX4, FSP1, and ACSL4 (Fig. S5K). The absence of ferroptosis in RO-treated SRCC was not attributable to an inactive tetrahydrobiopterin (BH4)-DHFR antioxidant pathway or the intact lipid peroxidation promoter ACSL4, but rather to an active CoQ10/vitamin K-FSP1 antioxidant pathway [54, 56, 57] (Figs. 5E and S5K-M).

We next sought to investigate the posttranslational crosstalk of NICD1 with other signaling pathways in the regulation of ferroptosis. It is recently suggested that ferroptosis is initiated in lysosomes through two discrete and integrative events: (1) lysosomal lipid peroxidation, which triggers ferroptosis; (2) lysosomal membrane permeabilization (LMP), which permits the release of ferrous ions into the cytosol [58, 59]. In light of the detection of iron accumulation in the lysosomes of SRCC, the role of NICD1 in LMP was investigated. NOTCH1 inhibition via sh*NOTCH1* knockdown resulted in a noteable increase of activated BAX, a critical “gate opener” for iron-releasing LMP [60], in SRCC (Fig. 5F). However, the increase of BAX activation was impeded by ectopically expressing Flag-tagged NICD1 or NICD1 with the deletion of the RBPJ-binding RAM domain, the mutant which lost the canonical transactivating activity of NOTCH1 (or mNICD1, Fig. 5G). Apparently, mNICD1 inhibited BAX activation and BAX-mediated LMP (Fig. 5H). Given the inhibition of BAX by AKT [61], NOTCH1 suppressed the BAX-mediated LMP in SRCC via activating AKT in a non-canonical manner (Fig. 5I). Taken together, the non-canonical NOTCH1 pathway suppresses ferroptosis vulnerability via both transcriptional inhibition of lipid peroxidation and posttranslational inhibition of BAX-mediated LMP.

### Combinatorial treatment with GSI and cisplatin induces ferroptosis and apoptosis in dormant SRCC

**Fig. 6:**
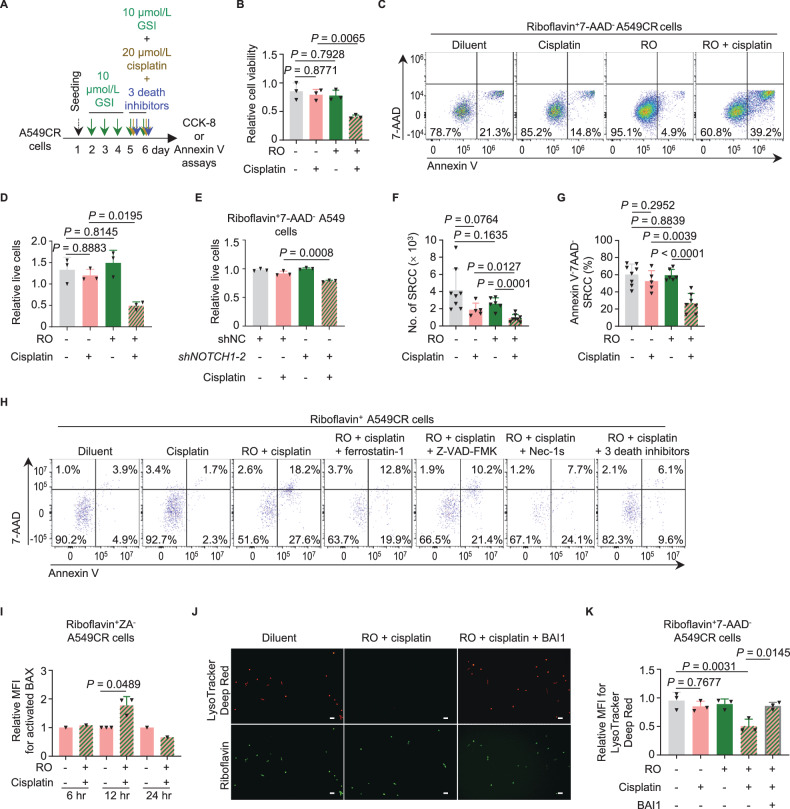
Inhibition of the non-canonical NOTCH1 pathway re-sensitizes SRCC to cisplatin. **A** Schematic design of the combinatorial treatment with GSI and cisplatin. **B** Viability of A549CR cells in response to cisplatin, RO, or both was assessed using CCK-8 assays (n = 3 independent experiments). **C**, **D** Representative Annexin V assay (**C**) and quantification (**D**) for riboflavin^+^7-AAD^−^ SRCC in response to cisplatin, RO, or both (n = 3 independent experiments). **E** Annexin V assays for the effect of cisplatin on riboflavin^+^7-AAD^-^ A549-shNC and A549-*shNOTCH1-2* cells (n = 3 independent experiments). See also representative assays in Fig. S6B. **F** Assessment of the absolute number of riboflavin^+^7AAD^−^ SRCC in xenograft tumors treated with RO alone, cisplatin alone, or both (n = 5–8 mice per group). **G** Assessment of the Annexin V^-^7AAD^-^ live cell fraction within the riboflavin^+^ SRCC compartment derived from xenograft tumors treated with RO alone, cisplatin alone, or both (n = 5–8 mice per group). **H** Representative Annexin V assay for the effect of the ferroptosis inhibitor ferrostatin-1 (10 µmol/L), the apoptosis inhibitor Z-VAD-FMK (10 µmol/L), and the necroptosis inhibitor Nec-1s (10 µmol/L) on the death of riboflavin^+^7-AAD^-^ SRCC induced by the combinatorial treatment of RO and cisplatin (n = 2 independent experiments). **I** Protein-flow analysis with the monoclonal antibody 6A7 for BAX activation in riboflavin^+^ZA^-^ A549CR cells in response to 6-, 12-, or 24-hour treatment with RO and cisplatin (n = 1–3 independent experiments). **J**, **K** Assessment of the lysosomal integrity of riboflavin^+^ SRCC treated with RO and cisplatin or with RO, cisplatin, and BAI1 for 24 hours. The lysosomal integrity is determined using fluorescent microscopy (**J**, n = 1 independent experiment) or flow cytometry (**K**, n = 3 independent experiments) with the dye LysoTracker Deep Red. Scale bar, 50 μm; ZA, Zombie Aqua fluorescence for dead cells; RO, RO4929097; error bars, mean ± SD. *P* values were calculated by one way ANOVA with Tukey’s tests (**B**, **D**, **E**, **K**), Student’s unpaired *t*-tests (**F**, **G**) or Student’s paired *t*-tests (**I**).

Recent reports have suggested a potential role for cisplatin in ROS production and lipid peroxidation [62–65]. Given the inhibitory role of NOTCH1 in the BAX-mediated LMP, it seems plausible to hypothesize that a combination of cisplatin with NOTCH1 inhibtion may be sufficient to initiate ferroptosis (and/or other types of cell death) in dormant SRCC with elevated ROS (Fig. 6A). Indeed, the combination of cisplatin with RO, PF-03084014 (or PF) [66] or *shNOTCH1*, resulted in the reduced viability of A549CR cells and the increased death of riboflavin^+^7-AAD^-^ SRCC (Figs. 6B–E, and S6A, B). Conversely, neither NOTCH1 inhibiton nor cisplatin treatment alone demonstrated a comparable effect. The in vitro observations were further corroborated by in vivo xenograft assays with a combination of RO and cisplatin (Figs. S6C, D and 6F, G).

Cell death in response to GSI and cisplatin was further characterized with the ferroptosis inhibitor ferrostatin-1, the apoptosis inhibitor Z-VAD-FMK, and the necroptosis inhibitor necrostatin-1s (Nec-1s), as well as specific markers for the respective forms of cell death. The combined treatment resulted in ferroptosis (also indicated by oxidized BODIPY 581/591 C11), apoptosis (NucView 488 fluorescence), and necroptosis (MLKL phosphorylation at Serine 358) in the riboflavin^+^ SRCC of A549CR and PC-9CR (Figs. 6H, and S6E–K). These results indicate that the combinatorial therapy of GSI and platinum drugs is an effective approach to target SRCC via inducing cell death.

The molecular mechanisms underlying the initiation of ferroptosis by the combination of RO and cisplatin were further characterized. The treatment with RO and cisplatin resulted in BAX activation and LMP (Figs. 6I–K and S6L). The induced LMP was inhibited by the BAX inhibitor BAI1 (Fig. 6J, K), thereby supporting the notion that RO induces BAX-mediated LMP via the non-canonical NOTCH1-AKT-BAX axis. In the presence of RO, the ferroptosis-inducing efficacy of cisplatin was surprisingly comparable to that of a combination of the GPX4 inhibitor RSL3 and the FSP1 inhibitor FSEN1, but much more potent than that of RSL3 or FSEN1 alone (Fig. S6M-Q). In other words, in the presence of NOTCH1 inhibition, cisplatin or a combination of RSL3 and FSEN1, is sufficient to suppress both GPX4-mediated and FSP1-mediated antioxidant pathways in SRCC, resulting in lysosomal lipid peroxidation and ferroptosis. These mechanistic observations provide insight into the mechanism(s) underlying the induction of ferroptosis by a combination of GSI and cisplatin.

## Discussion

This study has identified a novel riboflavin^+^NOTCH1^+^ SRCC population in lung cancer cell line and clinical specimens. It is observed at a low frequency before cisplatin treatment or therapy, but highly enriched after cisplatin treatment or therapy (chemotherapy and immunotherapy). An equivalent *SLC52A2*^*+*^*NOTCH1*^*+*^ population is present in scRNA-seq data from NSCLC specimens without therapy. Notably, SRCC exhibit a surprising combination of inherent and stable resistance, the epithelial characteristics, cellular dormancy, pro-survival programs, and elevated ROS levels. The non-canonical NOTCH1 pathway, but not the canonical NOTCH1 pathway, plays an important role in the transcriptional regulation of cell cycle, iron metabolism, EMT, and ferroptosis vulnerability in SRCC. It also suppresses the initiation of ferroptosis via the non-canonical NOTCH1-AKT-BAX axis. The inhibition of the non-canonical NOTCH1 pathway renders SRCC susceptible to platinum-induced ferroptosis, apoptosis, and necroptosis in vitro and in vivo.

The SRCC population is distinct from the previously reported resistant populations including EMT cells, CSC, and DTP. The distinction between SRCC and EMT cells lies in their respective epithelial and mesenchymal states. The former manifests an epithelial state, whereas the latter exhibits a mesenchymal state. While both SRCC and CSC share the intrinsic resistance, there are three notable distinctions between the 2 populations: 1) elevated ROS levels are observed in SRCC, but not in CSC [31]; 2) SRCC reside in deeper dormancy than slow-cycling lung CSC [12], and grow at a much slower rate than CSC in sphere-forming culture (data not shown); 3) the canonical NOTCH1 pathway is activated in CSC [12], but not in SRCC. The epithelial SRCC with stable resistance also differ from the mesenchymal DTP [30], whose resistance is inducible and reversible [8, 67]. It would be of significant interest to investigate how these resistant populations within a tumor collectively contribute to cancer resistance and relapse.

Dormant cancer cells are recently proposed to be a primary contributor to metastasis or relapse 5 years after curative therapy [68, 69]. However, the accurate detection and quantification of these cells is very challenging, mainly due to the lack of highly sensitive dormancy markers and single-cell resolution technology [69]. SRCC are rare (as low as 0.004%) and dormant. If SRCC constitute the major dormant cancer population (or one of them), riboflavin fluorescence (or SLC52A1-3), NOTCH1, and CD82, may serve as reliable markers for the clinical assessment of tumor cell dormancy and prediction of relapse. The observation that SRCC appeared to be re-awakened after 98 days of cisplatin-free culture supports the role of dormant cancer cells in long-latency relapse. It is important to elucidate the mechanisms underlying SRCC re-awakening. As the inhibition of NOTCH signaling alone is not sufficient to fully awaken dormant SRCC, additional molecular event(s) is required for their complete awakening. As its reported role in the re-awakening of dormant breast cancer cells carrying the *β1-integrin* disruption [70], the p53-p21 axis activated in SRCC is a promising candidate for further investigation.

NOTCH1 is highly expressed on the surface of resistant SRCC, CSC, and EMT cells in lung tumors [12, 71]. However, it is quite surprising that, in contrast to CSC and EMT cells, only the non-canonical NOTCH1 pathway is activated in SRCC. This surprising bias may be important for the suppression of EMT, which is promoted by the canonical pathway [72]. While both SRCC and CSC require the non-canonical NOTCH1 pathway for their resistance [12], further investigation is necessary to elucidate whether they share a non-canonical program. At least, AKT is identified as a suppressive mediator of both ferroptosis in lung SRCC and apoptosis in lung CSC via the non-canonical NOTCH1 pathway [12].

Several therapeutic strategies have been proposed for targeting dormant cancer cells, including the direct eradication of the cells or the reactivation (re-sensitization) of them to current therapies [69]. For example, a combinatorial trial with the CDK4/6 inhibitor abemaciclib and the autophagy inhibitor hydroxychloroquine (NCT04523857) has been designed to eliminate dormant breast cancer cells [73]. The present study proposes a novel dormancy-targeting strategy in which the combination of chemotherapy with NOTCH1 inhibition (e.g., GSI or NOTCH1-specific antibodies for alleviated intestine toxicity), induces cell death in dormant SRCC. A notable advantage of this strategy is its potential to target both CSC and SRCC [12], or even all resistant populations in heterogeneous tumors.

## Materials and methods

### Chemicals

The chemicals and compounds cisplatin (#HY-17394), RO4929097 (#HY-11102), PF-03084014 (#HY-15185B), Ferrostain-1 (#HY100579), Z-VAD-FMK (#HY16658B), FSEN1 (#HY-153629), BAI1 (#HY-103269), PEG300 (#HY-Y0873) and riboflavin (#HY-B0456) were obtained from MedChemExpress (Monmouth Junction, NJ). TWEEN 80 (#P1754) and DMSO (#D8418) from Sigma-Aldrich (Burlington, MA). DMF (#D119450), MK-2206 (#M129684) were purchased from Aladdin (Ontario, CA). Necrostatin 2 racemate (Nec-1s, #S8641) and RSL3 (#S8155) from Selleck (Houston, TX).

### Clinical samples

The tumor specimens and patient information (Table S1) were obtained from the First Affiliated Hospital of Zhejiang University, School of Medicine, Zhejiang University, Zhejiang Province, China. The 14 NSCLC specimens in the study were collected from 7 patients treated with immunotherapy and chemotherapy, 2 patients with targeted therapy, and 5 diagnosed (untreated) patients. All procedures were approved by the institutional review boards of the hospital and Zhejiang University (Approval No. IIT20230513B-R1), and conducted in accordance with the Belmont Report. The tumor specimens were processed according to the protocol previously described by Zhang et al. [12]. Specifically, the tissue digestion solution comprised 1 mg/mL collagenase I (#C8140, Solarbio, Beijing, China) and 1 mg/mL collagenase IV (#C8160, Solarbio), was used to digest tumor specimens for 1 hour.

### Animal experiments

Experimental mice were raised in a certified pathogen-free animal facility at Zhejiang University, in accordance with humane practice. All protocols were approved by the Ethics Review Committee of the Zhejiang University-University of Edinburgh (ZJE) Institute, Zhejiang University, Haining, Zhejiang Province, China (Approval No. ZJU20230534). No blinding was involved in animal experiments.

*NOD/ShiLtJGpt-Prkdc*^*em26Cd52*^*Il2rg*^*em26Cd22*^*/Gpt* (*NCG*) mice (four to six weeks old, male or female) were obtained from GemPharmatech (Nanjing, China) for xenograft assays. For the growth of SRCC, *NCG* mice were randomly distributed into 2 groups. A single-cell suspension of 1 × 10^6^ A549 or A549CR cells was mixed with Matrigel (#356231, Corning, NY) at a 1:1 ratio and injected subcutaneously into mice. The tumor mass was calculated at 2-day intervals, beginning at day 10 post injection, according to the formula [74]: length × width^2^ × π / 6 (mm^3^). For the administration of RO and cisplatin, a single-cell suspension of 2.5 × 10^6^ cisplatin-exposed A549 cells mixed with Matrigel at a 1:1 ratio was injected subcutaneously into mice. As outlined in Fig. S6C, grafted mice were randomly distributed into 4 groups and treated with diluent (10% DMSO, 40% PEG300, 5% Tween 80, 45% saline), 2.5 mg/kg cisplatin, 5 mg/kg RO, or a combination of 2.5 mg/kg cisplatin and 5 mg/kg RO at day 16 post injection. After 5 doses of cisplatin and/or 10 doses of RO, treated mice were sacrificed for tumors, followed by flow cytometric analysis with antibodies. SRCC were gated and analyzed in the mTer119^-^mCD45^-^mH-2Kd^-^7-AAD^-^ compartment.

Animals succumbing to non-tumor-related causes (e.g., pleural effusion-induced respiratory distress or severe inappetence) prior to the experimental endpoints were excluded from the final analysis.

### Cell culture

The human NSCLC cell lines, A549 (#SCSP-503), PC-9 (#SCSP-5085), and HCC827 (#SCSP-538), were obtained from the National Collection of Authenticated Cell Cultures, Shanghai, China. A549 cells were cultured in Ham’s F-12K medium (#21127022, Thermo, Waltham, MA), whereas PC-9 and HCC827 cells were maintained in RPMI 1640 medium (#R5886, Sigma-Aldrich). All media were supplemented with 10% heat-inactivated fetal bovine serum (FBS, #12A230, ExCell Bio, Shanghai, China). The cells were cultured at 37°C in a humidified incubator with 5% CO_2_. The transient transfection and lentiviral infection were conducted according to the methods in Supplementary Materials and Methods.

The authentication of the cells was conducted via PCR-based short tandem repeat profiling (see Supplementary Methods), and mycoplasma contamination was routinely inspected via PCR. All the PCR primer pairs are listed in Table S8.

### Cisplatin treatment

IC_50_ values for cisplatin were determined and cisplatin-resistant cells were generated according to the methods in the Supplementary Materials and Methods. To investigate the correlation between autofluorescence and riboflavin (and/or its derivatives), A549CR cells were enriched through 4 cycles of cisplatin treatment in a riboflavin-free medium (#MBS652982, MyBioSource, San Diego, CA), which was supplemented with 4 additional components including 10 mmol/L nicotinamide, 10 mmol/L folic acid (#F8758, Sigma), 10 mmol/L thiamin hydrochloride (#T1270), and 10 mmol/L pyridoxine hydrochloride (#P6280, Fig. S1H). After 28 days of cisplatin treatment, the autofluorescence in the enriched A549CR cells was measured using flow cytometric analysis.

### Flow cytometric analysis and sorting

The cultured cells were dissociated using TrypLE Express (#12604021, Thermo) and filtered through 40 μm cell strainers. The dissociated single cells were resuspended in HBSS+ buffer (1 × HBSS, 10 mmol/L HEPES, 2% bovine serum, 1% penicillin/streptomycin) and incubated with allophycocyanin (APC) or R-phycoerythrin (R-PE)-conjugated anti-NOTCH1 antibodies for 15 min at 4 °C. After a 10-minute incubation with 0.005 mg/mL 7-AAD (#A606804, Sangon, Shanghai, China), the stained single cells were subjected to flow cytometric analysis. For specific experiments using less than 5,000 cisplatin-resistant cells, they were mixed with 10^6^ murine Ba/F3 carrier cells to minimize cell loss during pipetting and centrifugation. Human cells were selected using the mCD45^-^mH-2kd^-^ gate in flow cytometric analysis.

Protein-flow or phopsho-flow analysis with antibodies (targeting Ki-67, SLC52A2, GPX4, FSP1, ACSL4, Flag, p-AKT, p-MLKL and activated BAX) was conducted, using the FIX & PERM Cell Permeation Kit (#GAS004, Thermo) and Zombie Aqua Fixable Viability Kit (#423101, Biolegend, San Diego, CA), in accordance with the manufacturers’ instructions. Annexin V assays were conducted using the Annexin V-PE/Cyanine 7/7-AAD Apoptosis Kit (#E-CK-A228, Elabscience, Houston, TX) or the Annexin V-APC/Cyanine 7/7-AAD Apoptosis Kit (#E-CK-A230, Elabscience). For EdU proliferation assays, cells were incubated with EdU from the BeyoClick™ EdU Cell Proliferation Kit with Alexa Fluor 594 (#C0078L, Beyotime, Shanghai, China) for 24 hours, fixed and permeabilized using the FIX & PERM Cell Permeation Kit and Zombie Aqua Fixable Viability Kit, and labeled by the Click additive cocktail in the BeyoClick™ EdU Cell Proliferation Kit with Alexa Fluor 594.

ROS assays were conducted using the CellROX™ Deep Red Flow Analysis Kit (#C10491, Thermo), in accordance with the manufacturers’ instructions. Ferrous ion was detected using a FerroOrange dye (#F374, Dojindo, Mashiki, Japan), in accordance with the manufacturers’ instructions. Lipid peroxidation was assessed using the probe BODIPY™ 581/591 C11 (#D3861, Thermo), according to the manufacturers’ instructions. Caspase-3 activity was detected using the NucView^®^ 488 Caspase-3 Assay Kit for Live Cells (#30029-T, Biotium, Fremont, CA), following the manufacturer’s instructions.

Flow cytometric analysis was performed on a Cytek Aurora (Cytek, Fremont, CA) or an ACEA Novocyte cytometer (Agilent, Santa Clara, CA), and the data were subsequently analyzed using FlowJo (BD Bioscience, Franklin Lakes, NJ) or NovoExpress. The MFI is calculated as follows [75]:

$${MFI}=\frac{\sum ({Fluorescence\; Intensit}{ies\; of\; individual\; cells})}{{\rm{Total\; number\; of\; cells\; analyzed}}}$$

The riboflavin^+^NOTCH1^+^ or riboflavin^-^NOTCH1^−^ cells were sorted on a BD Influx sorter (BD Bioscience) with a 100 µm nozzle and a 1.0 drop single mode, which is the highest purity mode available. The antibodies used in flow cytometry are listed in Table S9.

### Fluorescent and confocal imaging

To detect riboflavin fluorescence, cells were cultured in 6-well plates and observed under a Nikon ECLIPES Ts2 fluorescent microscope (Tokyo, Japan). To evaluate mitochondria integrity, cells were cultivated in confocal dishes (#801002, NEST, Wuxi, China) for 24 hours and subsequently exposed to 20 nmol/L MitoTracker™ Red CMXRos (#M7512, Thermo) for 20 min, in accordance with the manufacturer’s instructions. The stained mitochondria were observed on the LSM880 live imaging system with a 63× oil immersion lens (Zeiss, Oberkochen, Germany) using the ZEN 3.4 software for data acquisition and analysis.

To analyze the colocalization of lysosomes and Fe^2+^, cells were seeded in confocal dishes and cultured for 24 hours. The attached cells were exposed to 1 μmol/L SiR-lysosome probe (#SC012, Genevivo, San Marino, CA) for 1 hour. After the SiR-lysosome-containing medium was replaced with fresh medium containing 1 µmol/L FerroOrange probe, the cells were observed immediately on the LSM880 live imaging system equipped with a 63× oil immersion lens and the ArrayScan mode. The confocal images were processed using ImageJ with region of interest manager and plot profile, and displayed using Origin (OriginLab, Northampton, MA).

In order to determine the growth of colonies cultured in a cisplatin-free environment, resistant cells were first seeded into a 6-well plate, and monitored and scanned on the ImageXpress^®^ Micro Confocal High-Content Imaging system (Molecular Devices, San Jose, CA) with MetaXpress software. Images were captured with a 24 × 24 grid configuration.

### Molecular cloning and analysis

The lentiviral plasmids with *NOTCH1* shRNAs were constructed by GenePharma (Shanghai, China). The lentiviral plasmids with Flag-tagged NICD1 (4536–6944 bp) or mNICD1 with the deletion of the RBPJ-binding RAM domain (4608–6944 bp) were cloned into the vector pLVX-M-puro or pLV3-EF1a-MCS-PGK-copGFP-Puro. RT-PCR was conducted according to the detailed method in Supplementary Materials and Methods. Primer sequences are listed in Table S8. Western blotting analysis was conducted according to the detailed method in Supplementary Materials and Methods. The primary and secondary antibodies employed are listed in Table S9.

### Bulk RNA-seq analysis

A single-cell suspension was washed with 1 × PBS and lysed in TRI reagent (#T9424, Sigma). The RNA lysates were subjected to bulk RNA-seq at GENEWIZ (Leipzig, Germany). Briefly, poly(A)^+^ mRNA were extracted for library preparation using the VAHTS mRNA Capture Beads and the VAHTS Universal V8 RNA-seq Library Prep Kit for Illumina (#N401, #NR605-02, Vazyme). The cDNA libraries were sequenced on an *Illumina HiSeq*, *Illumina NovaSeq*, or *MGI2000* sequencer with the 2 × 150 bp paired-end configuration. The raw sequencing data were analyzed on a locally installed Galaxy instance (version 20.0965). RNA-seq analysis including differential expression analysis, GO analysis, and GSEA is detailed in Supplementary Materials and Methods. The gene sets used in GSEA are listed in Table S2.

### scRNA-seq analysis

The scRNA-seq dataset GSE131907 was obtained from the GEO database and analyzed using the *Seurat* R package (version 5.1.0). Briefly, the cells with fewer than 200 features (genes) and more than 5% mitochondrial genes were excluded from subsequent analysis. Raw expression counts were normalized with *NormalizeData()* and scaled with *ScaleData()* for a subset of highly variable features. Cell populations were clustered using PCA and UMAP and visualized on 2-dimensional plots. The clusters of epithelial cells, fibroblasts, and B cells were then selected for CNV analysis using *InferCNV* (version 1.21.0). Four tumor clusters with CNV scores greater than 1.009 (Table S6) were combined for the detection of the rare *SLC52A2*^*+*^*NOTCH1*^*+*^ population.

To cluster rare SRCC on UMAP plots, a modified PCA clustering analysis with input gains (or pre-amplified expression) for the marker genes *SLC52A2* and *NOTCH1* was implemented in Seurat’s analytic pipeline (Fig. S4G). The 5-fold increase in the expression of the SRCC markers enabled clustering of the *SLC52A2*^*+*^*NOTCH1*^*+*^ SRCC population from CNV^+^ epithelial cancer cells. DEG between SRCC and non-SRCC were determined using the Seurat *FindMarkers* function with *DESeq2* tests and subjected to GSEA using *GSEABase* and *fgsea*. The GSEA gene sets employed are listed in Table S2.

### Statistical analysis

All experiments involving statistical comparison were independently repeated 3-4 times. Few experiments in figures and supplementary figures were independently repeated less than 3 times. Detailed information on the numbers of independent experiments or sample sizes is indicated in figure legends. Student’s two-tailed, unpaired *t*-tests or paired *t*-tests were employed for comparisons between 2 groups only when the data met the key assumptions of parametric tests: a normal distribution, homogeneity of variance between groups, and independence. One way ANOVA with Tukey’s test is utilized for comparisons between two groups in the experiments with 3 or more groups. Individual data points within an experimental group are displayed on scatter plots with/without bars to visualize the full distribution, and their variation is indicated by SD in all figures. A statistically significant difference was deemed when *P* < 0.05.

In xenograft tumor studies, the number of animals per group was determined based on the statistical power of comparable xenograft studies. Specifically, 3 animals were selected for experiments examining SRCC growth, and 5-8 animals were chosen for experiments assessing the effect of RO and cisplatin on SRCC. *NCG* mice were randomly selected as recipients for tumor engraftment. Following successful engraftment, mice were randomly allocated into different treatment groups. For molecular and cellular assays, randomization was not required due to the controlled experimental conditions. No blinding procedures were employed in this study.

The default statistical models were used for bulk RNA-seq analysis and scRNA-seq analysis. Transcriptional comparison of individual genes in RNA-seq data employed Wald tests, which was corrected with the Benjamini-Hochberg procedure. For non-systems experiments, statistical analysis was conducted using the software GraphPad Prism 8.0.

## Supplementary information

Supplementary Materials and Methods

Supplemental Figures

Supplementary Table 1

Supplementary Table 2

Supplementary Table 3

Supplementary Table 4

Supplementary Table 5

Supplementary Table 6

Supplementary Table 7

Supplementary Table 8

Supplementary Table 9

Original Western blot data

## Data Availability

The sequencing data have been deposited in the National Genomics Data Center database (Beijing, China) with the accession number HRA007891 (https://ngdc.cncb.ac.cn/gsa-human/submit/hra/subHRA011407/finishedOverview). The datasets GSE131907 and phs000178 are accessible via the GEO database (https://www.ncbi.nlm.nih.gov/geo/query/acc.cgi?acc=GSE131907) and the TCGA database (https://www.ncbi.nlm.nih.gov/projects/gap/cgi-bin/study.cgi?study_id=phs000178.v11.p8).

## Code availability

The code utilized to cluster the SRCC population in scRNA-seq data is available via the GitHub link (https://github.com/wei-R45/SRCC).
