# Supplementary material for: Non-canonical NOTCH1 signaling regulates ferroptosis vulnerability in dormant lung cancer cells with stable resistance
Source: Cell Death Dis. 2025 Dec 26;17(1):1. doi: 10.1038/s41419-025-08355-9 (PMC12780219; doi:10.1038/s41419-025-08355-9)
Supplement: Supplementary file 1 — Supplementary Materials and Methods [file 41419_2025_8355_MOESM1_ESM.pdf]

Non-canonical NOTCH Signaling Regulates Ferroptosis Vulnerability in Stable Resistant Lung Cancer Cells, Huang H *et. al.*

Supplementary Materials and Methods

Cisplatin resistance assays

In order to determine the IC_{50} values of cisplatin, 10 000 cells were seeded into individual wells of a 96-well plate and exposed to varying concentrations (0.1, 1, 3, 10, 30, 100 μmol/L) of cisplatin for 72 hours. The treated cells were harvested for Cell Counting Kit-8 (CCK-8) assays using the Cell Counting Kit-8 kit (#HY-K0301, MedChemExpress) following the manufacturer's instructions. The IC_{50} for cisplatin in individual cell lines was determined using a nonlinear regression curve in GraphPad.

To generate cisplatin-resistant cells, A549, HCC827, and PC-9 cell lines were cultured in medium containing 20 μM, 10 μM, and 3.5 μM cisplatin (corresponding to their respective IC_{50} values) for three days. Subsequently, the cells were transferred to cisplatin-free medium and maintained for an additional three days. Following the repeated administration of cisplatin over 3-4 rounds, the surviving cells were designated as cisplatin-resistant A549CR and HCC827CR cells.

Plasmids

The lentiviral plasmids with *NOTCH1* shRNAs were designed and purchased from GenePharma (Shanghai, China). The targeting sequences in these constructs: 5'-GAGTGTGAATCCAACCCTTGT-3' and 5'-GGAGCATGTGTAACATCAACA-3', respectively. The lentiviral plasmids with Flag-tagged NICD1 (4536 - 6944 bp) or a mutant NICD1 with the deletion of the RBPJ-binding RAM domain (4608 - 6944 bp) were cloned into between the BamH I and EcoR I sites of the vector pLVX-M-puro or pLV3-EF1a-MCS-PGK-copGFP-Puro.

Transient transfection and stable infection

To generate stable cell lines A549-*shNOTCH1* and HCC827-*shNOTCH1*, lentivirus carrying sh*NOTCH1*-1 and sh*NOTCH1*-2 were generated and packaged in 293FT cells, using the packaging plasmids pCMV-VSV-G and pCMV-dR8.91. The packaged lentivirus then infected A549 and HCC827 cells and mCherry+ clones were sorted for validation by Western blot analysis and subsequent experiments.

For transient transfection, A549-*shNOTCH1* stable cells were transfected with the validated plasmids using the PEI transfection reagent (#23966-1, Polysciences, Warrington, PA). After transfection, cells were harvested for protein-flow or phospho-flow analysis.

Western blot analysis

The cultured cells were lysed using 1× RIPA lysis buffer (150 mmol/L sodium chloride, 50 mmol/L Tris [pH 8.0], 1% Triton X-100, 1% sodium deoxycholate, and 0.1% SDS) on ice. The protein concentrations were determined using the bicinchoninic acid assays. The protein lysates were resolved on SDS-PAGE and transferred to Immobilon®-P membranes (#ISEQ00010, Merck Millipore, Burlington, MA), which were stained with primary and secondary antibodies. The stained proteins were visualized using the E170 StarSignal Plus Chemiluminescent Assay Kit (#E170, Genstar, Beijing, China). ATP1A1 or GAPDH was used as a loading control.

RT-PCR

RNA were extracted from cells, converted into cDNA using the FlysisAmp Cells-to-cDNA Kit (CL111-01, Vazyme, Nanjing, China) and subjected to quantitative PCR using the Taq Pro Universal SYBR qPCR Master Mix (Q712-02, Vazyme) on a CFX96 Touch Real-Time PCR Detection System (Bio-Rad, Hercules, CA). Primer sequences are listed in Supplementary Table 8. Data analysis was performed using the Livak method [1].

Bulk RNA-seq analysis

A single-cell suspension was washed with 1 × PBS and lysed in TRI reagent (#T9424, Sigma-Aldrich). The RNA lysate was subjected to bulk RNA-seq at

GENEWIZ (Leipzig, Germany). Briefly, poly(A)$^+$ mRNA was extracted for library preparation using the VAHTS mRNA Capture Beads and the VAHTS Universal V8 RNA-seq Library Prep Kit for Illumina (#N401, #NR605-02, Vazyme). The cDNA libraries were sequenced on an *Illumina HiSeq*, *Illumina NovaSeq*, or *MGI2000* sequencer with the 2 × 150 bp paired-end configuration. The raw sequencing data were collected.

Fastq data were analyzed on a locally installed Galaxy instance (version 20.0965) [2]. Sequencing reads were processed with *fastp* (parameters: --detect_adapter_for_pe -f 15 -F 15 -q 20 -u 10 -n 1) and aligned to the human reference genome GRCh38 using the aligner *RNA STAR* with the default parameters [3]. The gene expression levels were then estimated using *featureCounts* [4]. The DEG were determined using *DESeq2* analysis [5], with a significance threshold of $p < 0.05$, and visualized using *heatmap2* and *Volcano Plot*.

The GO analysis was conducted on the DAVID server [6,7] (https://david.ncifcrf.gov/tools.jsp). The statistical framework relies on a hypergeometric distribution model to assess the overrepresentation of GO terms within the input gene list. This model assumes random sampling of genes from the entire genome and calculates the probability of observing at least k genes associated with a specific GO term in a list of n genes, given that the term is annotated to M genes in the genome. The enrichment significance is computed as:

$$P(X \geq K) = \sum_{i=k}^{min(n,M)} \frac{\binom{M}{i}\binom{N-M}{n-i}}{\binom{N}{n}},$$

where N represents the total number of genes in the genome. To address multiple testing across thousands of GO terms, the Benjamini-Hochberg method was applied to control the false discovery rate (FDR) at a threshold of 0.05.

GSEA was conducted using the Broad Institute *GSEA* software, with weighted enrichment statistic to prioritize gene sets with coordinated expression changes concentrated at the extremes of the ranked gene list. Genes were ranked by their Signal2Noise metric, calculated as the ratio of the difference in class means to the sum of standard deviations, and sorted in descending order. Gene sets containing fewer than 15 or more than 2000 genes were excluded to mitigate noise. The enrichment score (ES) for each gene set was computed using a weighted Kolmogorov-Smirnov-like running sum statistic. To assess significance, phenotype labels were permuted 1,000 times to generate a null distribution of enrichment scores. The GSEA gene sets are listed in Table S7.

scRNA-seq analysis

The scRNA-seq dataset GSE131907 was obtained from the GEO database and analyzed using the *Seurat* R package (version 5.1.0) [8]. Briefly, the cells with fewer than 200 features (genes) and more than 5% mitochondrial genes were excluded from subsequent analysis. Raw expression counts were normalized with *NormalizeData()* and scaled with *ScaleData()* for a subset of highly variable

features. Cell populations were clustered using PCA and UMAP and visualized on 2-dimensional plots. The clusters of epithelial cells, fibroblasts, and B cells were then selected for CNV analysis using *InferCNV* (version 1.21.0), which is described in detail below. The 4 tumor clusters with a CNV score greater than 1.009 (Table S6) were combined for the detection of the rare *SLC52A2*$^{+}$*NOTCH1*$^{+}$ population.

CNV scores were derived to quantify chromosomal aberrations by analyzing normalized gene expression deviations from a reference baseline (e.g., B cells). To reduce noise and model chromosomal spatial continuity, a hidden Markov model (HMM) segmented the genome into contiguous regions with shared expression states (normal, amplification, deletion), leveraging transition probabilities to account for adjacent genomic dependencies. For each segment, the CNV score was computed as the mean absolute deviation of normalized expression values:

$$\mathrm{CNV\ score} = \frac{1}{N}\sum_{i=1}^{N}|E_i - \mu_{\mathrm{reference}}|,$$

where E_i represents the normalized expression of gene i, $\mu_{\mathrm{reference}}$ is the reference population's mean expression, and N is the number of genes in the segment. Amplifications (scores > threshold) and deletions (scores < threshold) were identified by comparing segment scores to empirically defined thresholds.

InferCNV (version 1.21.0) was employed to identify CNVs in single-cell transcriptomic data by integrating hierarchical clustering with a HMM. The

analysis begins with hierarchical clustering to group cells based on expression similarity, providing a preliminary stratification of the dataset. Subsequently, a HMM is applied to infer CNV patterns across genomic regions. The HMM models chromosomal continuity by defining discrete states (e.g., normal, amplification, deletion) and transition probabilities between adjacent genomic loci. All parameters were set as default.

To cluster rare cell populations on UMAP plots, a modified PCA clustering analysis with input gains (or pre-amplified expression) for the marker genes *SLC52A2* and *NOTCH1* was implemented in Seurat's analytic pipeline (Fig. S4G). The 5-fold increase in expression of the SRCC markers enabled clustering of the *SLC52A2*$^{+}$*NOTCH1*$^{+}$ SRCC population from CNV^{+} epithelial cancer cells. DEG between SRCC and non-SRCC were determined using the Seurat *FindMarkers* function with *DESeq2* tests and subjected to GSEA using *GSEABase* and *fgsea* [9]. The GSEA gene sets employed are listed in Table S2.

STR analysis

The genomic DNA of PC-9, A549, HCC827, A549CR and HCC827CR were extracted for PCR amplification with STR primers (their sequences were listed in the Table S5). The PCR products were combined and sent to the GENEWIZ for next-generation sequencing. The STR analysis was performed on the Galaxy sever [2], using a customized pipeline.

References

1 Livak KJ, Schmittgen TD. Analysis of Relative Gene Expression Data Using Real-Time Quantitative PCR and the 2−ΔΔCT Method. Methods. 2001;25:402–8.

2 The Galaxy Community, Afgan E, Nekrutenko A, Grüning BA, Blankenberg D, Goecks J, et al. The Galaxy platform for accessible, reproducible and collaborative biomedical analyses: 2022 update. Nucleic Acids Res. 2022;50:W345–51.

3 Dobin A, Davis CA, Schlesinger F, Drenkow J, Zaleski C, Jha S, et al. STAR: ultrafast universal RNA-seq aligner. Bioinformatics. 2013;29:15–21.

4 Liao Y, Smyth GK, Shi W. featureCounts: an efficient general purpose program for assigning sequence reads to genomic features. Bioinformatics. 2014;30:923–30.

5 Love MI, Huber W, Anders S. Moderated estimation of fold change and dispersion for RNA-seq data with DESeq2. Genome Biol. 2014;15:550.

6 Huang DW, Sherman BT, Lempicki RA. Systematic and integrative analysis of large gene lists using DAVID bioinformatics resources. Nat Protoc. 2009;4:44–57.

7 Sherman BT, Hao M, Qiu J, Jiao X, Baseler MW, Lane HC, et al. DAVID: a web server for functional enrichment analysis and functional annotation of gene lists (2021 update). Nucleic Acids Res. 2022;50:W216–21.

8 Hao Y, Stuart T, Kowalski MH, Choudhary S, Hoffman P, Hartman A, et al. Dictionary learning for integrative, multimodal and scalable single-cell analysis. Nat Biotechnol. 2024;42:293–304.

9 Korotkevich G, Sukhov V, Budin N, Shpak B, Artyomov MN, Sergushichev A. Fast gene set enrichment analysis. preprint at https://www.biorxiv.org/content/10.1101/060012v3 (2016).
