# Supplementary material for: Non-canonical NOTCH1 signaling regulates ferroptosis vulnerability in dormant lung cancer cells with stable resistance
Source: Cell Death Dis. 2025 Dec 26;17(1):1. doi: 10.1038/s41419-025-08355-9 (PMC12780219; doi:10.1038/s41419-025-08355-9)
Supplement: Supplementary file 2 — Supplemental Figures [file 41419_2025_8355_MOESM2_ESM.pdf]

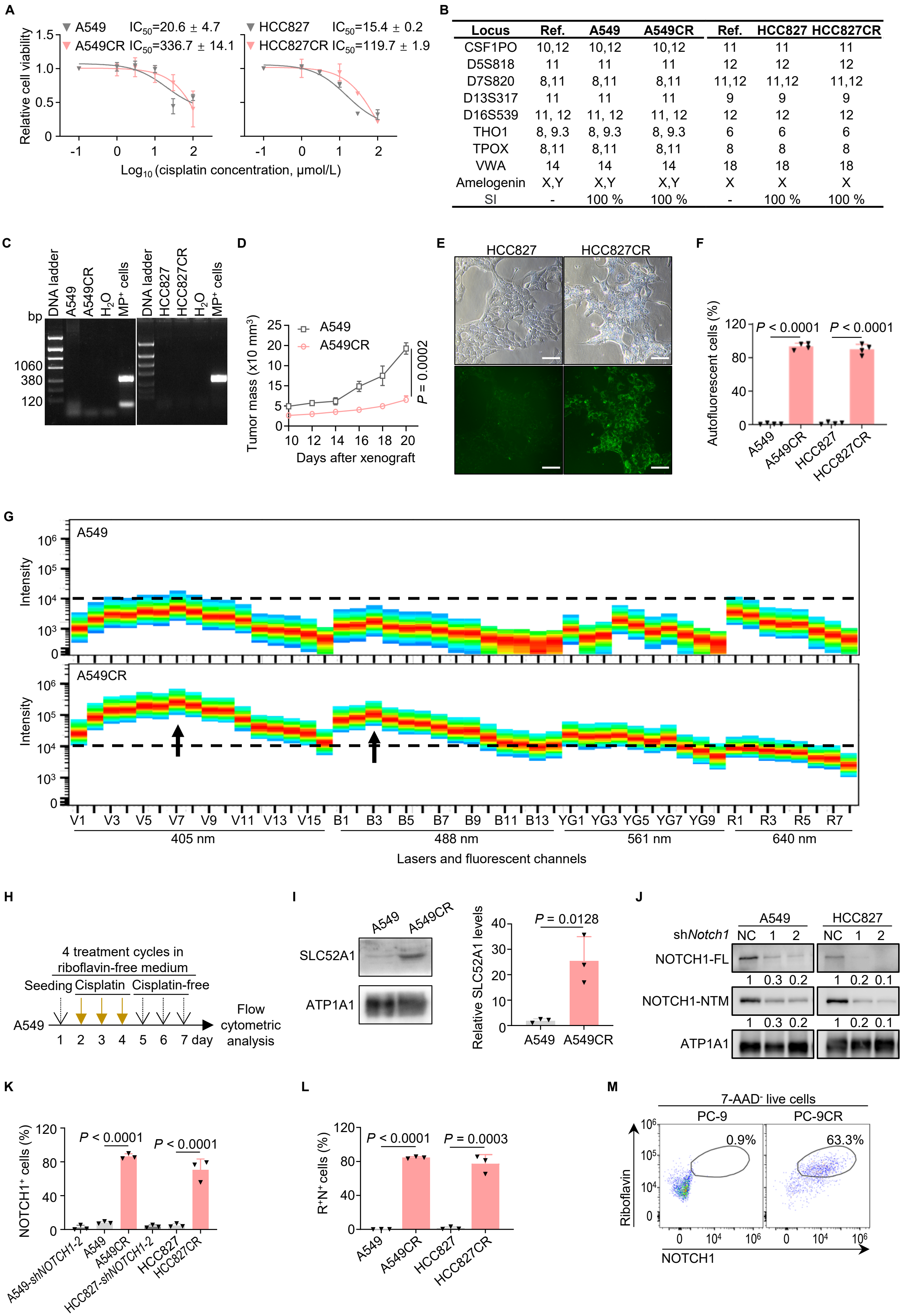

Locus	Ref.	A549	A549CR	Ref.	HCC827	HCC827CR
CSF1PO	10,12	10,12	10,12	11	11	11
D5S818	11	11	11	12	12	12
D7S820	8,11	8,11	8,11	11,12	11,12	11,12
D13S317	11	11	11	9	9	9
D16S539	11, 12	11, 12	11, 12	12	12	12
THO1	8, 9.3	8, 9.3	8, 9.3	6	6	6
TPOX	8,11	8,11	8,11	8	8	8
VWA	14	14	14	18	18	18
Amelogenin	X,Y	X,Y	X,Y	X	X	X
SI	-	100 %	100 %	-	100 %	100 %

Figure S1: The riboflavin$^+$NOTCH1$^+$ population is identified in untreated and cisplatin-treated lung cancer cell lines. A Response curves of A549, A549CR, HCC827 and HCC827CR to cisplatin were determined using CCK-8 assays and used to calculate IC_{50} (n = 2 independent experiments). **B** STR profiling of parental and surviving cells after 4 cycles of cisplatin treatment. The similarity index (SI) values in the table indicate the similarity between cisplatin-treated cells and their parental counterparts. The reference STR information was obtained from the Cellosaurus database. **C** Evaluation of mycoplasma contamination by PCR for A549CR and HCC827CR cells. MP, mycoplasma positive. **D** Growth of xenograft tumors in the *NCG* mice injected subcutaneously with 1×10^6 A549 and A549CR cells was monitored at 2-day intervals after injection (n = 3 mice per group). **E** Representative images of autofluorescence in HCC827 and HCC827CR cells. Scale bar, 100 μm. **F** Flow cytometric quantification of autofluorescent cells (n = 4 independent experiments). See also Fig. 1D. **G** Spectrum of autofluorescence in A549 and A549CR cells was detected on a Cytek Aurora 4-laser cytometer. The arrows indicate the emission peaks of autofluorescence excited by 405 nm and 488 nm lasers, respectively. **H** Schematic diagram for assessing riboflavin fluorescence in A549CR cells, which were enriched by cisplatin treatment in a riboflavin-free medium for 28 days. **I** Western blot analysis (left panel) and quantification (right panel) of the SLC52A1 protein in A549CR cells (n = 3 independent experiments). **J** Western blot analysis of the NOTCH1 protein in A549 and HCC827 cells, in which *NOTCH1* was knocked down using 2 different shRNA. ATP1A1 was used as a loading control (n = 1 independent experiment). FL, full length; NTM, NOTCH transmembrane and intracellular domains. **K** Quantification of the NOTCH1$^+$ fractions in A549/A549CR and HCC827/HCC827CR cells, as well as sh*NOTCH1*-bearing A549 and HCC827 cells. See also Fig. 1F. **L** Flow cytometric quantification of the riboflavin$^+$NOTCH1$^+$ (or R^+N^+) fractions in A549/A549CR and HCC827/HCC827CR cells (n = 3 independent experiments). See also Fig. 1G. **M** Flow cytometric analysis of the riboflavin$^+$NOTCH1$^+$ population in PC-9 and PC-9CR cells (n = 1 independent experiment). Error bars, mean $\pm$ SD; *P* values were calculated by Student's unpaired *t*-tests (**F**, **I**, **L**) or one way ANOVA with Tukey's tests (**K**).

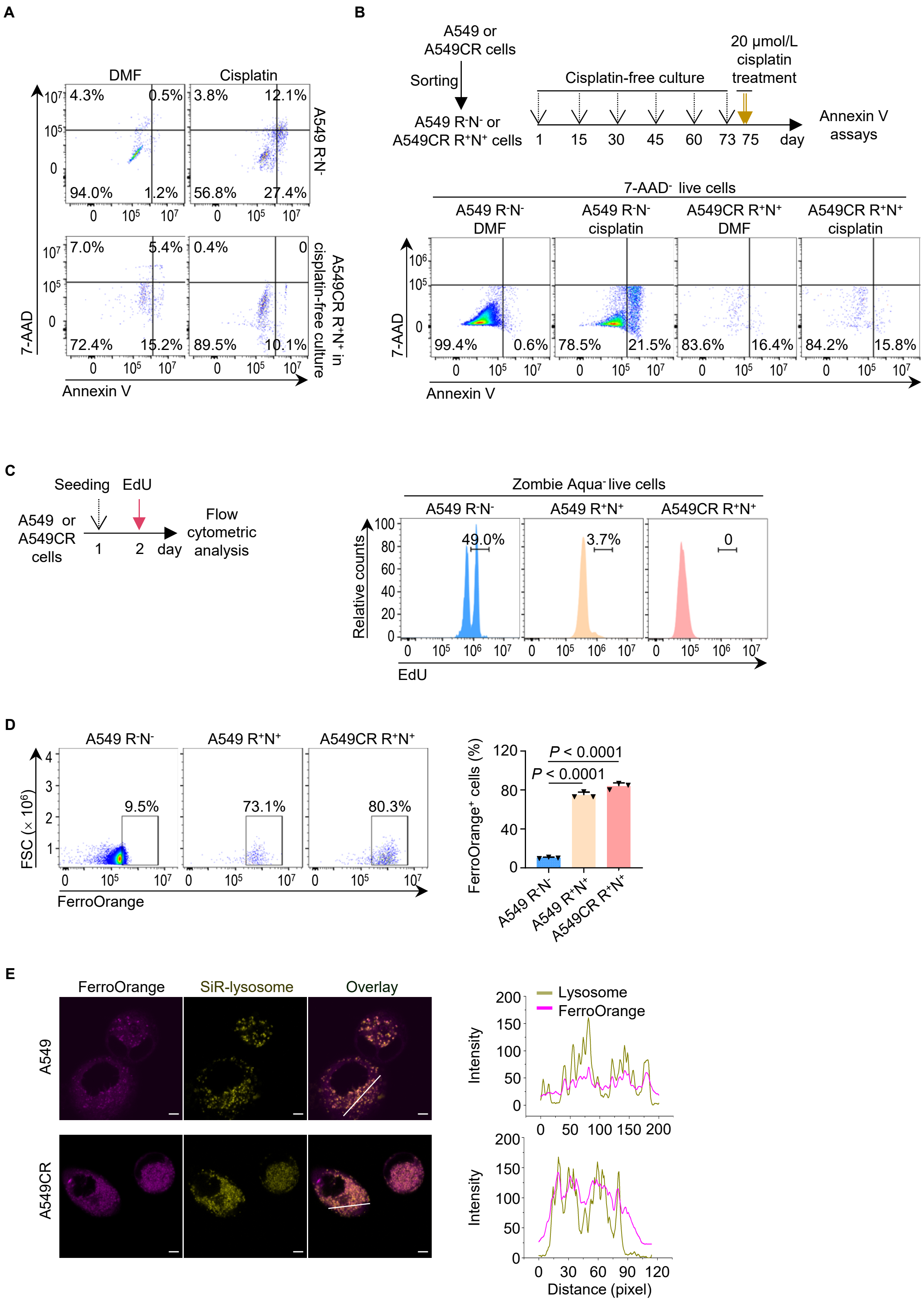
A
DMF
Cisplatin
4.3%
0.5%
3.8%
12.1%
94.0%
1.2%
56.8%
27.4%
A549 R-N-
7.0%
5.4%
0.4%
0
72.4%
15.2%
89.5%
10.1%
A549CR R+N+ in cisplatin-free culture
7-AAD
Annexin V
B
A549 or A549CR cells
Sorting
A549 R-N- or A549CR R+N+ cells
Cisplatin-free culture
20 μmol/L cisplatin treatment
1
15
30
45
60
73
75
day
Annexin V assays
7-AAD- live cells
A549 R-N- DMF
A549 R-N- cisplatin
A549CR R+N+ DMF
A549CR R+N+ cisplatin
99.4%
0.6%
78.5%
21.5%
83.6%
16.4%
84.2%
15.8%
7-AAD
Annexin V
C
Seeding
EdU
A549 or A549CR cells
1
2
day
Flow cytometric analysis
Zombie Aqua- live cells
A549 R-N-
A549 R+N+
A549CR R+N+
49.0%
3.7%
0
Relative counts
EdU
D
A549 R-N-
A549 R+N+
A549CR R+N+
9.5%
73.1%
80.3%
FSC (× 10^6)
FerroOrange
FerroOrange+ cells (%)
P < 0.0001
P < 0.0001
A549 R-N-
A549 R+N+
A549CR R+N+
E
FerroOrange
SiR-lysosome
Overlay
A549
A549CR
Lysosome
FerroOrange
Intensity
Distance (pixel)

Figure S2: Characterization of the cisplatin-naïve and cisplatin-exposed riboflavin^{+}NOTCH1^{+} populations. A Representative Annexin V assay for the irreversible resistance of sorted riboflavin^{+}NOTCH1^{+} cells after 8 days of cisplatin-free culture (n = 3 independent experiments). The experimental design is shown in Fig. 2C and the results are summarized in Fig. 2D. **B** Assessment of stable resistance of sorted riboflavin^{+}NOTCH1^{+} A549CR cells to 2-day cisplatin treatment after 73 days of cisplatin-free culture (n = 1 independent experiment). The top panel is an experimental plot and the bottom panel is an Annexin V assay. **C** EdU proliferation assays for cisplatin-naïve and cisplatin-exposed riboflavin^{+}NOTCH1^{+} populations gated from A549 and A549CR cells, respectively (n = 1 independent experiment). The left panel is an experimental diagram and the right panel is flow cytometric analysis. **D** Representative flow cytometric analysis (left panel) and quantification (right panel) with FerroOrange (Fe^{2+} indicator) in cisplatin-naïve and -exposed riboflavin^{+}NOTCH1^{+} A549 cells (n = 3 independent experiments). **E** Colocalization analysis of Fe^{2+} and lysosome in A549 and A549CR cells, using a Fe^{2+} probe (FerroOrange, in rose red) and lysosome dye (SiR-lysosome, in yellow). The left panel displays representative confocal images, while the right panel presents the intensity profiles of the FerroOrange and lysosome fluorescence along a white line across cells (n = 1 independent experiment). Scale bar, 5 μm. R^{-}N^{-}, riboflavin^{-}NOTCH1^{-}; R^{+}N^{+}, riboflavin^{+}NOTCH1^{+}; DMF, N, N-dimethylformamide (cisplatin diluent). Error bars, mean ± SD; *P* values were calculated by one way ANOVA with Tukey's tests (**D**).

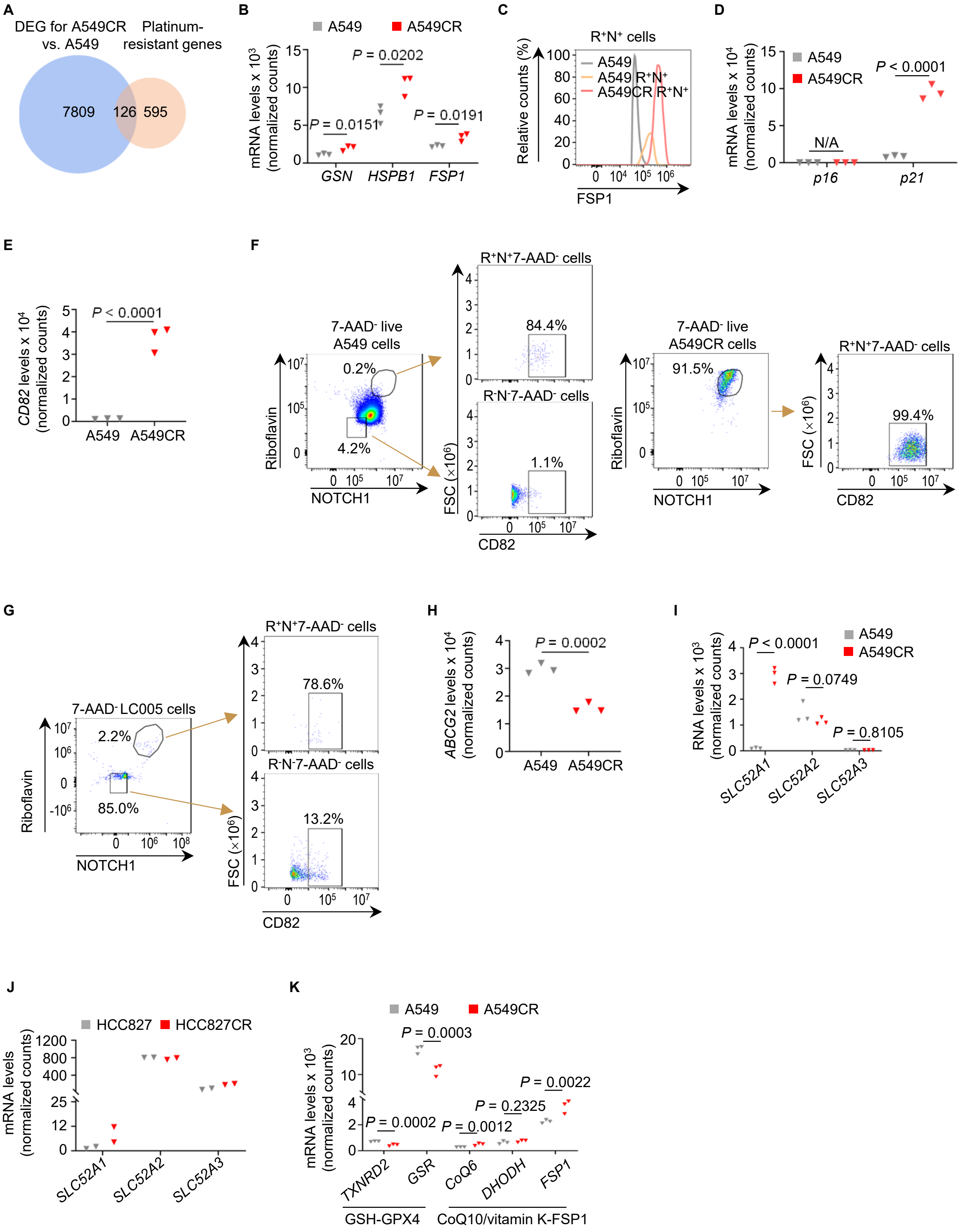

A
DEG for A549CR vs. A549
Platinum-resistant genes
7809
126
595
B
A549
A549CR
mRNA levels x 10^3 (normalized counts)
P = 0.0202
P = 0.0151
P = 0.0191
GSN
HSPB1
FSP1
C
R+N+ cells
Relative counts (%)
A549
A549 R+N+
A549CR R+N+
FSP1
D
mRNA levels x 10^4 (normalized counts)
A549
A549CR
P < 0.0001
N/A
p16
p21
E
CD82 levels x 10^4 (normalized counts)
P < 0.0001
A549
A549CR
F
7-AAD- live A549 cells
0.2%
4.2%
Riboflavin
NOTCH1
R+N+7-AAD- cells
84.4%
R-N-7-AAD- cells
1.1%
FSC (×10^6)
CD82
7-AAD- live A549CR cells
91.5%
R+N+7-AAD- cells
99.4%
G
7-AAD- LC005 cells
2.2%
85.0%
Riboflavin
NOTCH1
R+N+7-AAD- cells
78.6%
R-N-7-AAD- cells
13.2%
FSC (×10^6)
CD82
H
ABCG2 levels x 10^4 (normalized counts)
P = 0.0002
A549
A549CR
I
RNA levels x 10^3 (normalized counts)
A549
A549CR
P < 0.0001
P = 0.0749
P = 0.8105
SLC52A1
SLC52A2
SLC52A3
J
HCC827
HCC827CR
mRNA levels (normalized counts)
SLC52A1
SLC52A2
SLC52A3
K
A549
A549CR
mRNA levels x 10^3 (normalized counts)
P = 0.0003
P = 0.0002
P = 0.0012
P = 0.2325
P = 0.0022
TXNRD2
GSR
CoQ6
DHODH
FSP1
GSH-GPX4
CoQ10/vitamin K-FSP1

Figure S3: The molecular programs of platinum resistance, cell dormancy, and antioxidant control are associated with SRCC. A Venn diagram illustrating 126 platinum-resistant genes in the DEG identified by a transcriptomic comparison between A549CR and A549 cells. **B** Transcriptional comparison of the anti-apoptotic and anti-ferroptotic genes *GSN*, *HSPB1* and *FSP1* between A549 and A549CR cells, using bulk RNA-seq analysis with DEseq2 (n = 3 independent experiments). **C** Protein-flow analysis with an anti-FSP1 antibody was used to detect FSP1 expression in cisplatin-naïve and -exposed riboflavin^{+}NOTCH1^{+} cells (n = 3 independent experiment). **D,E** Transcriptional comparison of the senescence regulatory genes *p16* and *p21* (**D**), and the dormancy marker *CD82* (**E**) between A549 and A549CR cells, using bulk RNA-seq analysis with DEseq2 (n = 3 independent experiments). **F** Representative flow cytometric analysis of CD82 expression in the riboflavin^{-}NOTCH1^{-}7-AAD^{-} and riboflavin^{+}NOTCH1^{+}7-AAD^{-} populations derived from A549 and A549CR cells. See the summarized results (n = 3 independent experiments) in Fig. 3K. **G** Representative flow cytometric analysis of CD82 expression in lung cancer specimens. See the summarized results (n = 4 patients) in Fig. 3L. **H** Transcriptional comparison of the transporter gene *ABCG2* between A549 and A549CR cells, using bulk RNA-seq analysis with DEseq2 (n = 3 independent experiments). **I** Transcriptional comparison of *SLC52A1*, *SLC52A2* and *SLC52A3* between A549CR and A549 cells, using RNA-seq analysis with DEseq2. **J** Transcriptional comparison of the riboflavin transporter genes *SLC52A1*, *SLC52A2* and *SLC52A3* between HCC827 and HCC827CR cells, using bulk RNA-seq analysis (n = 2 independent experiments). **K** Transcriptional comparison of the antioxidant genes *TXNRD2*, *GSR*, *CoQ6*, *DHODH* and *FSP1* between A549 and A549CR cells, using bulk RNA-seq analysis with DEseq2 (n = 3 independent experiments). R^{-}N^{-}, riboflavin^{-}NOTCH1^{-}; R^{+}N^{+}, riboflavin^{+}NOTCH1^{+}; the adjusted *P* values were determined using Wald tests with the Benjamini-Hochberg correction in DEseq2 (**B, D**, **E**, **H**, **I, K**).

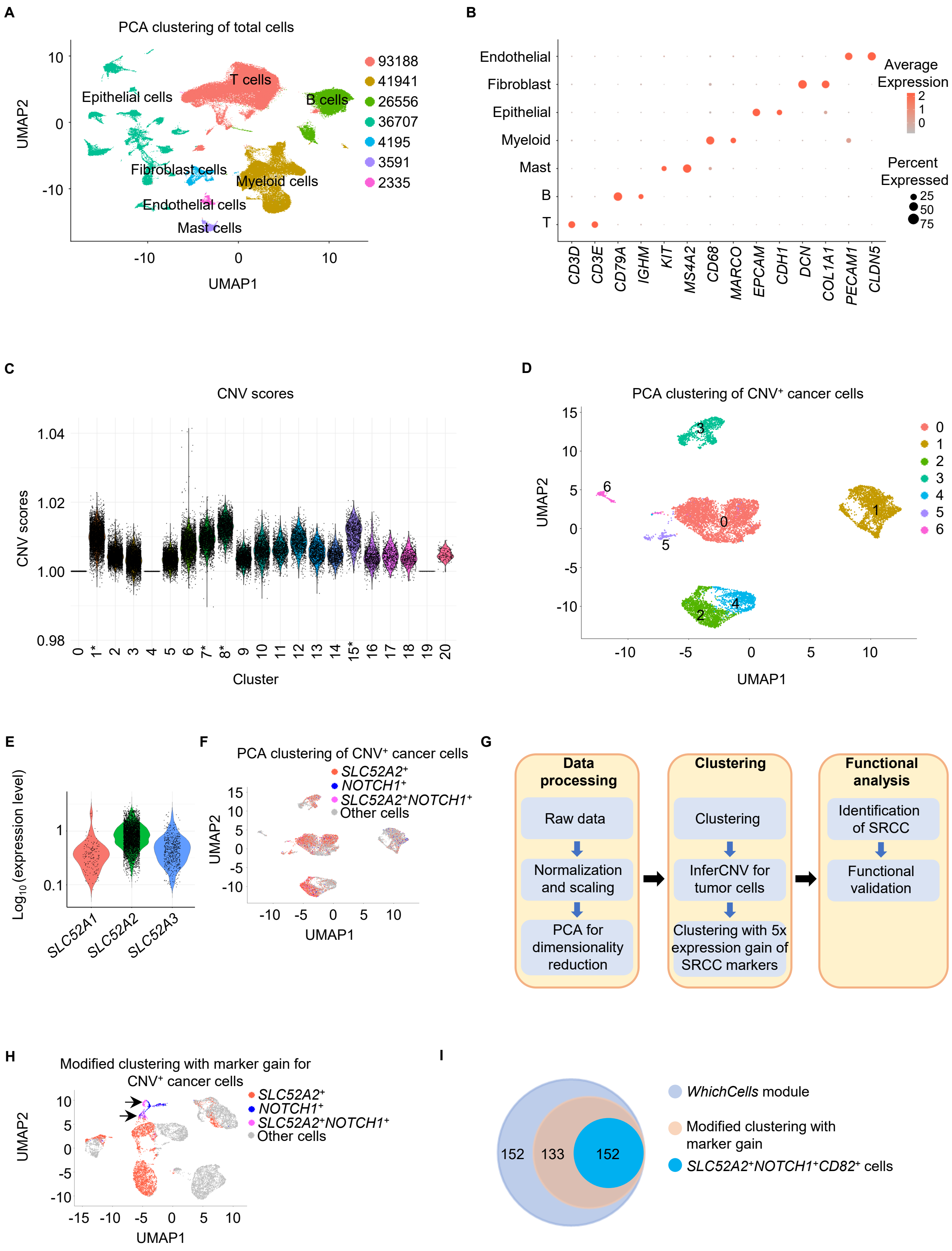

A
PCA clustering of total cells
UMAP2
UMAP1
T cells
Epithelial cells
B cells
Fibroblast cells
Myeloid cells
Endothelial cells
Mast cells
93188
41941
26556
36707
4195
3591
2335
B
Endothelial
Fibroblast
Epithelial
Myeloid
Mast
B
T
CD3D
CD3E
CD79A
IGHM
KIT
MS4A2
CD68
MARCO
EPCAM
CDH1
DCN
COL1A1
PECAM1
CLDN5
Average Expression
Percent Expressed
C
CNV scores
CNV scores
Cluster
D
PCA clustering of CNV⁺ cancer cells
UMAP2
UMAP1
E
Log10(expression level)
SLC52A1
SLC52A2
SLC52A3
F
PCA clustering of CNV⁺ cancer cells
SLC52A2⁺
NOTCH1⁺
SLC52A2⁺NOTCH1⁺
Other cells
UMAP2
UMAP1
G
Data processing
Raw data
Normalization and scaling
PCA for dimensionality reduction
Clustering
Clustering
InferCNV for tumor cells
Clustering with 5x expression gain of SRCC markers
Functional analysis
Identification of SRCC
Functional validation
H
Modified clustering with marker gain for CNV⁺ cancer cells
SLC52A2⁺
NOTCH1⁺
SLC52A2⁺NOTCH1⁺
Other cells
UMAP2
UMAP1
I
152
133
152
WhichCells module
Modified clustering with marker gain
SLC52A2⁺NOTCH1⁺CD82⁺ cells

Figure S4: The *SLC52A2*⁺*NOTCH1*⁺ SRCC population is identified in scRNA-seq data derived from diagnosed NSCLC specimens. A PCA analysis and UMAP plot illustrated cell clusters in the dataset GSE131907 (208506 cells), derived from advanced NSCLC patients before therapy. The cell counts for individual clusters are provided on the right. **B** The expression and positive percentages of lineage marker genes were determined for the cell clusters indicated in Fig. S4A. **C** *InferCNV* analysis of the clustered epithelial, fibroblast and B cells for CNV clusters and their CNV scores (Table S6). The cells in the clusters marked with asterisks were identified as CNV^+ tumor cells with their CNV scores > 1.009, which were used in subsequent experiments (Fig. S4D-I). **D** PCA clustering of the CNV^+ tumor cells. **E** Violin plot showed the expression of *SLC52A1/2/3* in the CNV^+ tumor cells selected by the *WhichCells* module. **F** Visualization of the expression of *SLC52A2* and/or *NOTCH1* on the clusters of CNV^+ tumor cells determined by PCA clustering. **G** Schematic diagram for a modified PCA clustering technique implemented with the 5-fold pre-amplified expression of the marker genes *SLC52A2* and *NOTCH1*, which was developed to enable the clustering of rare SRCC. **H** Visualization of the expression of *SLC52A2* and/or *NOTCH1* on the cell clusters determined by modified PCA analysis with marker gain (Fig. 4A). The black arrows indicate the *SLC52A2*$^+$*NOTCH1*$^+$ clusters. **I** Venn diagram illustrated the *SLC52A2*$^+$*NOTCH1*$^+$ cells identified by the *WhichCells* module and the modified PCA clustering technique with marker gain. The *CD82*$^+$ fraction was also determined in the *SLC52A2*$^+$*NOTCH1*$^+$ cluster identified by the modified PCA clustering technique with marker gain. See also Table S7.

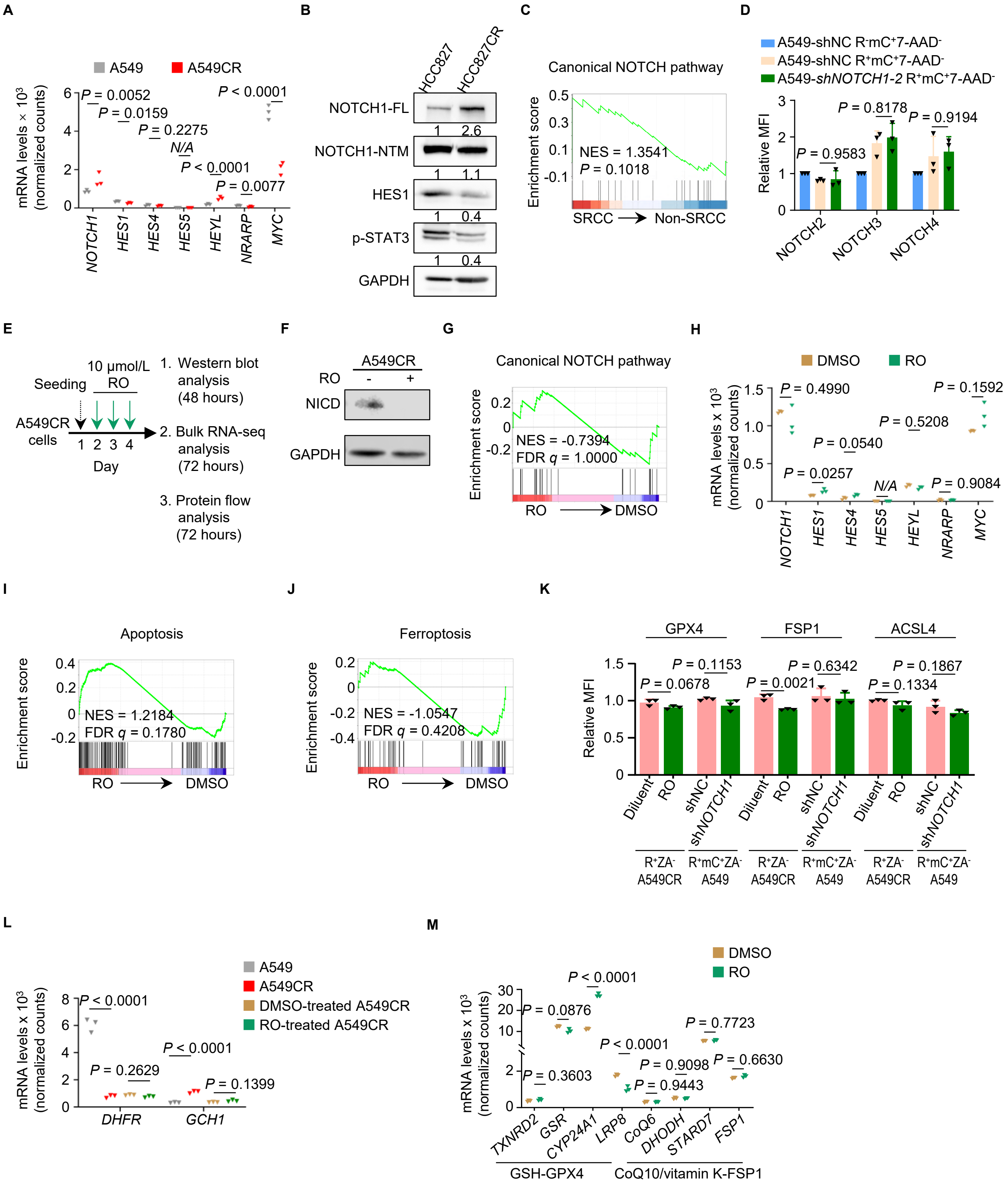

A
A549
A549CR
mRNA levels × 10³ (normalized counts)
P = 0.0052
P = 0.0159
P = 0.2275
N/A
P < 0.0001
P = 0.0077
P < 0.0001
NOTCH1
HES1
HES4
HES5
HEYL
NRARP
MYC
B
HCC827
HCC827CR
NOTCH1-FL
1 2.6
NOTCH1-NTM
1 1.1
HES1
1 0.4
p-STAT3
1 0.4
GAPDH
C
Canonical NOTCH pathway
Enrichment score
NES = 1.3541
P = 0.1018
SRCC
Non-SRCC
D
A549-shNC R⁻mC⁺7-AAD⁻
A549-shNC R⁺mC⁺7-AAD⁻
A549-shNOTCH1-2 R⁺mC⁺7-AAD⁻
Relative MFI
P = 0.9583
P = 0.8178
P = 0.9194
NOTCH2
NOTCH3
NOTCH4
E
10 μmol/L RO
Seeding
A549CR cells
1 2 3 4
Day
1. Western blot analysis (48 hours)
2. Bulk RNA-seq analysis (72 hours)
3. Protein flow analysis (72 hours)
F
A549CR
RO
NICD
GAPDH
G
Canonical NOTCH pathway
Enrichment score
NES = -0.7394
FDR q = 1.0000
RO
DMSO
H
DMSO
RO
mRNA levels x 10³ (normalized counts)
P = 0.4990
P = 0.0257
P = 0.0540
N/A
P = 0.5208
P = 0.9084
P = 0.1592
NOTCH1
HES1
HES4
HES5
HEYL
NRARP
MYC
I
Apoptosis
Enrichment score
NES = 1.2184
FDR q = 0.1780
RO
DMSO
J
Ferroptosis
Enrichment score
NES = -1.0547
FDR q = 0.4208
RO
DMSO
K
GPX4
FSP1
ACSL4
Relative MFI
P = 0.0678
P = 0.1153
P = 0.0021
P = 0.6342
P = 0.1334
P = 0.1867
Diluent
RO
shNC
shNOTCH1
R⁺ZA⁻ A549CR
R⁺mC⁺ZA⁻ A549
L
A549
A549CR
DMSO-treated A549CR
RO-treated A549CR
mRNA levels x 10³ (normalized counts)
P < 0.0001
P = 0.2629
P < 0.0001
P = 0.1399
DHFR
GCH1
M
DMSO
RO
mRNA levels x 10³ (normalized counts)
P = 0.3603
P = 0.0876
P < 0.0001
P < 0.0001
P = 0.9098
P = 0.9443
P = 0.7723
P = 0.6630
TXNRD2
GSR
CYP24A1
LRP8
CoQ6
DHODH
STARD7
FSP1
GSH-GPX4
CoQ10/vitamin K-FSP1

Figure S5: The non-canonical NOTCH pathway plays an important role in the regulation of SRCC. A Transcriptional comparison of the canonical NOTCH pathway targets between A549CR and A549 cells (n = 3 independent experiments). **B** Western blot analysis of key regulators in the canonical NOTCH pathway in the comparison of HCC827CR cells vs. HCC827 cells (n = 2 independent experiments). FL, full length, NTM, NOTCH transmembrane and intracellular region. **C** GSEA of scRNA-seq data using *GSEABase* and *fgsea* illustrated that the canonical NOTCH pathway was not enriched in SRCC compared to non-SRCC. **D** Flow cytometric analysis of NOTCH2, NOTCH3 and NOTCH4 in the surface of riboflavin$^+$mCherry$^+$7-AAD$^-$ A549-shNC and A549-*shNOTCH1*-2 cells (n = 3 independent experiments). Both A549-shNC and A549-*shNOTCH1-2* are positive for mCherry. **E** Schematic diagram for assessing the response of A549CR cells to the RO treatment for 48 hours (Western blot analysis), or 72 hours (bulk RNA-seq analysis, or protein-flow analysis). **F** Western blot analysis of the NICD protein in response to treatment with 10 μmol/L RO for 48 hours (n = 3 independent experiments). **G** GSEA of bulk RNA-seq data illustrated that the canonical NOTCH pathway was not activated in response to treatment with 10 μmol/L RO for 72 hours (n = 3 independent experiments). **H** Transcriptional evaluation of the canonical NOTCH pathway targets in response to RO, using bulk RNA-seq analysis with DEseq2 (n = 3 independent experiments). **I,J** GSEA assessed the correlation of the apoptotic (**I**) and ferroptotic (**J**) programs with RO treatment in A549CR cells (n = 3 independent experiments). **K** Protein-flow analysis with the monoclonal antibodies (230168A8 for GPX4 expression, 1A2B2 for FSP1 expression, and 1H5D3 for ACSL4 expression) in riboflavin$^+$ZA$^-$ A549CR cells treated with RO for 72 hours, or in riboflavin$^+$mCherry$^+$ZA$^-$ A549-sh*NOTCH*1-2 cells (n = 3 independent experiments). Both A549-shNC and A549-*shNOTCH1-2* are positive for mCherry. **L** Transcriptional comparison of the BH4-DHFR regulatory genes *DHFR* and *GCH1* between A549CR and A549 cells, and between RO- and DMSO-treated A549CR cells, using bulk RNA-seq analysis with DEseq2 (n = 3 independent experiments). **M** Transcriptional evaluation of the GSH-GPX4 regulatory genes *TXNRD2, GSR*, *CYP24A1*, *LRP8*, and the CoQ10 (and vitamin K)-FSP1 regulatory genes *CoQ6*, *DHODH, STARD7* and *FSP1*, in A549CR cells in response to RO (n = 3 independent experiments). The adjusted *P* values were determined using Wald tests with the Benjamini-Hochberg correction in DEseq2 (see also Fig. S3B, and S3K). RO, RO4929097; R, riboflavin; mC, mCherry for *shNOTCH1*-2; ZA, Zombie Aqua fluorescence for dead cells. The data are presented as mean ± SD. *P* values were calculated by one way ANOVA with Tukey's tests (**D**), or Student's unpaired *t*-tests (**K**); the adjusted *P* values were determined using Wald tests with the Benjamini-Hochberg correction in DEseq2 (**A**, **H**, **L**, **M**).

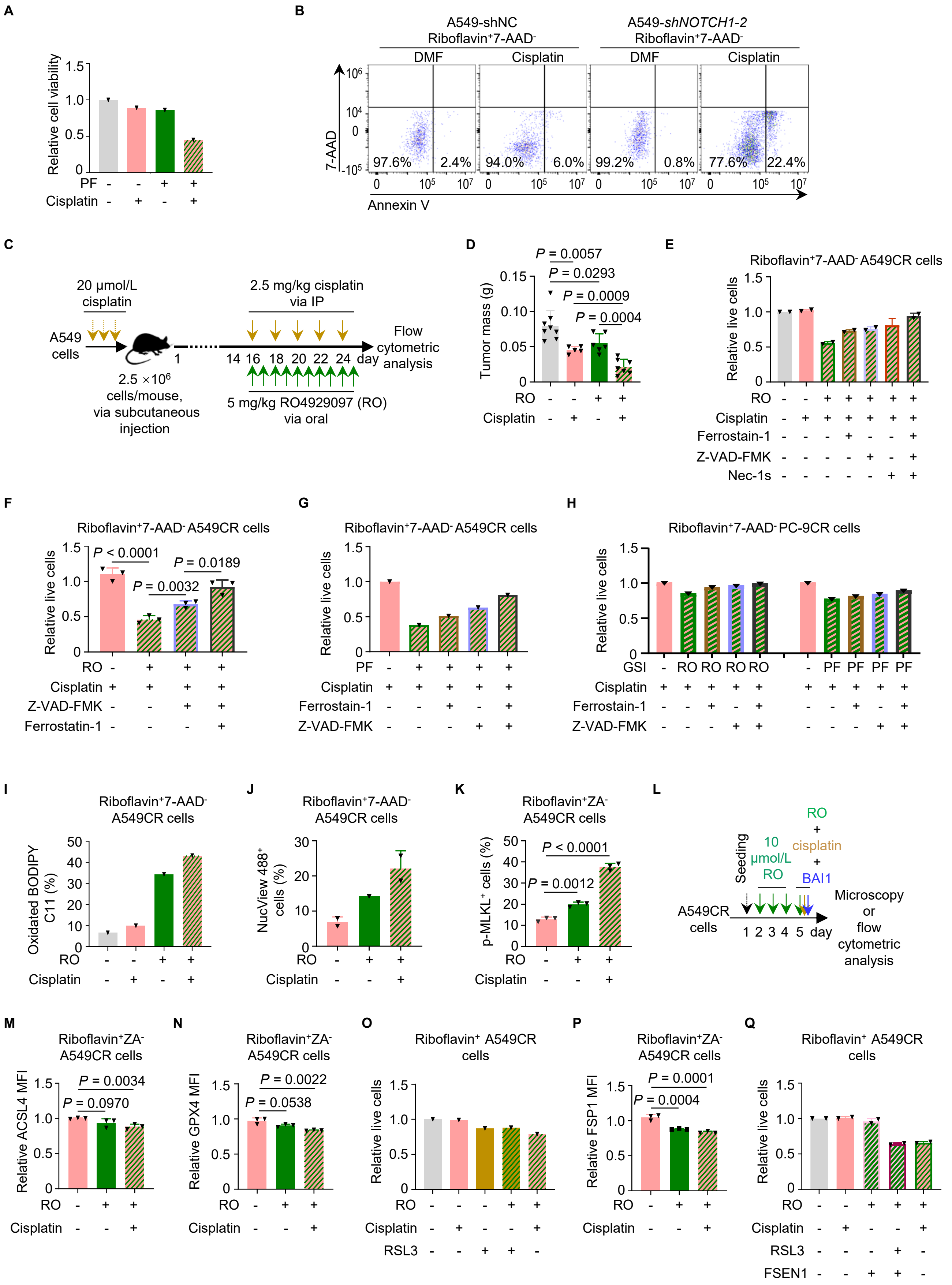
A
Relative cell viability
PF
Cisplatin
B
A549-shNC
Riboflavin+7-AAD-
A549-shNOTCH1-2
Riboflavin+7-AAD-
DMF
Cisplatin
DMF
Cisplatin
7-AAD
97.6%
2.4%
94.0%
6.0%
99.2%
0.8%
77.6%
22.4%
Annexin V
C
20 μmol/L cisplatin
A549 cells
2.5 ×10^6 cells/mouse, via subcutaneous injection
2.5 mg/kg cisplatin via IP
14 16 18 20 22 24 day
Flow cytometric analysis
5 mg/kg RO4929097 (RO) via oral
D
Tumor mass (g)
P = 0.0057
P = 0.0293
P = 0.0009
P = 0.0004
RO
Cisplatin
E
Riboflavin+7-AAD- A549CR cells
Relative live cells
RO
Cisplatin
Ferrostain-1
Z-VAD-FMK
Nec-1s
F
Riboflavin+7-AAD- A549CR cells
Relative live cells
P < 0.0001
P = 0.0189
P = 0.0032
RO
Cisplatin
Z-VAD-FMK
Ferrostatin-1
G
Riboflavin+7-AAD- A549CR cells
Relative live cells
PF
Cisplatin
Ferrostain-1
Z-VAD-FMK
H
Riboflavin+7-AAD- PC-9CR cells
Relative live cells
GSI
RO RO RO RO
PF PF PF PF
Cisplatin
Ferrostain-1
Z-VAD-FMK
I
Riboflavin+7-AAD- A549CR cells
Oxidated BODIPY C11 (%)
RO
Cisplatin
J
Riboflavin+7-AAD- A549CR cells
NucView 488+ cells (%)
RO
Cisplatin
K
Riboflavin+ZA- A549CR cells
p-MLKL+ cells (%)
P < 0.0001
P = 0.0012
RO
Cisplatin
L
Seeding
10 μmol/L RO
RO + cisplatin + BAI1
A549CR cells
1 2 3 4 5 day
Microscopy or flow cytometric analysis
M
Riboflavin+ZA- A549CR cells
Relative ACSL4 MFI
P = 0.0034
P = 0.0970
RO
Cisplatin
N
Riboflavin+ZA- A549CR cells
Relative GPX4 MFI
P = 0.0022
P = 0.0538
RO
Cisplatin
O
Riboflavin+ A549CR cells
Relative live cells
RO
Cisplatin
RSL3
P
Riboflavin+ZA- A549CR cells
Relative FSP1 MFI
P = 0.0001
P = 0.0004
RO
Cisplatin
Q
Riboflavin+ A549CR cells
Relative live cells
RO
Cisplatin
RSL3
FSEN1

Fig. S6: Inhibition of NOTCH1 re-sensitizes SRCC to cisplatin-mediated cytotoxicity. A The viability of A549CR cells in response to cisplatin, PF, or both, was assessed using CCK-8 assays (n = 1 independent experiment). PF is a GSI that inhibits NOTCH signaling. **B** Representative Annexin V assay for the effect of cisplatin on riboflavin$^+$7-AAD$^-$ A549-shNC and A549-*shNOTCH1*-2 cells (n = 3 independent experiments). Please also see summarized data in Fig. 6E. **C** Experimental design for assessing the effect of RO, cisplatin, or both on SRCC and xenograft tumors. **D** Evaluation of tumor mass in xenograft mice treated with RO alone, cisplatin alone, or both (n = 5-8 mice per group). **E** Annexin V assays for the effect of the ferroptosis inhibitor ferrostatin-1 (10 μmol/L), the apoptosis inhibitor Z-VAD-FMK (10 μmol/L), and the necroptosis inhibitor Nec-1s (10 μmol/L) on the death of riboflavin$^+$7-AAD$^-$ SRCC induced by the combinatorial treatment of RO and cisplatin (n = 2 independent experiments). See also representative assays in Fig. 6H. **F** Annexin V assays for the effect of the ferroptosis inhibitor ferrostatin-1 (10 μmol/L) and the apoptosis inhibitor Z-VAD-FMK (10 μmol/L) on the death of riboflavin$^+$7-AAD$^-$ SRCC induced by the combinatorial treatment of RO and cisplatin (n = 3 independent experiments). **G** An Annexin V assay for the effect of the ferroptosis inhibitor ferrostatin-1 (10 μmol/L) and the apoptosis inhibitor Z-VAD-FMK (10 μmol/L) on the death of the riboflavin$^+$7-AAD$^-$ A549CR cells induced by the combinatorial treatment of PF and cisplatin (n = 1 independent experiment). **H** Annexin V assay for the effect of the ferroptosis inhibitor ferrostatin-1 (10 μmol/L) and the apoptosis inhibitor Z-VAD-FMK (10 μmol/L) on the death of the riboflavin$^+$7-AAD$^-$ PC-9CR cells induced by cisplatin in combination with RO (left panel, n = 1 independent experiment) or PF (right panel, n = 1 independent experiment). **I** Flow cytometric analysis of the oxidated BODIPY 581/591 C11 fluorescence in riboflavin$^+$7-AAD$^-$ A549CR cells in response to RO, cisplatin, or both (n = 1 independent experiment). **J** Flow cytometric analysis for the cell fraction positive for activated caspase-3 (indicated by the fluorescence of NucView 488) in the riboflavin$^+$7-AAD$^-$ A549CR cells in response to the 24-hour treatment with RO and cisplatin (n = 2 independent experiments). **K** Protein-flow analysis with the the monoclonal antibody EPR9514 for detecting the cell fraction positive for phosphorylated MLKL (S358) in the riboflavin$^+$ZA$^-$ A549CR compartment in response to the 24-hour treatment with RO and cisplatin (n = 3 independent experiments). **L** Schematic for assessing the lysosomal integrity of SRCC treated with RO and cisplatin or with RO, cisplatin, and BAI1. **M** Protein-flow analysis with the monoclonal antibody 1H5D3 for ACSL4 expression in riboflavin$^+$ZA$^-$ A549CR cells treated with RO and/or cisplatin (n = 3 independent experiments). **N** Protein-flow analysis with the monoclonal antibody 230168A8 for GPX4 expression in riboflavin$^+$ZA$^-$ A549CR cells treated with cisplatin and RO for 24 hours (n = 3 independent experiments). **O** Annexin V assay for assessing the 24-hour effect of RSL3 on riboflavin$^+$ A549CR cells in the presence of RO (n = 1 independent experiment). **P** Protein-flow analysis with the monoclonal antibody 1A2B2 for FSP1 expression in riboflavin$^+$ZA$^-$ A549CR cells treated with cisplatin and RO for 24 hours (n = 3 independent experiments). **Q** Annexin V assays for assessing the 48-hour effect of RSL3 and/or FSEN1 on riboflavin$^+$ A549CR cells in the presence of RO (n = 2 independent experiments). PF, PF-03084014; RO, RO4929097. ZA, Zombie Aqua fluorescence for dead cells. The data are presented as mean ± SD. *P* values were calculated by Student's unpaired *t*-tests (**D**) or one way ANOVA with Tukey's tests (**F**, **K**, **M**, **N**, **P**).
