# Supplementary material for: Non-canonical NOTCH1 signaling regulates ferroptosis vulnerability in dormant lung cancer cells with stable resistance
Source: Cell Death Dis. 2025 Dec 26;17(1):1. doi: 10.1038/s41419-025-08355-9 (PMC12780219; doi:10.1038/s41419-025-08355-9)
Supplement: Supplementary file 3 — Supplementary Table 1 [file 41419_2025_8355_MOESM3_ESM.pdf]

Table S1. Patient information and fractions of SRCC in NSCLC tumors

Related to Fig. 1H, Fig. 2H, Fig. 3L, 3N, 3O, S3G

Patient ID	Age	Gender	Date of enrollment	Hospital	Tumor type	Staging	Mutation	Therapeutic regimen**	Therapeutic strategy	Duration of treatment (days)	Pathologic response***	% SRCC
PLC001	49	Male	20240518	ZJU-FAH*	Lung squamous carcinoma	T3bN2M0	N/A	Daboshu 200 mg + paclitaxel albumin 300 mg + carboplatin 500 mg, 3 cycles	Immunotherapy + chemotherapy	63	NR	2.160%
PLC002	68	Female	20240524	ZJU-FAH	Lung adenocarcinoma	T2aN2M0	EGFR ex19del	80 mg furmonertinib (oral) daily for 3 months	Targeted therapy	90	NR	2.030%
PLC003	66	Male	20240705	ZJU-FAH	Lung squamous carcinoma	T1cN2M0	N/A	Sintilimab 200 mg+ carboplatin 500 mg, 4 cycles	Immunotherapy + chemotherapy	84	CPR	0.140%
PLC004	64	Male	20240717	ZJU-FAH	Lung squamous carcinoma	T2aN2M0	N/A	Pembrolizumab 200 mg + paclitaxel albumin 400 mg, 4 cycles	Immunotherapy + chemotherapy	84	CPR	1.080%
PLC005	65	Male	20240717	ZJU-FAH	Lung squamous carcinoma	T3N2bM0	N/A	Tislelizumab 200 mg + paclitaxel albumin 450 mg + carboplatin 400 mg, 3 cycles	Immunotherapy + chemotherapy	63	NR	2.200%
PLC006	68	Male	20240807	ZJU-FAH	Lung squamous carcinoma	T2aN2M0	N/A	Sintilimab 200 mg + paclitaxel albumin 400 mg + carboplatin 400 mg, 3 cycles	Immunotherapy + chemotherapy	63	MPR	0.090%
PLC007	71	Male	20241108	ZJU-FAH	Lung squamous carcinoma	T1bN0M0	N/A	Paclitaxel albumin 400 mg + carboplatin 450 mg + tislelizumab 200 mg, 2 cycles	Immunotherapy + chemotherapy	51	MPR	0.180%
PLC008	61	Male	20241212	ZJU-FAH	Lung squamous carcinoma	T2aN0Mx	N/A	N/A	N/A	N/A	N/A	0.004%
PLC009	71	Male	20241212	ZJU-FAH	Lung adenocarcinoma	T1cN1Mx	N/A	N/A	N/A	N/A	N/A	0.034%
PLC010	74	Female	20241216	ZJU-FAH	Lung adenocarcinoma	T2aN2Mx	N/A	N/A	N/A	N/A	N/A	0.027%
PLC011	71	Male	20250421	ZJU-FAH	Lung adenocarcinoma	pT2N2Mx	PD-L1+	N/A	Immunotherapy	N/A	N/A	N/A
PLC012	61	Female	20250430	ZJU-FAH	Lung adenocarcinoma	ypT1bN1Mx	EGFR 19del	80 mg Osimertinib (oral) daily for 3 months	Targeted therapy	90	MPR	0.280%
PLC013	73	Male	20250504	ZJU-FAH	Lung adenocarcinoma	T1bN0Mx	EGFR L858R	N/A	N/A	N/A	N/A	0.090%
PLC014	68	Female	20250512	ZJU-FAH	Lung adenocarcinoma	pT1N0M0	N/A	N/A	N/A	N/A	N/A	0.030%

Footnote:

*ZJU-FAH, the First Affiliated Hospital, College of Medicine, Zhejiang University, Hangzhou, China

**， a cycle of immunotherapy and chemotherapy = 1 day administration and 20 day recovery without treatment

***，CPR, complete pathologic response; MPR, major pathologic response (defined as less than 10% residual tumor under microscopy) ；NR, non-response (failure to achieve CPR or MPR).
