# Supplementary material for: Non-canonical NOTCH1 signaling regulates ferroptosis vulnerability in dormant lung cancer cells with stable resistance
Source: Cell Death Dis. 2025 Dec 26;17(1):1. doi: 10.1038/s41419-025-08355-9 (PMC12780219; doi:10.1038/s41419-025-08355-9)
Supplement: Supplementary file 4 — Supplementary Table 2 [file 41419_2025_8355_MOESM4_ESM.pdf]

Huang H et. al., Non-canonical NOTCH1 Signaling Regulates Ferroptosis Vulnerability in Dormant Lung Cancer Cells with Stable Resistance

Table S2. GSEA with multiple gene sets for bulk RNA-seq and scRNA-seq data

Tab 1 : A549CR vs A549

This tab is related to Fig. 3A-F, H, I, Fig. 5A

GSEA was conducted for bulk RNA-seq data of A549CR vs A549

Gene set	Size	ES	NES	NOM p-val	FDR q-val	Fwer p-val	Rank at max	Leading edge	Reference link
**Chemoresistance**									
KERLEY_RESPONSE_TO_CISPLATIN_UP	45	0.83363	2.09407	0	0	0	2415	tags=62%, list=7%, signal=67%	https://www.gsea-msigdb.org/gsea/msigdb/cards/KERLEY_RESPONSE_TO_CISPLATIN_UP
BRACHAT_RESPONSE_TO_CISPLATIN	23	0.79124	1.7287	0	0.003874736	0.013	2659	tags=39%, list=8%, signal=42%	https://www.gsea-msigdb.org/gsea/msigdb/cards/BRACHAT_RESPONSE_TO_CISPLATIN.html
KANG_FLUOROURACIL_RESISTANCE_UP	22	0.75752	1.65407	0.005093379	0.006778709	0.035	1608	tags=41%, list=5%, signal=43%	https://www.gsea-msigdb.org/gsea/msigdb/cards/KANG_FLUOROURACIL_RESISTANCE_UP.html
KANG_CISPLATIN_RESISTANCE_UP	21	0.70054	1.50383	0.020134227	0.031257994	0.194	5431	tags=62%, list=15%, signal=73%	https://www.gsea-msigdb.org/gsea/msigdb/human/geneset/KANG_CISPLATIN_RESISTANCE_UP.html?ex=1
WANG_CISPLATIN_RESPONSE_AND_XPC_DN	218	0.4592	1.44648	0.001386963	0.047914475	0.331	5540	tags=40%, list=16%, signal=47%	https://www.gsea-msigdb.org/gsea/msigdb/geneset_page.jsp?geneSetName=WANG_CISPLATIN_RESPONSE_AND_XPC_DN
**Ferroptosis**									
WP_FERROPTOSIS	63	-0.37001	-1.06524	0.32142857	0.44611192	0.539	5558	tags=43%, list=16%, signal=51%	https://www.gsea-msigdb.org/gsea/msigdb/cards/WP_FERROPTOSIS.html
**Senescence**									
SENMAYO	124	0.32266	0.938	0.6281113	0.6281113	0.429	4183	tags=24%, list=12%, signal=27%	https://pubmed.ncbi.nlm.nih.gov/35974106/
**G0 arrest**									
G0-ARREST-UP	25	0.7873	1.72915	0	0	0	3643	tags=64%, list=10%, signal=71%	https://pubmed.ncbi.nlm.nih.gov/37221612/
G0-ARREST-DOWN	111	-0.80591	-2.49017	0	0	0	5130	tags=77%, list=14%, signal=90%	
**Hallmark**									
HALLMARK_APOPTOSIS	161	0.42493	1.27934	0.030683404	0.33699375	0.836	4148	tags=32%, list=12%, signal=36%	https://www.gsea-msigdb.org/gsea/msigdb/cards/HALLMARK APOPTOSIS.html
HALLMARK_EPITHELIAL_MESENCHYMAL_TRANSITION	200	-0.50122	-1.68794	0	0.003877405	0.014	4300	tags=42%, list=12%, signal=48%	https://www.gsea-msigdb.org/gsea/msigdb/cards/HALLMARK EPITHELIAL MESENCHYMAL TRANSITION
HALLMARK_E2F_TARGETS	200	-0.84711	-2.83069	0	0	0	2901	tags=73%, list=8%, signal=79%	https://www.gsea-msigdb.org/gsea/msigdb/cards/HALLMARK E2F TARGETS
HALLMARK_G2M_CHECKPOINT	200	-0.83069	-2.79543	0	0	0	2677	tags=63%, list=8%, signal=68%	https://www.gsea-msigdb.org/gsea/msigdb/cards/HALLMARK G2M CHECKPOINT
HALLMARK_MYC_TARGETS_V1	200	-0.71515	-2.35817	0	0	0	5537	tags=66%, list=16%, signal=77%	https://www.gsea-msigdb.org/gsea/msigdb/cards/HALLMARK MYC TARGETS V1
HALLMARK_MITOTIC_SPINDLE	199	-0.6578	-2.18382	0	0	0	2756	tags=35%, list=8%, signal=38%	https://www.gsea-msigdb.org/gsea/msigdb/cards/HALLMARK MITOTIC SPINDLE
HALLMARK_MTORC1_SIGNALING	200	-0.49669	-1.63332	0	0.005163916	0.022	5247	tags=49%, list=15%, signal=57%	https://www.gsea-msigdb.org/gsea/msigdb/cards/HALLMARK MTORC1 SIGNALING
HALLMARK_P53_PATHWAY	200	0.69842	2.14862	0	0	0	3630	tags=52%, list=10%, signal=57%	https://www.gsea-msigdb.org/gsea/msigdb/cards/HALLMARK P53 PATHWAY
HALLMARK_NOTCH_SIGNALING	32	0.4018	0.96397	0.49172187	0.63840604	1	7491	tags=38%, list=21%, signal=48%	https://www.gsea-msigdb.org/gsea/msigdb/cards/HALLMARK_NOTCH_SIGNALING

Tab 2 : SRCC vs Non-SRCC

This tab is related to Fig. 4C-F, Fig. S5C

GSEA was used to compare the SRCC population and a non-SRCC population in scRNA-seq data

Gene set	p-val	NES	eference link
**Ferroptosis**			https://www.gsea-msigdb.org/gsea/msigdb/cards/HALLMARK APOPTOSIS.html
WP_FERROPTOSIS	0.92493	0.68401	https://www.gsea-msigdb.org/gsea/msigdb/cards/WP_FERROPTOSIS.html
**Hallmark**			
HALLMARK_APOPTOSIS	0.76132	-0.88422	https://www.gsea-msigdb.org/gsea/msigdb/cards/HALLMARK APOPTOSIS.html
HALLMARK_EPITHELIAL_MESENCHYMAL_TRANSITION	0.02175	-1.36803	https://www.gsea-msigdb.org/gsea/msigdb/cards/HALLMARK EPITHELIAL MESENCHYMAL TRANSITION
HALLMARK_E2F_TARGETS	5.94E-10	-2.26814	https://www.gsea-msigdb.org/gsea/msigdb/cards/HALLMARK E2F TARGETS
HALLMARK_G2M_CHECKPOINT	2.13E-08	-2.19504	https://www.gsea-msigdb.org/gsea/msigdb/cards/HALLMARK G2M CHECKPOINT
HALLMARK_MITOTIC_SPINDLE	0.00131	-1.59518	https://www.gsea-msigdb.org/gsea/msigdb/cards/HALLMARK_MITOTIC_SPINDLE
HALLMARK_MTORC1_SIGNALING	0.01062	-1.45628	https://www.gsea-msigdb.org/gsea/msigdb/cards/HALLMARK MTORC1 SIGNALING
HALLMARK_NOTCH_SIGNALING	0.1018	1.35412	https://www.gsea-msigdb.org/gsea/msigdb/cards/HALLMARK NOTCH SIGNALING
HALLMARK_HYPOXIA	9.1E-09	-2.19287	https://www.gsea-msigdb.org/gsea/msigdb/cards/HALLMARK HYPOXIA

Tab 3 : A549CR RO vs A549CR DMSO

This tab is related to Fig. 5C, Fig. S5G, I, J

GSEA was conducted for bulk RNA-seq data of A549CR cells treated with RO or DMSO

Gene set	Size	ES	NES	NOM p-val	FDR q-val	Fwer p-val	Rank at max	Leading edge	Reference link
**Ferroptosis**									
WP FERROPTOSIS	54	-0.38299	-1.03386	0.3900185	0.7485127	0.674	3090	tags=24%, list=9%, signal=26%	https://www.gsea-msigdb.org/gsea/msigdb/cards/WP_FERROPTOSIS.html
**Hallmark**									
HALLMARK NOTCH SIGNALING	26	-0.30865	-0.73943	0.91840607	1	1	2303	tags=23%, list=7%, signal=25%	https://www.gsea-msigdb.org/gsea/msigdb/cards/HALLMARK NOTCH SIGNALING
HALLMARK MYC TARGETS V1	177	0.67709	2.21063	0	0	0	7580	tags=75%, list=21%, signal=94%	https://www.gsea-msigdb.org/gsea/msigdb/cards/HALLMARK MYC TARGETS V1
HALLMARK EPITHELIAL MESENCHYMAL TRANSITION	170	0.55713	1.8162	0	0.002758487	0.005	6358	tags=50%, list=18%, signal=61%	https://www.gsea-msigdb.org/gsea/msigdb/cards/HALLMARK EPITHELIAL MESENCHYMAL TRANSITION
HALLMARK E2F TARGETS	160	0.46639	1.49617	0	0.022594685	0.11	8972	tags=60%, list=25%, signal=80%	https://www.gsea-msigdb.org/gsea/msigdb/cards/HALLMARK E2F TARGETS
HALLMARK G2M CHECKPOINT	166	0.42434	1.36774	0.004424779	0.055576883	0.369	6780	tags=39%, list=19%, signal=48%	https://www.gsea-msigdb.org/gsea/msigdb/cards/HALLMARK G2M CHECKPOINT
HALLMARK_MITOTIC_SPINDLE	143	-0.33236	-1.03731	0.34219858	1	1	8988	tags=51%, list=25%, signal=68%	https://www.gsea-msigdb.org/gsea/msigdb/cards/HALLMARK_MITOTIC_SPINDLE
HALLMARK APOPTOSIS	134	0.38807	1.21837	0.07013575	0.17801638	0.865	7763	tags=46%, list=22%, signal=59%	https://www.gsea-msigdb.org/gsea/msigdb/cards/HALLMARK APOPTOSIS.html

Footnote:

1. In the GSEA reports, a p value of zero indicates an actual p value of less than 1/1000 permutations.
