# Supplementary material for: Non-canonical NOTCH1 signaling regulates ferroptosis vulnerability in dormant lung cancer cells with stable resistance
Source: Cell Death Dis. 2025 Dec 26;17(1):1. doi: 10.1038/s41419-025-08355-9 (PMC12780219; doi:10.1038/s41419-025-08355-9)
Supplement: Supplementary file 5 — Supplementary Table 3 [file 41419_2025_8355_MOESM5_ESM.pdf]

Huang H et. al. Non-canonical NOTCH1 Signaling Regulates Ferroptosis Vulnerability in Dormant Lung Cancer Cells with Stable Resistance

Table S3. Chemoresistance-related DEG in SRCC

Tab1 : A549CR vs A549

This tab is related to Fig. S3A

No.	Gene name	Reported expression changes in platinum resistance*	DEG in A549CR vs A549
1	KCNMA1	Downregulated	Downregulated
2	NTRK3	Downregulated	Downregulated
3	FANCI	Downregulated	Downregulated
4	BLM	Downregulated	Downregulated
5	THBS1	Downregulated	Downregulated
6	SLFN11	Downregulated	Downregulated
7	MSH6	Downregulated	Downregulated
8	MSH2	Downregulated	Downregulated
9	BRIP1	Downregulated	Downregulated
10	ITPR1	Downregulated	Downregulated
11	TIMP2	Downregulated	Downregulated
12	PDXK	Downregulated	Downregulated
13	RBMS3	Downregulated	Downregulated
14	WWOX	Downregulated	Downregulated
15	SLC3A2	Downregulated	Downregulated
16	BOK	Downregulated	Downregulated
17	TGFBI	Downregulated	Downregulated
18	NF2	Downregulated	Downregulated
19	CDH1	Downregulated	Downregulated
20	VIM	Downregulated	Downregulated
21	LRRC8D	Downregulated	Downregulated
22	TIMP3	Downregulated	Downregulated
23	ATP1B1	Downregulated	Downregulated
24	AIFM1	Downregulated	Downregulated
25	NEDD4L	Downregulated	Downregulated
26	ARID1A	Downregulated	Downregulated
27	RBM3	Downregulated	Downregulated
28	TRAP1	Downregulated	Downregulated
29	NEK11	Downregulated	Downregulated
30	RGS10	Downregulated	Downregulated
31	BIN1	Downregulated	Downregulated
32	DICER1	Downregulated	Downregulated
33	LRRC8A	Downregulated	Downregulated
34	DAPK3	Downregulated	Downregulated
35	SLC31A1	Downregulated	Downregulated
36	SETD2	Downregulated	Downregulated
37	SLC22A3	Downregulated	Downregulated
38	DYRK2	Downregulated	Downregulated
39	PTGS2	Upregulated	Upregulated
40	MDM2	Upregulated	Upregulated
41	CRYAB	Upregulated	Upregulated
42	CYP1B1	Upregulated	Upregulated

43	GDF15	Upregulated	Upregulated
44	PDK4	Upregulated	Upregulated
45	PPM1D	Upregulated	Upregulated
46	SOX8	Upregulated	Upregulated
47	SNAI2	Upregulated	Upregulated
48	XPC	Upregulated	Upregulated
49	ACTA2	Upregulated	Upregulated
50	PMEL	Upregulated	Upregulated
51	SQSTM1	Upregulated	Upregulated
52	TWIST1	Upregulated	Upregulated
53	CD55	Upregulated	Upregulated
54	PPARGC1A	Upregulated	Upregulated
55	POLH	Upregulated	Upregulated
56	ALDH3A1	Upregulated	Upregulated
57	NAP1L3	Upregulated	Upregulated
58	IKBKB	Upregulated	Upregulated
59	GADD45A	Upregulated	Upregulated
60	PSMB10	Upregulated	Upregulated
61	DKK1	Upregulated	Upregulated
62	RAD52	Upregulated	Upregulated
63	SFN	Upregulated	Upregulated
64	PBXIP1	Upregulated	Upregulated
65	FANCF	Upregulated	Upregulated
66	MMP7	Upregulated	Upregulated
67	SMARCA2	Upregulated	Upregulated
68	TEAD3	Upregulated	Upregulated
69	PIK3R1	Upregulated	Upregulated
70	EPHA2	Upregulated	Upregulated
71	ATP6V1A	Upregulated	Upregulated
72	XPA	Upregulated	Upregulated
73	GSN	Upregulated	Upregulated
74	PRR13	Upregulated	Upregulated
75	ALKBH3	Upregulated	Upregulated
76	CUEDC2	Upregulated	Upregulated
77	HSF1	Upregulated	Upregulated
78	TXNDC17	Upregulated	Upregulated
79	TNFAIP8	Upregulated	Upregulated
80	HBEGF	Upregulated	Upregulated
81	ABCC3	Upregulated	Upregulated
82	CFLAR	Upregulated	Upregulated
83	GSTP1	Upregulated	Upregulated
84	KLF5	Upregulated	Upregulated
85	SIX1	Upregulated	Upregulated
86	SOX9	Upregulated	Upregulated
87	SERPINE1	Upregulated	Upregulated
88	ARIH1	Upregulated	Upregulated
89	NOTCH3	Upregulated	Upregulated
90	CARD10	Upregulated	Upregulated
91	NAPSA	Upregulated	Upregulated
92	ABCC5	Upregulated	Upregulated
93	YAP1	Upregulated	Upregulated
94	CLDN4	Upregulated	Upregulated
95	SIRT3	Upregulated	Upregulated
96	ERCC5	Upregulated	Upregulated
97	KDM3A	Upregulated	Upregulated

98	CD44	Upregulated	Upregulated
99	DVL2	Upregulated	Upregulated
100	EDNRA	Upregulated	Upregulated
101	IL7	Upregulated	Upregulated
102	DVL3	Upregulated	Upregulated
103	ABCA8	Upregulated	Upregulated
104	USP35	Upregulated	Upregulated
105	HSPB1	Upregulated	Upregulated
106	SERPINB3	Upregulated	Upregulated
107	RELA	Upregulated	Upregulated
108	RAD51C	Upregulated	Upregulated
109	ID1	Upregulated	Upregulated
110	HCFC1R1	Upregulated	Upregulated
111	PTPN3	Upregulated	Upregulated
112	ATG14	Upregulated	Upregulated
113	ERCC1	Upregulated	Upregulated
114	ACSS2	Upregulated	Upregulated
115	NOTCH1	Upregulated	Upregulated
116	STAT3	Upregulated	Upregulated
117	ABRAXAS2	Upregulated	Upregulated
118	PAK1	Upregulated	Upregulated
119	GPX4	Upregulated	Upregulated
120	MVP	Upregulated	Upregulated
121	ITGB8	Upregulated	Upregulated
122	H6PD	Upregulated	Upregulated
123	ITGA6	Upregulated	Upregulated
124	PPL	Upregulated	Upregulated
125	TBX2	Upregulated	Upregulated
126	HELQ	Upregulated	Upregulated

Tab2 : SRCC vs non-SRCC

This tab is related to Fig. 4B

No.	Gene name	Reported expression changes in platinum resistance*	DEG in SRCC vs non-SRCC
1	SMPD1	Downregulated	Downregulated
2	AIFM1	Downregulated	Downregulated
3	NKX2-8	Downregulated	Downregulated
4	IRF1	Downregulated	Downregulated
5	MLH1	Downregulated	Downregulated
6	PDCD4	Downregulated	Downregulated
7	MOAP1	Downregulated	Downregulated
8	BHLHE40	Downregulated	Downregulated
9	SLC3A2	Downregulated	Downregulated
10	PML	Downregulated	Downregulated
11	MAPK13	Downregulated	Downregulated
12	BOK	Downregulated	Downregulated
13	LRRC8A	Downregulated	Downregulated
14	CD40	Downregulated	Downregulated
15	TNFSF10	Downregulated	Downregulated
16	MSH2	Downregulated	Downregulated
17	TP53	Downregulated	Downregulated
18	CASP3	Downregulated	Downregulated
19	NOTCH1	Upregulated	Upregulated

20	ST6GAL1	Upregulated	Upregulated
21	HOXB3	Upregulated	Upregulated
22	MGMT	Upregulated	Upregulated
23	FN1	Upregulated	Upregulated
24	BMI1	Upregulated	Upregulated
25	PTK2	Upregulated	Upregulated
26	MTRR	Upregulated	Upregulated
27	HSF1	Upregulated	Upregulated
28	CLOCK	Upregulated	Upregulated
29	HOXB4	Upregulated	Upregulated
30	CLPTM1L	Upregulated	Upregulated
31	SUSD2	Upregulated	Upregulated
32	IL6	Upregulated	Upregulated
33	RBBP8	Upregulated	Upregulated
34	SRPK2	Upregulated	Upregulated
35	ADAM9	Upregulated	Upregulated
36	BAG3	Upregulated	Upregulated
37	GSTM4	Upregulated	Upregulated
38	SOD2	Upregulated	Upregulated
39	SLC39A4	Upregulated	Upregulated
40	DVL3	Upregulated	Upregulated
41	SPARC	Upregulated	Upregulated
42	MAP1LC3A	Upregulated	Upregulated
43	HBEGF	Upregulated	Upregulated
44	IKBKB	Upregulated	Upregulated
45	CLU	Upregulated	Upregulated
46	SMO	Upregulated	Upregulated
47	POLD2	Upregulated	Upregulated
48	PIK3CA	Upregulated	Upregulated
49	MAP2K1	Upregulated	Upregulated
50	ATP6V0B	Upregulated	Upregulated
51	LMO4	Upregulated	Upregulated
52	PTPN3	Upregulated	Upregulated
53	CUEDC2	Upregulated	Upregulated
54	GOLPH3	Upregulated	Upregulated
55	HOXA4	Upregulated	Upregulated
56	CCNI	Upregulated	Upregulated
57	RAB18	Upregulated	Upregulated
58	CDK4	Upregulated	Upregulated
59	YWHAG	Upregulated	Upregulated
60	RELB	Upregulated	Upregulated
61	PGD	Upregulated	Upregulated
62	ATP11B	Upregulated	Upregulated
63	ABCC3	Upregulated	Upregulated
64	PRR13	Upregulated	Upregulated
65	XPA	Upregulated	Upregulated
66	DDR1	Upregulated	Upregulated
67	SQSTM1	Upregulated	Upregulated
68	ATP6V1A	Upregulated	Upregulated
69	PXN	Upregulated	Upregulated
70	CUL4A	Upregulated	Upregulated
71	SMARCE1	Upregulated	Upregulated
72	TMEM98	Upregulated	Upregulated
73	HDAC1	Upregulated	Upregulated
74	PDPK1	Upregulated	Upregulated

75	ACTA2	Upregulated	Upregulated
76	CDCP1	Upregulated	Upregulated
77	MAFG	Upregulated	Upregulated
78	PARP1	Upregulated	Upregulated
79	AATF	Upregulated	Upregulated
80	BRD9	Upregulated	Upregulated
81	GAS6	Upregulated	Upregulated
82	YWHAH	Upregulated	Upregulated
83	G6PD	Upregulated	Upregulated
84	ATG5	Upregulated	Upregulated
85	LNPEP	Upregulated	Upregulated
86	EPHA2	Upregulated	Upregulated
87	SLC25A1	Upregulated	Upregulated
88	ATP6V1C1	Upregulated	Upregulated
89	DAXX	Upregulated	Upregulated
90	ATP7A	Upregulated	Upregulated
91	WWTR1	Upregulated	Upregulated
92	GLS2	Upregulated	Upregulated
93	EIF4EBP1	Upregulated	Upregulated
94	DVL2	Upregulated	Upregulated
95	NACC1	Upregulated	Upregulated
96	ARIH1	Upregulated	Upregulated
97	RAC1	Upregulated	Upregulated
98	CFLAR	Upregulated	Upregulated
99	MGAT1	Upregulated	Upregulated
100	GPX3	Upregulated	Upregulated
101	ITGB1	Upregulated	Upregulated
102	SLC31A2	Upregulated	Upregulated
103	NID1	Upregulated	Upregulated
104	GCLM	Upregulated	Upregulated
105	TXNRD1	Upregulated	Upregulated
106	RORC	Upregulated	Upregulated
107	RPS6KB2	Upregulated	Upregulated
108	STAT3	Upregulated	Upregulated
109	HOXD8	Upregulated	Upregulated
110	GPX4	Upregulated	Upregulated
111	XPO1	Upregulated	Upregulated
112	NFE2L2	Upregulated	Upregulated
113	FAT1	Upregulated	Upregulated
114	H6PD	Upregulated	Upregulated
115	YBX1	Upregulated	Upregulated
116	TMEM205	Upregulated	Upregulated
117	PRKAA1	Upregulated	Upregulated
118	UBE2S	Upregulated	Upregulated
119	ITGB5	Upregulated	Upregulated
120	CDK7	Upregulated	Upregulated
121	KLF5	Upregulated	Upregulated
122	NFKB1	Upregulated	Upregulated
123	RHOA	Upregulated	Upregulated
124	ERBB2	Upregulated	Upregulated
125	IL6R	Upregulated	Upregulated
126	RPS6KB1	Upregulated	Upregulated
127	SIRT1	Upregulated	Upregulated
128	AGR3	Upregulated	Upregulated
129	GSTK1	Upregulated	Upregulated

130	ARHGDIB	Upregulated	Upregulated
131	FASN	Upregulated	Upregulated
132	HOXB7	Upregulated	Upregulated
133	GSTP1	Upregulated	Upregulated
134	EIF4E	Upregulated	Upregulated
135	SMARCA4	Upregulated	Upregulated
136	SOX2	Upregulated	Upregulated
137	CYP1B1	Upregulated	Upregulated
138	BSG	Upregulated	Upregulated
139	YAP1	Upregulated	Upregulated
140	URI1	Upregulated	Upregulated
141	ALKBH3	Upregulated	Upregulated
142	NRP2	Upregulated	Upregulated
143	SREBF2	Upregulated	Upregulated
144	UGCG	Upregulated	Upregulated
145	PBXIP1	Upregulated	Upregulated
146	NFKB2	Upregulated	Upregulated
147	NCOA3	Upregulated	Upregulated
148	TAB3	Upregulated	Upregulated
149	RNF2	Upregulated	Upregulated
150	BIRC2	Upregulated	Upregulated
151	STAT5A	Upregulated	Upregulated
152	KAT2B	Upregulated	Upregulated
153	PDK4	Upregulated	Upregulated
154	ANXA3	Upregulated	Upregulated
155	ERBB3	Upregulated	Upregulated
156	HMGCR	Upregulated	Upregulated
157	ATP6V1B2	Upregulated	Upregulated
158	NEDD4	Upregulated	Upregulated
159	ABCC4	Upregulated	Upregulated
160	ATR	Upregulated	Upregulated
161	AREG	Upregulated	Upregulated
162	ABCC5	Upregulated	Upregulated
163	TIMELESS	Upregulated	Upregulated
164	GDF15	Upregulated	Upregulated
165	SLC1A5	Upregulated	Upregulated
166	HEY1	Upregulated	Upregulated
167	ITGB8	Upregulated	Upregulated
168	POLD1	Upregulated	Upregulated
169	USP22	Upregulated	Upregulated
170	GPRC5A	Upregulated	Upregulated
171	GSR	Upregulated	Upregulated
172	AKR1B10	Upregulated	Upregulated
173	TRIM27	Upregulated	Upregulated
174	TBCE	Upregulated	Upregulated
175	SIX1	Upregulated	Upregulated
176	ABCC1	Upregulated	Upregulated
177	MDM2	Upregulated	Upregulated
178	RECQL4	Upregulated	Upregulated
179	LDLR	Upregulated	Upregulated
180	EIF4G2	Upregulated	Upregulated
181	TRIM65	Upregulated	Upregulated
182	ENPP2	Upregulated	Upregulated
183	SLC7A11	Upregulated	Upregulated
184	MUC1	Upregulated	Upregulated

185	FSTL1	Upregulated	Upregulated
186	ENG	Upregulated	Upregulated
187	CAMK2D	Upregulated	Upregulated
188	EZR	Upregulated	Upregulated
189	IKBKE	Upregulated	Upregulated
190	NEDD8	Upregulated	Upregulated
191	MALT1	Upregulated	Upregulated
192	NBN	Upregulated	Upregulated
193	HECTD3	Upregulated	Upregulated
194	MTOR	Upregulated	Upregulated
195	BMPR1A	Upregulated	Upregulated
196	SLC7A5	Upregulated	Upregulated
197	TET1	Upregulated	Upregulated
198	PROM1	Upregulated	Upregulated
199	UIMC1	Upregulated	Upregulated
200	YY1	Upregulated	Upregulated
201	MTDH	Upregulated	Upregulated
202	AMBRA1	Upregulated	Upregulated
203	HSPB1	Upregulated	Upregulated
204	DUSP1	Upregulated	Upregulated
205	CHUK	Upregulated	Upregulated
206	ERCC5	Upregulated	Upregulated
207	PIK3R2	Upregulated	Upregulated
208	PTGS2	Upregulated	Upregulated
209	MAST1	Upregulated	Upregulated
210	IL7	Upregulated	Upregulated
211	FGFR2	Upregulated	Upregulated
212	DCLRE1A	Upregulated	Upregulated
213	AKR1C3	Upregulated	Upregulated
214	TEAD4	Upregulated	Upregulated
215	ATG7	Upregulated	Upregulated
216	COX7A2	Upregulated	Upregulated
217	PTCH1	Upregulated	Upregulated
218	PPM1D	Upregulated	Upregulated
219	FANCC	Upregulated	Upregulated
220	CCL2	Upregulated	Upregulated
221	ROR2	Upregulated	Upregulated
222	CSF1	Upregulated	Upregulated
223	SNAI2	Upregulated	Upregulated
224	EIF2AK3	Upregulated	Upregulated
225	MYD88	Upregulated	Upregulated
226	SIK2	Upregulated	Upregulated
227	PARK7	Upregulated	Upregulated
228	PIK3R1	Upregulated	Upregulated
229	RAD52	Upregulated	Upregulated
230	TP63	Upregulated	Upregulated
231	ABCG2	Upregulated	Upregulated
232	E2F1	Upregulated	Upregulated
233	CLDN7	Upregulated	Upregulated
234	MT1A	Upregulated	Upregulated
235	PPARGC1B	Upregulated	Upregulated
236	ABCA8	Upregulated	Upregulated
237	FZD8	Upregulated	Upregulated

Footnote:

*, 721 Platinum resistance genes are collected in a recent review
Huang D, et al: A highly annotated database of genes associated with platinum resistance in cancer, Oncogene (2021) 40:63[illegible]

[illegible]95 – 6405.
