# Supplementary material for: Non-canonical NOTCH1 signaling regulates ferroptosis vulnerability in dormant lung cancer cells with stable resistance
Source: Cell Death Dis. 2025 Dec 26;17(1):1. doi: 10.1038/s41419-025-08355-9 (PMC12780219; doi:10.1038/s41419-025-08355-9)
Supplement: Supplementary file 6 — Supplementary Table 4 [file 41419_2025_8355_MOESM6_ESM.pdf]

Huang H et. al., Non-canonical NOTCH1 Signaling Regulates Ferroptosis Vulnerability in Dormant Lung Cancer Cells with Stable Resistance

Table S4. Top DEG lists for bulk RNA-seq

Tab 1: A549CR vs A549

This tab is related to Fig. 3J

No.	Gene	Gene function
**Top 10 upregulated genes (A549CR vs. A549)**		
1	CEACAM1	Encodes CD66a that mediates cell adhesion, regulates insulin levels and signaling
2	CLCA2	Encodes calcium-activated chloride channel 2, which transports of chloride across the plasma membrane, CLCA2 is a stress-inducible and p53-inducible gene in response to cell detachment, DNA damage, replicative senescence, oxidative stress, and other stressors. It suppresses EMT.
3	RGMA	A glycosylphosphatidylinositol-anchored glycoprotein and axon guidance molecule that signals through its receptor, neogenin (NEO1). It inhibit cancer cell proliferation.
4	LCE1F	Is a downstream target of p53, and interacts with PRMT5 and inhibits PRMT5-mediated cell cycle progression.
5	CORO2A	A novel component of the N-CoR (nuclear receptor co-repressor) complex, Coro2A was a key regulator of NCoR clearance from target gene promoters. Coro2A is primarily localized to the nucleus.
6	GPR50	GPR50 receptor promotes constitutive TGFβ receptor signaling. It directly interacts with ADAM17 and so activate Notch signaling in a ligand-independent manner through ADAM17
7	PDE7B-AS1	PDE7B-AS1 inhibits expression of PDE7B, a cAMP-specific phosphodiesterase. Phosphodiesterases (PDEs) regulate the intracellular levels of cAMP and cGMP
8	NEFL	NEFL interacts with NEFM to form neurofilaments, which are structural components of the cytoskeleton in mature neurons
9	GRHL3	GRHL3 is a critical activator of PTEN expression. The GRHL3-PTEN axis as a critical tumor suppressor pathway in SCC

10	DGKA	The kinase phosphorylating diacylglycerol to phosphatidic acid. It promotes platinum resistance.

Top 10 downregulated genes (A549CR vs. A549)

1	BUB1	Encodes a mitotic checkpoint serine/threonine kinase in mitosis
2	NUF2	Encodes a component of a conserved protein complex associated with the centromere
3	DLGAP5	Encodes a mitotic microtubule-associated protein that enhances microtubule polymerization and promotes timely formation and stability of a bipolar spindle
4	BUB1B	Encodes a central component of the mitotic checkpoint for spindle assembly (SAC)
5	KIF11	Encodes a molecular motor protein that is required for the formation of the bipolar spindle in metaphase
6	DTL	Encdoes a component of Cul4A-RING E3 ubiquitin ligase complex and Cul4B-RING E3 ubiquitin ligase complex, which is required for the regulation of the G2/M checkpoint. The DCX(DTL) complex mediates the polyubiquitination and subsequent degradation of CDT1, p21(CIP1), FBH1, KMT5A and SDE2
7	MELK	Encodes a mitotic kinase in the AMPK/Snf1 family, which phosphorylates eIF4B and so regulates protein synthesis during mitosis
8	CEP55	Encodes an essential protein required for the completion of cell abscission during mitosis. It is localized to the centrosome in interphase cells and recruited to the midbody during cytokinesis.
9	SPAG5	Encodes a mitotic spindle protein. In normal cells, it ensures the correct separation of sister chromatids into daughter cell.
10	KIF23	Encodes a kinesin-like motor protein, which is involved in the formation of the central spindle and midbody during cytokinesis. It may help to transport organelles within cells and move chromosomes during cell division.

Tab 2: A549CR RO vs. A549CR DMSO

This tab is related to Fig. 5D

No.	Gene	Gene function
**Top 10 upregulated genes (A549CR RO vs. A549CR DMSO)**		
1	CYP24A1	Encodes an enzyme with 24-hydroxylase activity that inactivates vitamin D metabolites, crucial for calcium homeostasis.
2	PMEPA1	A TGF-beta-inducible protein that functions as a negative regulator of TGF-beta signaling, influencing cell proliferation, differentiation, apoptosis, motility, extracellular matrix production, and immunosuppression.
3	SERPINE1	Encodes plasminogen activator inhibitor-1 (PAI-1), a primary inhibitor of tissue plasminogen activator and urokinase (uPA-uPAR interaction in ECM), regulating fibrinolysis. Involved in cellular senescence.
4	SLC2A3	Encodes glucose transporter 3 (GLUT3), facilitating glucose uptake in neurons and other tissues.
5	CCN2	Encodes connective tissue growth factor (CTGF), involved in cell adhesion, migration, proliferation, and differentiation.
6	MMP7	Encodes matrix metalloproteinase-7, involved in the breakdown of extracellular matrix components.
7	HMOX1	Encodes heme oxygenase 1, which degrades heme into biliverdin, ferrous ions, and carbon monoxide. It is involved in ferroptosis
8	TUBA4A	Encodes tubulin alpha-4A, a component of microtubules essential for cell structure and division.
9	TGFBI	Encodes a TGFβ-induced secreting protein, involved in cell adhesion and extracellular matrix interactions.
10	SOX4	Encodes a transcription factor that regulates EMT

Top 10 downregulated genes (A549CR RO vs. A549CR DMSO)

1	MUC5B	Encodes mucin 5B, a gel-forming glycoprotein that contributes to the viscoelastic properties of mucus, playing a crucial role in mucosal defense.
2	CYP4F3	Encodes cytochrome P450 family 4 subfamily F member 3, which catalyzes the ω-oxidation of fatty acids and vitamin E. It is the closest analog to CYP4F2, which is know to be involved in the ω-oxidation of vitamin K
3	AGR2	Encodes anterior gradient protein 2, a disulfide isomerase involved in protein folding within the endoplasmic reticulum. AGR2 maintains epithelial phenotypes.
4	CYP4F11	Encodes cytochrome P450 family 4 subfamily F member 11, involved in the ω-oxidation of fatty acids, vitamin K, and vitamin E.
5	CP	Encodes ceruloplasmin, a ferroxidase enzyme involved in iron metabolism, antioxidant defense, and inhibition of erastin- and RSL3-induced ferroptosis
6	CYP1B1	Encodes cytochrome P450 family 1 subfamily B member 1, involved in the metabolism of xenobiotics and endogenous substrates. CYP1B1 knockout leads to iron accumulation.
7	ITGB8	Encodes integrin beta-8, a protein involved in cell adhesion and signaling. It also contributes to activation of latent TGFB1.
8	CYP4F12	Encodes cytochrome P450 family 4 subfamily F member 12, involved in the catabolism of fatty acids and vitamin E.
9	DHCR24	Encodes 24-dehydrocholesterol reductase, an enzyme involved in cholesterol and vitamin D biosynthesis.
10	LPIN1	Encodes lipin 1, a phosphatidate phosphatase involved in lipid metabolism.
