# Supplementary material for: Non-canonical NOTCH1 signaling regulates ferroptosis vulnerability in dormant lung cancer cells with stable resistance
Source: Cell Death Dis. 2025 Dec 26;17(1):1. doi: 10.1038/s41419-025-08355-9 (PMC12780219; doi:10.1038/s41419-025-08355-9)
Supplement: Supplementary file 7 — Supplementary Table 5 [file 41419_2025_8355_MOESM7_ESM.pdf]

Huang H et. al., Non-canonical NOTCH1 Signaling Regulates Ferroptosis Vulnerability in Dormant Lung Cancer Cells with Stable Resistance

Table S5. ***SLC52A1-3*** **expression in the chemotherapy-treated TCGA-LUAD patients**

Related to Fig. 3M

ID (barcode)	bcr_patient_barcode	Sample ID	Disease	Tissue_or_organ_of_origin	Gender	Age_at_index	Project_id	Therapy_types	Drug_name	SLC52A1 (TPM)	SLC52A2 (TPM)	SLC52A3 (TPM)
TCGA-05-4390-01A-02R-1755-07	TCGA-05-4390-01A	TCGA-05-4390-01A	Lung Adenocarcinoma	Upper lobe, lung	female	58	TCGA-LUAD	Chemotherapy	Cisplatin	2.6268	118.5182	0.1466
TCGA-05-4398-01A-01R-1206-07	TCGA-05-4398-01A	TCGA-05-4398-01A	Lung Adenocarcinoma	Upper lobe, lung	female	47	TCGA-LUAD	Chemotherapy	Carboplatin	1.2505	110.7557	8.5387
TCGA-05-4402-01A-01R-1206-07	TCGA-05-4402-01A	TCGA-05-4402-01A	Lung Adenocarcinoma	Lower lobe, lung	female	57	TCGA-LUAD	Chemotherapy	Carboplatin	2.7516	95.8194	11.6185
TCGA-05-4425-01A-01R-1755-07	TCGA-05-4425-01A	TCGA-05-4425-01A	Lung Adenocarcinoma	Middle lobe, lung	female	70	TCGA-LUAD	Chemotherapy	Cisplatin	6.3925	63.975	11.9299
TCGA-05-4427-01A-21R-1858-07	TCGA-05-4427-01A	TCGA-05-4427-01A	Lung Adenocarcinoma	Upper lobe, lung	female	65	TCGA-LUAD	Chemotherapy	Cisplatin	0.74	51.2169	0.3355
TCGA-05-4432-01A-01R-1206-07	TCGA-05-4432-01A	TCGA-05-4432-01A	Lung Adenocarcinoma	Upper lobe, lung	male	66	TCGA-LUAD	Chemotherapy	Cisplatin	2.2477	71.8425	4.9656
TCGA-05-5425-01A-02R-1628-07	TCGA-05-5425-01A	TCGA-05-5425-01A	Lung Adenocarcinoma	Lung, NOS	male	68	TCGA-LUAD	Chemotherapy	Carboplatin	2.7346	60.2129	6.5606
TCGA-05-5428-01A-01R-1628-07	TCGA-05-5428-01A	TCGA-05-5428-01A	Lung Adenocarcinoma	Lung, NOS	male	57	TCGA-LUAD	Chemotherapy	Cisplatin	0.8848	54.232	1.9944
TCGA-38-4628-01A-01R-1206-07	TCGA-38-4628-01A	TCGA-38-4628-01A	Lung Adenocarcinoma	Upper lobe, lung	female	65	TCGA-LUAD	Chemotherapy	Carboplatin	20.6285	77.8812	7.8469
TCGA-38-4632-01A-01R-1755-07	TCGA-38-4632-01A	TCGA-38-4632-01A	Lung Adenocarcinoma	Upper lobe, lung	male	42	TCGA-LUAD	Chemotherapy	CPT-11	1.654	148.5015	1.8122
TCGA-38-6178-01A-11R-1755-07	TCGA-38-6178-01A	TCGA-38-6178-01A	Lung Adenocarcinoma	Upper lobe, lung	female	70	TCGA-LUAD	Chemotherapy	Carboplatin	7.029	147.9293	9.7841
TCGA-38-7271-01A-11R-2039-07	TCGA-38-7271-01A	TCGA-38-7271-01A	Lung Adenocarcinoma	Upper lobe, lung	female	72	TCGA-LUAD	Chemotherapy	Alimta	0.5097	34.2631	4.0821
TCGA-44-2659-01A-01R-0946-07	TCGA-44-2659-01A	TCGA-44-2659-01A	Lung Adenocarcinoma	Lower lobe, lung	female	65	TCGA-LUAD	Chemotherapy	Alimta	2.4866	33.2418	3.7046
TCGA-44-2665-01A-01R-0946-07	TCGA-44-2665-01A	TCGA-44-2665-01A	Lung Adenocarcinoma	Upper lobe, lung	female	55	TCGA-LUAD	Chemotherapy	Cisplatin	1.9191	50.5367	4.8364
TCGA-44-2665-01A-01R-A278-07	TCGA-44-2665-01A	TCGA-44-2665-01A	Lung Adenocarcinoma	Upper lobe, lung	female	55	TCGA-LUAD	Chemotherapy	Cisplatin	0.9617	18.0402	1.2374
TCGA-44-2665-01B-06R-A277-07	TCGA-44-2665-01B	TCGA-44-2665-01B	Lung Adenocarcinoma	Upper lobe, lung	female	55	TCGA-LUAD	Chemotherapy	Cisplatin	1.1383	4.1561	1.3606
TCGA-44-3396-01A-01R-1206-07	TCGA-44-3396-01A	TCGA-44-3396-01A	Lung Adenocarcinoma	Upper lobe, lung	female	74	TCGA-LUAD	Chemotherapy	Alimta	0.243	66.8162	5.7966
TCGA-44-3918-01A-01R-1107-07	TCGA-44-3918-01A	TCGA-44-3918-01A	Lung Adenocarcinoma	Upper lobe, lung	female	60	TCGA-LUAD	Chemotherapy	Alimta	1.9454	52.7176	0.3215
TCGA-44-3918-01A-01R-A278-07	TCGA-44-3918-01A	TCGA-44-3918-01A	Lung Adenocarcinoma	Upper lobe, lung	female	60	TCGA-LUAD	Chemotherapy	Alimta	0.4667	5.8984	0.1395
TCGA-44-3918-01B-02R-A277-07	TCGA-44-3918-01B	TCGA-44-3918-01B	Lung Adenocarcinoma	Upper lobe, lung	female	60	TCGA-LUAD	Chemotherapy	Alimta	2.1812	10.1081	0.2817
TCGA-44-4112-01A-01R-1107-07	TCGA-44-4112-01A	TCGA-44-4112-01A	Lung Adenocarcinoma	Upper lobe, lung	female	60	TCGA-LUAD	Chemotherapy	Alimta	2.7922	34.073	5.0825
TCGA-44-4112-01A-01R-A278-07	TCGA-44-4112-01A	TCGA-44-4112-01A	Lung Adenocarcinoma	Upper lobe, lung	female	60	TCGA-LUAD	Chemotherapy	Alimta	0.7534	9.1333	1.0684
TCGA-44-4112-01B-06R-A277-07	TCGA-44-4112-01B	TCGA-44-4112-01B	Lung Adenocarcinoma	Upper lobe, lung	female	60	TCGA-LUAD	Chemotherapy	Alimta	1.9317	4.3434	1.5662
TCGA-44-5643-01A-01R-1628-07	TCGA-44-5643-01A	TCGA-44-5643-01A	Lung Adenocarcinoma	Lower lobe, lung	male	53	TCGA-LUAD	Chemotherapy	Carboplatin	6.2457	41.8624	5.814
TCGA-44-6146-01A-11R-1755-07	TCGA-44-6146-01A	TCGA-44-6146-01A	Lung Adenocarcinoma	Lower lobe, lung	male	64	TCGA-LUAD	Chemotherapy	Alimta	0.1339	67.7248	16.9212
TCGA-44-6146-01A-11R-A278-07	TCGA-44-6146-01A	TCGA-44-6146-01A	Lung Adenocarcinoma	Lower lobe, lung	male	64	TCGA-LUAD	Chemotherapy	Alimta	0.144	34.2187	8.0495
TCGA-44-6146-01B-04R-A277-07	TCGA-44-6146-01B	TCGA-44-6146-01B	Lung Adenocarcinoma	Lower lobe, lung	male	64	TCGA-LUAD	Chemotherapy	Alimta	0.6163	4.4854	4.8622
TCGA-44-6774-01A-21R-1858-07	TCGA-44-6774-01A	TCGA-44-6774-01A	Lung Adenocarcinoma	Lower lobe, lung	female	56	TCGA-LUAD	Chemotherapy	Alimta	1.1469	63.4118	4.1234
TCGA-44-6779-01A-11R-1858-07	TCGA-44-6779-01A	TCGA-44-6779-01A	Lung Adenocarcinoma	Lung, NOS	female	50	TCGA-LUAD	Chemotherapy	Carboplatin	0.0699	61.0397	8.955
TCGA-44-7669-01A-21R-2066-07	TCGA-44-7669-01A	TCGA-44-7669-01A	Lung Adenocarcinoma	Upper lobe, lung	male	59	TCGA-LUAD	Chemotherapy	Alimta	0.2406	47.1203	0.6821
TCGA-44-7670-01A-11R-2066-07	TCGA-44-7670-01A	TCGA-44-7670-01A	Lung Adenocarcinoma	Upper lobe, lung	female	47	TCGA-LUAD	Chemotherapy	Cisplatin	4.593	73.6406	13.9978
TCGA-44-8117-01A-11R-2241-07	TCGA-44-8117-01A	TCGA-44-8117-01A	Lung Adenocarcinoma	Upper lobe, lung	female	54	TCGA-LUAD	Chemotherapy	Cisplatin	2.3287	74.4789	0.2905
TCGA-49-4490-01A-21R-1858-07	TCGA-49-4490-01A	TCGA-49-4490-01A	Lung Adenocarcinoma	Upper lobe, lung	female	45	TCGA-LUAD	Chemotherapy	Cisplatin	6.1257	128.621	12.6876
TCGA-49-4501-01A-01R-1206-07	TCGA-49-4501-01A	TCGA-49-4501-01A	Lung Adenocarcinoma	Upper lobe, lung	female	67	TCGA-LUAD	Chemotherapy	Gemzar	4.4462	60.3791	6.1123
TCGA-49-4507-01A-01R-1206-07	TCGA-49-4507-01A	TCGA-49-4507-01A	Lung Adenocarcinoma	Main bronchus	female	73	TCGA-LUAD	Chemotherapy	Carboplatin	1.4366	127.4709	5.3155
TCGA-49-4512-01A-21R-1858-07	TCGA-49-4512-01A	TCGA-49-4512-01A	Lung Adenocarcinoma	Middle lobe, lung	female	69	TCGA-LUAD	Chemotherapy	NA	2.2113	76.2166	1.9252
TCGA-49-6742-01A-11R-1858-07	TCGA-49-6742-01A	TCGA-49-6742-01A	Lung Adenocarcinoma	Upper lobe, lung	male	70	TCGA-LUAD	Chemotherapy	Alimta	7.8373	48.7594	11.169
TCGA-49-6743-01A-11R-1858-07	TCGA-49-6743-01A	TCGA-49-6743-01A	Lung Adenocarcinoma	Upper lobe, lung	female	81	TCGA-LUAD	Chemotherapy	Bevacizumab	7.6659	40.0189	9.3682
TCGA-49-6744-01A-11R-1858-07	TCGA-49-6744-01A	TCGA-49-6744-01A	Lung Adenocarcinoma	Upper lobe, lung	female	64	TCGA-LUAD	Chemotherapy	NA	2.8867	32.3391	7.4292
TCGA-49-6745-01A-11R-1858-07	TCGA-49-6745-01A	TCGA-49-6745-01A	Lung Adenocarcinoma	Upper lobe, lung	male	82	TCGA-LUAD	Chemotherapy	NA	1.6057	49.9245	4.9055
TCGA-49-AAR2-01A-11R-A39D-07	TCGA-49-AAR2-01A	TCGA-49-AAR2-01A	Lung Adenocarcinoma	Lower lobe, lung	male	64	TCGA-LUAD	Chemotherapy	TAXOL	10.2162	71.0816	4.4527
TCGA-49-AAR4-01A-12R-A41B-07	TCGA-49-AAR4-01A	TCGA-49-AAR4-01A	Lung Adenocarcinoma	Upper lobe, lung	male	51	TCGA-LUAD	Chemotherapy	CARBOPLATIN	2.2885	47.2077	4.3297
TCGA-50-5051-01A-21R-1858-07	TCGA-50-5051-01A	TCGA-50-5051-01A	Lung Adenocarcinoma	Upper lobe, lung	female	42	TCGA-LUAD	Chemotherapy	Carboplatin	0.3639	86.5105	1.4918
TCGA-50-5066-01A-01R-1628-07	TCGA-50-5066-01A	TCGA-50-5066-01A	Lung Adenocarcinoma	Upper lobe, lung	male	72	TCGA-LUAD	Chemotherapy	Avastin	0.7997	25.2823	0.8314
TCGA-50-5068-01A-01R-1628-07	TCGA-50-5068-01A	TCGA-50-5068-01A	Lung Adenocarcinoma	Lower lobe, lung	female	59	TCGA-LUAD	Chemotherapy	Taxol	1.2751	30.8455	2.7613
TCGA-50-5072-01A-21R-1858-07	TCGA-50-5072-01A	TCGA-50-5072-01A	Lung Adenocarcinoma	Upper lobe, lung	male	74	TCGA-LUAD	Chemotherapy	Carboplatin	3.1044	80.8001	13.0523
TCGA-50-5930-01A-11R-1755-07	TCGA-50-5930-01A	TCGA-50-5930-01A	Lung Adenocarcinoma	Lower lobe, lung	male	47	TCGA-LUAD	Chemotherapy	Taxotere	6.3347	36.7004	8.0619
TCGA-50-5933-01A-11R-1755-07	TCGA-50-5933-01A	TCGA-50-5933-01A	Lung Adenocarcinoma	Lower lobe, lung	male	72	TCGA-LUAD	Chemotherapy	Carboplatin	3.5597	118.2858	2.0203
TCGA-50-5936-01A-11R-1628-07	TCGA-50-5936-01A	TCGA-50-5936-01A	Lung Adenocarcinoma	Lower lobe, lung	male	58	TCGA-LUAD	Chemotherapy	TAXOL	0.398	48.4983	10.3781
TCGA-50-5941-01A-11R-1755-07	TCGA-50-5941-01A	TCGA-50-5941-01A	Lung Adenocarcinoma	Upper lobe, lung	female	55	TCGA-LUAD	Chemotherapy	Cisplatin	2.2299	92.6077	7.6694
TCGA-50-6590-01A-12R-1858-07	TCGA-50-6590-01A	TCGA-50-6590-01A	Lung Adenocarcinoma	Upper lobe, lung	female	72	TCGA-LUAD	Chemotherapy	Carboplatin	0.6383	61.8931	6.1353
TCGA-50-6595-01A-12R-1858-07	TCGA-50-6595-01A	TCGA-50-6595-01A	Lung Adenocarcinoma	Upper lobe, lung	female	74	TCGA-LUAD	Chemotherapy	Carboplatin	0.7513	66.8632	9.9728
TCGA-50-8459-01A-11R-2326-07	TCGA-50-8459-01A	TCGA-50-8459-01A	Lung Adenocarcinoma	Lower lobe, lung	male	68	TCGA-LUAD	Chemotherapy	ALIMTA	1.0411	39.2246	2.2587
TCGA-53-7624-01A-11R-2066-07	TCGA-53-7624-01A	TCGA-53-7624-01A	Lung Adenocarcinoma	Upper lobe, lung	female	40	TCGA-LUAD	Chemotherapy	Abraxane	7.1772	51.129	17.1617
TCGA-53-7626-01A-12R-2066-07	TCGA-53-7626-01A	TCGA-53-7626-01A	Lung Adenocarcinoma	Upper lobe, lung	female	76	TCGA-LUAD	Chemotherapy	Cisplatin	4.0008	39.5488	5.0539
TCGA-53-7813-01A-11R-2170-07	TCGA-53-7813-01A	TCGA-53-7813-01A	Lung Adenocarcinoma	Upper lobe, lung	female	51	TCGA-LUAD	Chemotherapy	Cisplatin	0.371	23.8527	0.8078
TCGA-53-A4EZ-01A-12R-A24X-07	TCGA-53-A4EZ-01A	TCGA-53-A4EZ-01A	Lung Adenocarcinoma	Upper lobe, lung	male	63	TCGA-LUAD	Chemotherapy	Cisplatin	0.8469	126.7684	17.7673
TCGA-55-1596-01A-01R-0946-07	TCGA-55-1596-01A	TCGA-55-1596-01A	Lung Adenocarcinoma	Upper lobe, lung	male	55	TCGA-LUAD	Chemotherapy	Carboplatin	8.4585	133.3241	24.6468
TCGA-55-5899-01A-11R-1628-07	TCGA-55-5899-01A	TCGA-55-5899-01A	Lung Adenocarcinoma	Upper lobe, lung	male	58	TCGA-LUAD	Chemotherapy	Carboplatin	0.2146	78.6682	2.3518
TCGA-55-6712-01A-11R-1858-07	TCGA-55-6712-01A	TCGA-55-6712-01A	Lung Adenocarcinoma	Upper lobe, lung	male	71	TCGA-LUAD	Chemotherapy	Carboplatin	0.4948	55.6931	5.3387
TCGA-55-6968-01A-11R-1949-07	TCGA-55-6968-01A	TCGA-55-6968-01A	Lung Adenocarcinoma	Lower lobe, lung	male	61	TCGA-LUAD	Chemotherapy	NA	0.4102	49.8075	0.4045
TCGA-55-6970-01A-11R-1949-07	TCGA-55-6970-01A	TCGA-55-6970-01A	Lung Adenocarcinoma	Upper lobe, lung	female	67	TCGA-LUAD	Chemotherapy	Cisplatin	1.288	87.131	6.0372
TCGA-55-6979-01A-11R-1949-07	TCGA-55-6979-01A	TCGA-55-6979-01A	Lung Adenocarcinoma	Upper lobe, lung	female	59	TCGA-LUAD	Chemotherapy	Carboplatin	1.3208	41.9282	4.2959
TCGA-55-6981-01A-11R-1949-07	TCGA-55-6981-01A	TCGA-55-6981-01A	Lung Adenocarcinoma	Upper lobe, lung	female	53	TCGA-LUAD	Chemotherapy	Chemo, NOS	2.0959	77.0271	7.8971
TCGA-55-6982-01A-11R-1949-07	TCGA-55-6982-01A	TCGA-55-6982-01A	Lung Adenocarcinoma	Lower lobe, lung	female	79	TCGA-LUAD	Chemotherapy	Gemzar	1.0583	90.0567	7.5738
TCGA-55-6983-01A-11R-1949-07	TCGA-55-6983-01A	TCGA-55-6983-01A	Lung Adenocarcinoma	Lower lobe, lung	male	81	TCGA-LUAD	Chemotherapy	Chemo, NOS	1.5336	51.6917	6.7693
TCGA-55-6984-01A-11R-1949-07	TCGA-55-6984-01A	TCGA-55-6984-01A	Lung Adenocarcinoma	Lower lobe, lung	female	71	TCGA-LUAD	Chemotherapy	Chemo, Multi-Age	0.2356	51.1791	6.3359
TCGA-55-7227-01A-11R-2039-07	TCGA-55-7227-01A	TCGA-55-7227-01A	Lung Adenocarcinoma	Lower lobe, lung	male	77	TCGA-LUAD	Chemotherapy	Carboplatin	0.4736	43.0628	4.033
TCGA-55-7281-01A-11R-2039-07	TCGA-55-7281-01A	TCGA-55-7281-01A	Lung Adenocarcinoma	Middle lobe, lung	female	70	TCGA-LUAD	Chemotherapy	Alimta	1.4927	51.4869	7.911
TCGA-55-7283-01A-11R-2039-07	TCGA-55-7283-01A	TCGA-55-7283-01A	Lung Adenocarcinoma	Upper lobe, lung	female	76	TCGA-LUAD	Chemotherapy	Alimta	7.4548	69.4181	10.2
TCGA-55-7574-01A-11R-2039-07	TCGA-55-7574-01A	TCGA-55-7574-01A	Lung Adenocarcinoma	Upper lobe, lung	female	64	TCGA-LUAD	Chemotherapy	Avastin	0.6509	47.7408	6.9027

TCGA-55-7576-01A-11R-2066-07	TCGA-55-7576-01A	TCGA-55-7576-01A	Lung Adenocarcinoma	Upper lobe, lung	male	54	TCGA-LUAD	Chemotherapy	Alimta	3.1345	58.167	5.2401
TCGA-55-7815-01A-11R-2170-07	TCGA-55-7815-01A	TCGA-55-7815-01A	Lung Adenocarcinoma	Lower lobe, lung	male	76	TCGA-LUAD	Chemotherapy	Chemo, NOS	0.4985	3.9097	2.1933
TCGA-55-7910-01A-11R-2170-07	TCGA-55-7910-01A	TCGA-55-7910-01A	Lung Adenocarcinoma	Lower lobe, lung	female	50	TCGA-LUAD	Chemotherapy	Cisplatin	0.9052	34.6577	2.538
TCGA-55-7914-01A-11R-2170-07	TCGA-55-7914-01A	TCGA-55-7914-01A	Lung Adenocarcinoma	Upper lobe, lung	female	71	TCGA-LUAD	Chemotherapy	Alimta	0.8779	34.1875	9.4872
TCGA-55-7994-01A-11R-2187-07	TCGA-55-7994-01A	TCGA-55-7994-01A	Lung Adenocarcinoma	Upper lobe, lung	male	81	TCGA-LUAD	Chemotherapy	Carboplatin	0.4141	92.3934	8.9142
TCGA-55-7995-01A-11R-2187-07	TCGA-55-7995-01A	TCGA-55-7995-01A	Lung Adenocarcinoma	Upper lobe, lung	female	73	TCGA-LUAD	Chemotherapy	Carboplatin	1.3105	29.7174	10.3055
TCGA-55-8205-01A-11R-2241-07	TCGA-55-8205-01A	TCGA-55-8205-01A	Lung Adenocarcinoma	Lower lobe, lung	female	76	TCGA-LUAD	Chemotherapy	Alimta	0.3174	68.1547	3.8649
TCGA-55-8301-01A-11R-2287-07	TCGA-55-8301-01A	TCGA-55-8301-01A	Lung Adenocarcinoma	Lower lobe, lung	male	58	TCGA-LUAD	Chemotherapy	Carboplatin	0.6319	67.3071	0.4554
TCGA-55-8505-01A-11R-2403-07	TCGA-55-8505-01A	TCGA-55-8505-01A	Lung Adenocarcinoma	Lower lobe, lung	male	62	TCGA-LUAD	Chemotherapy	Alimta	2.2068	61.834	9.5055
TCGA-55-8508-01A-11R-2403-07	TCGA-55-8508-01A	TCGA-55-8508-01A	Lung Adenocarcinoma	Upper lobe, lung	female	60	TCGA-LUAD	Chemotherapy	Cisplatin	0.4449	42.3876	1.8311
TCGA-55-8513-01A-11R-2403-07	TCGA-55-8513-01A	TCGA-55-8513-01A	Lung Adenocarcinoma	Lower lobe, lung	female	77	TCGA-LUAD	Chemotherapy	Alimta	2.9931	33.6162	5.699
TCGA-55-8615-01A-11R-2403-07	TCGA-55-8615-01A	TCGA-55-8615-01A	Lung Adenocarcinoma	Middle lobe, lung	male	67	TCGA-LUAD	Chemotherapy	Alimta	4.7723	81.9567	5.5027
TCGA-55-A48X-01A-11R-A24H-07	TCGA-55-A48X-01A	TCGA-55-A48X-01A	Lung Adenocarcinoma	Lower lobe, lung	female	63	TCGA-LUAD	Chemotherapy	Alimta	3.4369	55.888	9.2435
TCGA-55-A48Y-01A-11R-A24H-07	TCGA-55-A48Y-01A	TCGA-55-A48Y-01A	Lung Adenocarcinoma	Lower lobe, lung	male	69	TCGA-LUAD	Chemotherapy	Alimta	0.3369	66.8377	7.2448
TCGA-55-A48Z-01A-12R-A24X-07	TCGA-55-A48Z-01A	TCGA-55-A48Z-01A	Lung Adenocarcinoma	Upper lobe, lung	female	60	TCGA-LUAD	Chemotherapy	Alimta	0.48	74.4995	3.1243
TCGA-55-A490-01A-11R-A466-07	TCGA-55-A490-01A	TCGA-55-A490-01A	Lung Adenocarcinoma	Upper lobe, lung	male	78	TCGA-LUAD	Chemotherapy	Alimta	3.0965	51.9216	2.0054
TCGA-62-8394-01A-11R-2326-07	TCGA-62-8394-01A	TCGA-62-8394-01A	Lung Adenocarcinoma	Lower lobe, lung	female	65	TCGA-LUAD	Chemotherapy	Carboplatin	6.5976	162.1981	12.0637
TCGA-62-8398-01A-11R-2326-07	TCGA-62-8398-01A	TCGA-62-8398-01A	Lung Adenocarcinoma	Lower lobe, lung	male	55	TCGA-LUAD	Chemotherapy	Carboplatin	1.3062	88.0328	28.7509
TCGA-62-A46U-01A-11R-A24H-07	TCGA-62-A46U-01A	TCGA-62-A46U-01A	Lung Adenocarcinoma	Lower lobe, lung	female	71	TCGA-LUAD	Chemotherapy	Carboplatin	4.0668	38.7504	0.2698
TCGA-62-A46Y-01A-11R-A24H-07	TCGA-62-A46Y-01A	TCGA-62-A46Y-01A	Lung Adenocarcinoma	Lower lobe, lung	female	70	TCGA-LUAD	Chemotherapy	Cisplatin	2.156	45.7801	0.5525
TCGA-62-A471-01A-12R-A24H-07	TCGA-62-A471-01A	TCGA-62-A471-01A	Lung Adenocarcinoma	Upper lobe, lung	male	64	TCGA-LUAD	Chemotherapy	Cisplatin	6.4146	105.696	28.1374
TCGA-64-1677-01A-01R-0946-07	TCGA-64-1677-01A	TCGA-64-1677-01A	Lung Adenocarcinoma	Lower lobe, lung	female	77	TCGA-LUAD	Chemotherapy	NA	0.9943	54.3308	2.3263
TCGA-64-1678-01A-01R-0946-07	TCGA-64-1678-01A	TCGA-64-1678-01A	Lung Adenocarcinoma	Middle lobe, lung	female	70	TCGA-LUAD	Chemotherapy	Carboplatin	0.1083	31.0813	0.0712
TCGA-64-1679-01A-21R-2066-07	TCGA-64-1679-01A	TCGA-64-1679-01A	Lung Adenocarcinoma	Upper lobe, lung	female	58	TCGA-LUAD	Chemotherapy	Cisplatin	3.3104	43.9251	7.8315
TCGA-64-1680-01A-02R-0946-07	TCGA-64-1680-01A	TCGA-64-1680-01A	Lung Adenocarcinoma	Overlapping lesion of lung	male	63	TCGA-LUAD	Chemotherapy	NA	0.7099	46.8461	7.9516
TCGA-64-1681-01A-11R-2066-07	TCGA-64-1681-01A	TCGA-64-1681-01A	Lung Adenocarcinoma	Lung, NOS	female	61	TCGA-LUAD	Chemotherapy	Tarceva	5.4381	43.8399	3.627
TCGA-64-5775-01A-01R-1628-07	TCGA-64-5775-01A	TCGA-64-5775-01A	Lung Adenocarcinoma	Upper lobe, lung	male	71	TCGA-LUAD	Chemotherapy	Carboplatin	0.0441	109.3005	1.0447
TCGA-64-5778-01A-01R-1628-07	TCGA-64-5778-01A	TCGA-64-5778-01A	Lung Adenocarcinoma	Upper lobe, lung	male	60	TCGA-LUAD	Chemotherapy	Tarceva	1.1288	50.1788	4.9584
TCGA-64-5779-01A-01R-1628-07	TCGA-64-5779-01A	TCGA-64-5779-01A	Lung Adenocarcinoma	Lower lobe, lung	male	61	TCGA-LUAD	Chemotherapy	Cisplatin	0.4488	43.0372	0.4917
TCGA-64-5781-01A-01R-1628-07	TCGA-64-5781-01A	TCGA-64-5781-01A	Lung Adenocarcinoma	Upper lobe, lung	female	55	TCGA-LUAD	Chemotherapy	Bevacizumab	0.9749	61.7172	4.4343
TCGA-64-5815-01A-01R-1628-07	TCGA-64-5815-01A	TCGA-64-5815-01A	Lung Adenocarcinoma	Upper lobe, lung	male	74	TCGA-LUAD	Chemotherapy	Alimta	1.2419	41.81	4.335
TCGA-67-6215-01A-11R-1755-07	TCGA-67-6215-01A	TCGA-67-6215-01A	Lung Adenocarcinoma	Lower lobe, lung	female	52	TCGA-LUAD	Chemotherapy	ALIMTA	1.0518	58.2664	6.8907
TCGA-67-6217-01A-11R-1755-07	TCGA-67-6217-01A	TCGA-67-6217-01A	Lung Adenocarcinoma	Upper lobe, lung	female	73	TCGA-LUAD	Chemotherapy	Tarceva	3.7127	30.8787	4.5676
TCGA-69-7760-01A-11R-2170-07	TCGA-69-7760-01A	TCGA-69-7760-01A	Lung Adenocarcinoma	Lower lobe, lung	male	73	TCGA-LUAD	Chemotherapy	Carboplatin	3.6198	57.5192	7.053
TCGA-69-7761-01A-11R-2170-07	TCGA-69-7761-01A	TCGA-69-7761-01A	Lung Adenocarcinoma	Lower lobe, lung	male	84	TCGA-LUAD	Chemotherapy	Alimta	0.5966	29.3844	2.5212
TCGA-69-7765-01A-11R-2170-07	TCGA-69-7765-01A	TCGA-69-7765-01A	Lung Adenocarcinoma	Upper lobe, lung	male	56	TCGA-LUAD	Chemotherapy	Carboplatin	0.599	30.4838	8.0936
TCGA-69-7973-01A-11R-2187-07	TCGA-69-7973-01A	TCGA-69-7973-01A	Lung Adenocarcinoma	Middle lobe, lung	female	42	TCGA-LUAD	Chemotherapy	Cisplatin	1.7574	69.7576	4.5628
TCGA-69-7974-01A-11R-2187-07	TCGA-69-7974-01A	TCGA-69-7974-01A	Lung Adenocarcinoma	Upper lobe, lung	female	54	TCGA-LUAD	Chemotherapy	Carboplatin	0.7154	63.0907	8.3355
TCGA-69-8253-01A-11R-2287-07	TCGA-69-8253-01A	TCGA-69-8253-01A	Lung Adenocarcinoma	Lower lobe, lung	female	59	TCGA-LUAD	Chemotherapy	Cisplatin	0.8323	53.9624	12.4519
TCGA-69-8453-01A-12R-2326-07	TCGA-69-8453-01A	TCGA-69-8453-01A	Lung Adenocarcinoma	Lower lobe, lung	male	77	TCGA-LUAD	Chemotherapy	Alimta	1.4903	47.667	4.7603
TCGA-69-A59K-01A-11R-A262-07	TCGA-69-A59K-01A	TCGA-69-A59K-01A	Lung Adenocarcinoma	Upper lobe, lung	female	60	TCGA-LUAD	Chemotherapy	cisplatin	2.5	41.2715	3.9384
TCGA-71-6725-01A-11R-1858-07	TCGA-71-6725-01A	TCGA-71-6725-01A	Lung Adenocarcinoma	Upper lobe, lung	female	48	TCGA-LUAD	Chemotherapy	Cisplatin	4.7744	106.4705	11.6876
TCGA-73-4666-01A-01R-1206-07	TCGA-73-4666-01A	TCGA-73-4666-01A	Lung Adenocarcinoma	Upper lobe, lung	female	52	TCGA-LUAD	Chemotherapy	carboplatin	0.6845	59.2583	6.4237
TCGA-73-4668-01A-01R-1206-07	TCGA-73-4668-01A	TCGA-73-4668-01A	Lung Adenocarcinoma	Upper lobe, lung	female	66	TCGA-LUAD	Chemotherapy	Carboplatin	3.8842	49.6139	2.233
TCGA-73-4670-01A-01R-1206-07	TCGA-73-4670-01A	TCGA-73-4670-01A	Lung Adenocarcinoma	Upper lobe, lung	female	69	TCGA-LUAD	Chemotherapy	Avastin	0.2904	108.2839	4.5228
TCGA-73-4675-01A-01R-1206-07	TCGA-73-4675-01A	TCGA-73-4675-01A	Lung Adenocarcinoma	Lower lobe, lung	male	59	TCGA-LUAD	Chemotherapy	Alimta	0.2877	64.0965	12.1869
TCGA-73-4676-01A-01R-1755-07	TCGA-73-4676-01A	TCGA-73-4676-01A	Lung Adenocarcinoma	Upper lobe, lung	male	45	TCGA-LUAD	Chemotherapy	Cisplatin	4.4008	82.1641	4.0283
TCGA-73-7498-01A-12R-2187-07	TCGA-73-7498-01A	TCGA-73-7498-01A	Lung Adenocarcinoma	Lower lobe, lung	female	58	TCGA-LUAD	Chemotherapy	Carboplatin	1.3053	67.9099	6.8351
TCGA-73-A9RS-01A-11R-A41B-07	TCGA-73-A9RS-01A	TCGA-73-A9RS-01A	Lung Adenocarcinoma	Upper lobe, lung	male	41	TCGA-LUAD	Chemotherapy	carboplatin	0.3473	63.8019	6.5751
TCGA-75-6212-01A-11R-1755-07	TCGA-75-6212-01A	TCGA-75-6212-01A	Lung Adenocarcinoma	Lower lobe, lung	female	NA	TCGA-LUAD	Chemotherapy	Carboplatin	4.619	31.1211	2.7964
TCGA-75-7030-01A-11R-1949-07	TCGA-75-7030-01A	TCGA-75-7030-01A	Lung Adenocarcinoma	Lower lobe, lung	male	NA	TCGA-LUAD	Chemotherapy	Cisplatin	1.0668	30.7615	4.2717
TCGA-78-7150-01A-21R-2039-07	TCGA-78-7150-01A	TCGA-78-7150-01A	Lung Adenocarcinoma	Lower lobe, lung	male	59	TCGA-LUAD	Chemotherapy	Carboplatin	0.7072	51.1963	16.6009
TCGA-78-7155-01A-11R-2039-07	TCGA-78-7155-01A	TCGA-78-7155-01A	Lung Adenocarcinoma	Upper lobe, lung	male	68	TCGA-LUAD	Chemotherapy	Carboplatin	0.6512	50.2976	0.5254
TCGA-78-7158-01A-11R-2039-07	TCGA-78-7158-01A	TCGA-78-7158-01A	Lung Adenocarcinoma	Lower lobe, lung	female	59	TCGA-LUAD	Chemotherapy	Carboplatin	2.0026	51.5236	7.449
TCGA-78-7160-01A-11R-2039-07	TCGA-78-7160-01A	TCGA-78-7160-01A	Lung Adenocarcinoma	Lower lobe, lung	male	61	TCGA-LUAD	Chemotherapy	NA	0.2296	48.859	8.6192
TCGA-78-7161-01A-11R-2039-07	TCGA-78-7161-01A	TCGA-78-7161-01A	Lung Adenocarcinoma	Upper lobe, lung	female	69	TCGA-LUAD	Chemotherapy	Cisplatin	0.3402	58.2023	3.4951
TCGA-78-7535-01A-11R-2066-07	TCGA-78-7535-01A	TCGA-78-7535-01A	Lung Adenocarcinoma	Upper lobe, lung	male	45	TCGA-LUAD	Chemotherapy	Cisplatin	3.5224	107.1721	6.0012
TCGA-78-7539-01A-11R-2066-07	TCGA-78-7539-01A	TCGA-78-7539-01A	Lung Adenocarcinoma	Lower lobe, lung	female	75	TCGA-LUAD	Chemotherapy	Carboplatin	0.9501	60.5844	10.534
TCGA-86-6562-01A-11R-1755-07	TCGA-86-6562-01A	TCGA-86-6562-01A	Lung Adenocarcinoma	Upper lobe, lung	male	52	TCGA-LUAD	Chemotherapy	Cisplatin	12.0341	46.2581	8.2115
TCGA-86-7711-01A-11R-2066-07	TCGA-86-7711-01A	TCGA-86-7711-01A	Lung Adenocarcinoma	Lower lobe, lung	male	70	TCGA-LUAD	Chemotherapy	Cisplatin	1.2819	70.86	5.8993
TCGA-86-7713-01A-11R-2066-07	TCGA-86-7713-01A	TCGA-86-7713-01A	Lung Adenocarcinoma	Middle lobe, lung	male	70	TCGA-LUAD	Chemotherapy	Cisplatin	0.4823	166.8296	0.5228
TCGA-86-7954-01A-11R-2187-07	TCGA-86-7954-01A	TCGA-86-7954-01A	Lung Adenocarcinoma	Lower lobe, lung	female	68	TCGA-LUAD	Chemotherapy	Carboplatin	4.3229	77.1352	12.1462
TCGA-86-7955-01A-11R-2187-07	TCGA-86-7955-01A	TCGA-86-7955-01A	Lung Adenocarcinoma	Lower lobe, lung	male	62	TCGA-LUAD	Chemotherapy	Cisplatin	4.9704	62.114	5.0289
TCGA-86-8054-01A-11R-2241-07	TCGA-86-8054-01A	TCGA-86-8054-01A	Lung Adenocarcinoma	Upper lobe, lung	male	61	TCGA-LUAD	Chemotherapy	Cisplatin	0.1779	56.7922	0.4573
TCGA-86-8075-01A-11R-2241-07	TCGA-86-8075-01A	TCGA-86-8075-01A	Lung Adenocarcinoma	Upper lobe, lung	female	66	TCGA-LUAD	Chemotherapy	Cisplatin	2.9816	47.064	13.1818
TCGA-86-8076-01A-31R-2241-07	TCGA-86-8076-01A	TCGA-86-8076-01A	Lung Adenocarcinoma	Upper lobe, lung	male	42	TCGA-LUAD	Chemotherapy	Cisplatin	0.9829	45.1386	3.9222
TCGA-86-8278-01A-11R-2287-07	TCGA-86-8278-01A	TCGA-86-8278-01A	Lung Adenocarcinoma	Lower lobe, lung	female	63	TCGA-LUAD	Chemotherapy	Cisplatin	3.7	59.0448	4.2374
TCGA-86-8279-01A-11R-2287-07	TCGA-86-8279-01A	TCGA-86-8279-01A	Lung Adenocarcinoma	Upper lobe, lung	male	46	TCGA-LUAD	Chemotherapy	Cisplatin	1.7404	89.3251	9.426
TCGA-86-8280-01A-11R-2287-07	TCGA-86-8280-01A	TCGA-86-8280-01A	Lung Adenocarcinoma	Lower lobe, lung	female	54	TCGA-LUAD	Chemotherapy	Cisplatin	5.4944	57.6116	3.4274
TCGA-86-8669-01A-11R-2403-07	TCGA-86-8669-01A	TCGA-86-8669-01A	Lung Adenocarcinoma	Upper lobe, lung	male	64	TCGA-LUAD	Chemotherapy	Cisplatin	0.6726	121.197	1.6705
TCGA-86-8671-01A-11R-2403-07	TCGA-86-8671-01A	TCGA-86-8671-01A	Lung Adenocarcinoma	Upper lobe, lung	female	72	TCGA-LUAD	Chemotherapy	Cisplatin	3.1355	44.5896	5.475
TCGA-86-8674-01A-21R-2403-07	TCGA-86-8674-01A	TCGA-86-8674-01A	Lung Adenocarcinoma	Middle lobe, lung	male	50	TCGA-LUAD	Chemotherapy	Cisplatin	0.8231	119.297	13.5992
TCGA-86-A4D0-01A-11R-A24H-07	TCGA-86-A4D0-01A	TCGA-86-A4D0-01A	Lung Adenocarcinoma	Upper lobe, lung	male	48	TCGA-LUAD	Chemotherapy	Cisplatin	0.2514	122.9586	0.1446
TCGA-86-A4JF-01A-11R-A24X-07	TCGA-86-A4JF-01A	TCGA-86-A4JF-01A	Lung Adenocarcinoma	Upper lobe, lung	male	56	TCGA-LUAD	Chemotherapy	NA	3.7248	49.1517	6.5887
TCGA-86-A4P8-01A-11R-A24X-07	TCGA-86-A4P8-01A	TCGA-86-A4P8-01A	Lung Adenocarcinoma	Lower lobe, lung	female	59	TCGA-LUAD	Chemotherapy	Alimta	1.2683	26.6537	1.6314
TCGA-91-6848-01A-11R-1949-07	TCGA-91-6848-01A	TCGA-91-6848-01A	Lung Adenocarcinoma	Upper lobe, lung	male	59	TCGA-LUAD	Chemotherapy	Cisplatin	0.5117	70.6906	1.1663
TCGA-91-7771-01A-11R-2170-07	TCGA-91-7771-01A	TCGA-91-7771-01A	Lung Adenocarcinoma	Upper lobe, lung	male	62	TCGA-LUAD	Chemotherapy	Cisplatin	2.1685	105.1555	4.6059
TCGA-93-A4JN-01A-11R-A24X-07	TCGA-93-A4JN-01A	TCGA-93-A4JN-01A	Lung Adenocarcinoma	Upper lobe, lung	male	71	TCGA-LUAD	Chemotherapy	Belinostat	4.9288	38.5262	10.5418

TCGA-93-A4JP-01A-11R-A24X-07	TCGA-93-A4JP-01A	TCGA-93-A4JP-01A	Lung Adenocarcinoma	Lower lobe, lung	male	64	TCGA-LUAD	Chemotherapy	Carboplatin	2.6966	57.3109	10.692
TCGA-95-7562-01A-11R-2241-07	TCGA-95-7562-01A	TCGA-95-7562-01A	Lung Adenocarcinoma	Lower lobe, lung	male	71	TCGA-LUAD	Chemotherapy	ALIMTA	0.528	117.9553	2.4819
TCGA-95-7567-01A-11R-2066-07	TCGA-95-7567-01A	TCGA-95-7567-01A	Lung Adenocarcinoma	Upper lobe, lung	male	61	TCGA-LUAD	Chemotherapy	Cisplatin	0.7515	55.0046	8.5243
TCGA-95-A4VK-01A-11R-A262-07	TCGA-95-A4VK-01A	TCGA-95-A4VK-01A	Lung Adenocarcinoma	Upper lobe, lung	female	74	TCGA-LUAD	Chemotherapy	Alimta	4.8033	29.9829	13.7268
TCGA-95-A4VP-01A-21R-A262-07	TCGA-95-A4VP-01A	TCGA-95-A4VP-01A	Lung Adenocarcinoma	Lower lobe, lung	female	66	TCGA-LUAD	Chemotherapy	Carboplatin	0.7796	73.9237	1.2997
TCGA-97-7546-01A-11R-2039-07	TCGA-97-7546-01A	TCGA-97-7546-01A	Lung Adenocarcinoma	Upper lobe, lung	female	76	TCGA-LUAD	Chemotherapy	Tarceva	5.9212	19.8845	10.5195
TCGA-97-7547-01A-11R-2039-07	TCGA-97-7547-01A	TCGA-97-7547-01A	Lung Adenocarcinoma	Upper lobe, lung	female	67	TCGA-LUAD	Chemotherapy	Alimta	8.0793	94.8594	19.2486
TCGA-97-7554-01A-11R-2039-07	TCGA-97-7554-01A	TCGA-97-7554-01A	Lung Adenocarcinoma	Lower lobe, lung	female	83	TCGA-LUAD	Chemotherapy	Alimta	3.5227	41.3877	3.0944
TCGA-97-8174-01A-11R-2287-07	TCGA-97-8174-01A	TCGA-97-8174-01A	Lung Adenocarcinoma	Upper lobe, lung	male	67	TCGA-LUAD	Chemotherapy	CISplatinum	0.6344	31.8592	4.7575
TCGA-97-8175-01A-11R-2287-07	TCGA-97-8175-01A	TCGA-97-8175-01A	Lung Adenocarcinoma	Upper lobe, lung	female	55	TCGA-LUAD	Chemotherapy	Alimta	0.2793	62.1654	14.2948
TCGA-97-8177-01A-11R-2287-07	TCGA-97-8177-01A	TCGA-97-8177-01A	Lung Adenocarcinoma	Lower lobe, lung	female	59	TCGA-LUAD	Chemotherapy	Alimta	5.7759	43.9033	6.6525
TCGA-99-7458-01A-11R-2039-07	TCGA-99-7458-01A	TCGA-99-7458-01A	Lung Adenocarcinoma	Lower lobe, lung	female	74	TCGA-LUAD	Chemotherapy	Tarceva (Erlotinib	2.4286	35.9753	4.6924
TCGA-99-8025-01A-11R-2241-07	TCGA-99-8025-01A	TCGA-99-8025-01A	Lung Adenocarcinoma	Upper lobe, lung	female	72	TCGA-LUAD	Chemotherapy	Gemzar	2.3454	103.157	3.5797
TCGA-99-8033-01A-11R-2241-07	TCGA-99-8033-01A	TCGA-99-8033-01A	Lung Adenocarcinoma	Upper lobe, lung	female	74	TCGA-LUAD	Chemotherapy	Alimta	0.3603	83.6691	3.7595
TCGA-J2-8192-01A-11R-2241-07	TCGA-J2-8192-01A	TCGA-J2-8192-01A	Lung Adenocarcinoma	Upper lobe, lung	female	65	TCGA-LUAD	Chemotherapy	Erlotinib	9.0108	38.1892	5.6506
TCGA-J2-8194-01A-11R-2241-07	TCGA-J2-8194-01A	TCGA-J2-8194-01A	Lung Adenocarcinoma	Lower lobe, lung	female	69	TCGA-LUAD	Chemotherapy	Carboplatin	0.4188	79.8623	3.6809
TCGA-L9-A743-01A-43R-A39D-07	TCGA-L9-A743-01A	TCGA-L9-A743-01A	Lung Adenocarcinoma	Upper lobe, lung	male	56	TCGA-LUAD	Chemotherapy	CISPLATIN	2.8618	75.1961	4.3725
TCGA-L9-A7SV-01A-11R-A39D-07	TCGA-L9-A7SV-01A	TCGA-L9-A7SV-01A	Lung Adenocarcinoma	Lower lobe, lung	male	69	TCGA-LUAD	Chemotherapy	cisplatin	7.4637	54.6379	1.5952
TCGA-MP-A4T8-01A-11R-A24X-07	TCGA-MP-A4T8-01A	TCGA-MP-A4T8-01A	Lung Adenocarcinoma	Upper lobe, lung	male	68	TCGA-LUAD	Chemotherapy	Cisplatin	0.3489	62.7435	2.2719
TCGA-MP-A4T9-01A-11R-A24X-07	TCGA-MP-A4T9-01A	TCGA-MP-A4T9-01A	Lung Adenocarcinoma	Upper lobe, lung	female	54	TCGA-LUAD	Chemotherapy	Cisplatin	5.8632	48.3243	5.7941
TCGA-MP-A4TC-01A-11R-A24X-07	TCGA-MP-A4TC-01A	TCGA-MP-A4TC-01A	Lung Adenocarcinoma	Middle lobe, lung	male	77	TCGA-LUAD	Chemotherapy	Cisplatin	2.4302	115.2463	6.5809
TCGA-MP-A4TD-01A-32R-A262-07	TCGA-MP-A4TD-01A	TCGA-MP-A4TD-01A	Lung Adenocarcinoma	Lower lobe, lung	male	71	TCGA-LUAD	Chemotherapy	Cisplatin	1.2579	93.3593	1.2927
TCGA-MP-A4TF-01A-11R-A262-07	TCGA-MP-A4TF-01A	TCGA-MP-A4TF-01A	Lung Adenocarcinoma	Upper lobe, lung	female	58	TCGA-LUAD	Chemotherapy	Cisplatin	0.2058	98.9616	7.3458
TCGA-NJ-A7XG-01A-12R-A39D-07	TCGA-NJ-A7XG-01A	TCGA-NJ-A7XG-01A	Lung Adenocarcinoma	Upper lobe, lung	male	49	TCGA-LUAD	Chemotherapy	Cisplatin	7.3263	75.7858	7.6897
