# Supplementary material for: Non-canonical NOTCH1 signaling regulates ferroptosis vulnerability in dormant lung cancer cells with stable resistance
Source: Cell Death Dis. 2025 Dec 26;17(1):1. doi: 10.1038/s41419-025-08355-9 (PMC12780219; doi:10.1038/s41419-025-08355-9)
Supplement: Supplementary file 8 — Supplementary Table 6 [file 41419_2025_8355_MOESM8_ESM.pdf]

Table S6. CNV scores for inferCNV cell clusters
Related to Figure S4C

No.	Seurat_cluster	Mean_CNV_score	CNV+ cancer populations
1	0	1	
2	1	1.00968354	Yes
3	2	1.004504576	
4	3	1.003301756	
5	4	1	
6	5	1.003372393	
7	6	1.007441124	
8	7	1.009243537	Yes
9	8	1.012674248	Yes
10	9	1.003775443	
11	10	1.006061474	
12	11	1.006399481	
13	12	1.008575903	
14	13	1.005581403	
15	14	1.004780931	
16	15	1.010631161	Yes
17	16	1.004408929	
18	17	1.00444822	
19	18	1.004043288	
20	19	1	
21	20	1.004805081	

Footnote:
1. CNV score generated by *InferCNV* (version 1.21.0)
2. The scRNA-seq dataset GSE131907
3. Cutoff for tumor cells: 1.009
