# Supplementary material for: Non-canonical NOTCH1 signaling regulates ferroptosis vulnerability in dormant lung cancer cells with stable resistance
Source: Cell Death Dis. 2025 Dec 26;17(1):1. doi: 10.1038/s41419-025-08355-9 (PMC12780219; doi:10.1038/s41419-025-08355-9)
Supplement: Supplementary file 9 — Supplementary Table 7 [file 41419_2025_8355_MOESM9_ESM.pdf]

Table S7. The SLC52A2^{+}NOTCH1^{+} SRCC isolated by different techniques

Related to Fig. S4I

	The *SLC52A 2*$^{+}$*NOTCH1*$^{+}$ cells via the WhichCells module		the *SLC52A2*$^{+}$*NOTCH1*$^{+}$ population via the modified clustering with marker gain		The *SLC52A2*$^{+}$*NOTCH1*$^{+}$*CD82*$^{+}$ cells within the *SLC52A2*$^{+}$*NOTCH1*$^{+}$ compartment determined by the modified clustering with marker
**No.**	**Cell ID**	**No.**	**Cell ID**	**No.**	**Cell ID**
1	AAATGCCAGAGCCCAA_NS_12	1	AAATGCCAGAGCCCAA_NS_12	1	AAATGCCAGAGCCCAA_NS_12
2	AACCGCGTCTAGAGTC_NS_12	2	AACCGCGTCTAGAGTC_NS_12	2	AACCGCGTCTAGAGTC_NS_12
3	AAGACCTAGCATCATC_NS_12	3	AAGACCTAGCATCATC_NS_12	3	AAGACCTAGCATCATC_NS_12
4	ACACCGGCAGCCAATT_NS_07	4	ACACCGGCAGCCAATT_NS_07	4	ACACCGGCAGCCAATT_NS_07
5	ACAGCTAGTATCTGCA_NS_12	5	ACAGCTAGTATCTGCA_NS_12	5	ACAGCTAGTATCTGCA_NS_12
6	ACAGCTAGTGGTCCGT_NS_12	6	ACAGCTAGTGGTCCGT_NS_12	6	ACAGCTAGTGGTCCGT_NS_12
7	ACATCAGGTGGCGAAT_NS_07	7	ACATCAGGTGGCGAAT_NS_07	7	ACATCAGGTGGCGAAT_NS_07
8	ACATGGTTCGTGTAGT_NS_12	8	ACATGGTTCGTGTAGT_NS_12	8	ACATGGTTCGTGTAGT_NS_12
9	ACCTTTAGTCGCTTTC_NS_12	9	ACCTTTAGTCGCTTTC_NS_12	9	ACCTTTAGTCGCTTTC_NS_12
10	ACGATACCAAAGGCGT_NS_12	10	ACGATACCAAAGGCGT_NS_12	10	ACGATACCAAAGGCGT_NS_12
11	ACGATGTTCACCCTCA_NS_12	11	ACGATGTTCACCCTCA_NS_12	11	ACGATGTTCACCCTCA_NS_12
12	ACGCCGAAGCGCTCCA_NS_12	12	ACGCCGAAGCGCTCCA_NS_12	12	ACGCCGAAGCGCTCCA_NS_12
13	ACTGAACAGTGACATA_NS_12	13	ACTGAACAGTGACATA_NS_12	13	ACTGAACAGTGACATA_NS_12
14	ACTGAACCAGACAAGC_NS_12	14	ACTGAACCAGACAAGC_NS_12	14	ACTGAACCAGACAAGC_NS_12
15	ACTGAACGTGTGACGA_NS_12	15	ACTGAACGTGTGACGA_NS_12	15	ACTGAACGTGTGACGA_NS_12
16	ACTGAACTCACCGGGT_NS_07	16	ACTGAACTCACCGGGT_NS_07	16	ACTGAACTCACCGGGT_NS_07
17	ACTGAGTGTATTAGCC_NS_12	17	ACTGAGTGTATTAGCC_NS_12	17	ACTGAGTGTATTAGCC_NS_12
18	ACTGAGTTCCCGACTT_NS_12	18	ACTGAGTTCCCGACTT_NS_12	18	ACTGAGTTCCCGACTT_NS_12
19	ACTGCTCTCAATACCG_NS_12	19	ACTGCTCTCAATACCG_NS_12	19	ACTGCTCTCAATACCG_NS_12
20	ACTGTCCCATAGGATA_NS_12	20	ACTGTCCCATAGGATA_NS_12	20	ACTGTCCCATAGGATA_NS_12
21	AGACGTTTCTTGCATT_NS_12	21	AGACGTTTCTTGCATT_NS_12	21	AGACGTTTCTTGCATT_NS_12
22	AGATCTGAGACAGACC_NS_07	22	AGATCTGAGACAGACC_NS_07	22	AGATCTGAGACAGACC_NS_07
23	AGATCTGGTGTGTGCC_NS_12	23	AGATCTGGTGTGTGCC_NS_12	23	AGATCTGGTGTGTGCC_NS_12
24	AGCCTAAAGAAGGTGA_NS_12	24	AGCCTAAAGAAGGTGA_NS_12	24	AGCCTAAAGAAGGTGA_NS_12
25	AGCTCCTCAATCACAC_NS_12	25	AGCTCCTCAATCACAC_NS_12	25	AGCTCCTCAATCACAC_NS_12
26	AGCTCTCAGTCGAGTG_NS_07	26	AGCTCTCAGTCGAGTG_NS_07	26	AGCTCTCAGTCGAGTG_NS_07
27	AGGGAGTCACGCCAGT_NS_12	27	AGGGAGTCACGCCAGT_NS_12	27	AGGGAGTCACGCCAGT_NS_12
28	AGGGATGCATCGGGTC_NS_07	28	AGGGATGCATCGGGTC_NS_07	28	AGGGATGCATCGGGTC_NS_07
29	AGGGATGGTAAAGTCA_NS_12	29	AGGGATGGTAAAGTCA_NS_12	29	AGGGATGGTAAAGTCA_NS_12
30	AGGGTGAAGAACAATC_NS_12	30	AGGGTGAAGAACAATC_NS_12	30	AGGGTGAAGAACAATC_NS_12
31	AGGTCATCACACTGCG_NS_12	31	AGGTCATCACACTGCG_NS_12	31	AGGTCATCACACTGCG_NS_12
32	ATAACGCAGCCCAACC_NS_12	32	ATAACGCAGCCCAACC_NS_12	32	ATAACGCAGCCCAACC_NS_12
33	ATAACGCAGTGTACCT_NS_12	33	ATAACGCAGTGTACCT_NS_12	33	ATAACGCAGTGTACCT_NS_12
34	ATCATCTGTAGCCTCG_NS_12	34	ATCATCTGTAGCCTCG_NS_12	34	ATCATCTGTAGCCTCG_NS_12
35	ATCTACTTCGTGGTCG_NS_12	35	ATCTACTTCGTGGTCG_NS_12	35	ATCTACTTCGTGGTCG_NS_12
36	ATCTGCCAGGACAGAA_NS_12	36	ATCTGCCAGGACAGAA_NS_12	36	ATCTGCCAGGACAGAA_NS_12
37	ATGCGATGTCCGAACC_NS_12	37	ATGCGATGTCCGAACC_NS_12	37	ATGCGATGTCCGAACC_NS_12

38	ATTACTCGTGTTGAGG_NS_12	38	ATTACTCGTGTTGAGG_NS_12	38	ATTACTCGTGTTGAGG_NS_12
39	CAAGTTGGTCACCCAG_NS_12	39	CAAGTTGGTCACCCAG_NS_12	39	CAAGTTGGTCACCCAG_NS_12
40	CACAAACCAATGCCAT_NS_12	40	CACAAACCAATGCCAT_NS_12	40	CACAAACCAATGCCAT_NS_12
41	CACACCTCAGCTGTTA_NS_12	41	CACACCTCAGCTGTTA_NS_12	41	CACACCTCAGCTGTTA_NS_12
42	CACACCTCATGTTGAC_NS_12	42	CACACCTCATGTTGAC_NS_12	42	CACACCTCATGTTGAC_NS_12
43	CACACCTTCGGCCGAT_NS_12	43	CACACCTTCGGCCGAT_NS_12	43	CACACCTTCGGCCGAT_NS_12
44	CAGCATAGTCATATCG_NS_12	44	CAGCATAGTCATATCG_NS_12	44	CAGCATAGTCATATCG_NS_12
45	CAGCCGAGTCTAAAGA_NS_12	45	CAGCCGAGTCTAAAGA_NS_12	45	CAGCCGAGTCTAAAGA_NS_12
46	CAGCGACTCCTTAATC_NS_12	46	CAGCGACTCCTTAATC_NS_12	46	CAGCGACTCCTTAATC_NS_12
47	CAGTAACTCCGCGGTA_NS_07	47	CAGTAACTCCGCGGTA_NS_07	47	CAGTAACTCCGCGGTA_NS_07
48	CATCAAGCATTACCTT_NS_12	48	CATCAAGCATTACCTT_NS_12	48	CATCAAGCATTACCTT_NS_12
49	CATCAAGTCCTTGACC_NS_12	49	CATCAAGTCCTTGACC_NS_12	49	CATCAAGTCCTTGACC_NS_12
50	CATCGGGGTAACGCGA_NS_12	50	CATCGGGGTAACGCGA_NS_12	50	CATCGGGGTAACGCGA_NS_12
51	CATTCGCCAACTGGCC_NS_07	51	CATTCGCCAACTGGCC_NS_07	51	CATTCGCCAACTGGCC_NS_07
52	CCACTACCATGGAATA_NS_12	52	CCACTACCATGGAATA_NS_12	52	CCACTACCATGGAATA_NS_12
53	CCATGTCCATTCGACA_NS_12	53	CCATGTCCATTCGACA_NS_12	53	CCATGTCCATTCGACA_NS_12
54	CCATTCGCACAGCCCA_NS_12	54	CCATTCGCACAGCCCA_NS_12	54	CCATTCGCACAGCCCA_NS_12
55	CCCAGTTCAGTGAGTG_NS_07	55	CCCAGTTCAGTGAGTG_NS_07	55	CCCAGTTCAGTGAGTG_NS_07
56	CCCAGTTTCATGCTCC_NS_12	56	CCCAGTTTCATGCTCC_NS_12	56	CCCAGTTTCATGCTCC_NS_12
57	CCGGTAGGTTGGACCC_NS_12	57	CCGGTAGGTTGGACCC_NS_12	57	CCGGTAGGTTGGACCC_NS_12
58	CCGTTCACACATGGGA_NS_12	58	CCGTTCACACATGGGA_NS_12	58	CCGTTCACACATGGGA_NS_12
59	CCGTTCAGTACAAGTA_NS_12	59	CCGTTCAGTACAAGTA_NS_12	59	CCGTTCAGTACAAGTA_NS_12
60	CCTAAAGGTACATCCA_NS_12	60	CCTAAAGGTACATCCA_NS_12	60	CCTAAAGGTACATCCA_NS_12
61	CCTAAAGGTGAACCTT_NS_12	61	CCTAAAGGTGAACCTT_NS_12	61	CCTAAAGGTGAACCTT_NS_12
62	CCTATTAAGCTAGCCC_NS_07	62	CCTATTAAGCTAGCCC_NS_07	62	CCTATTAAGCTAGCCC_NS_07
63	CCTCAGTCAATCACAC_NS_12	63	CCTCAGTCAATCACAC_NS_12	63	CCTCAGTCAATCACAC_NS_12
64	CCTCTGACAGGCTCAC_NS_12	64	CCTCTGACAGGCTCAC_NS_12	64	CCTCTGACAGGCTCAC_NS_12
65	CCTTACGTCGTTTGCC_NS_12	65	CCTTACGTCGTTTGCC_NS_12	65	CCTTACGTCGTTTGCC_NS_12
66	CCTTCGAAGACAGACC_NS_12	66	CCTTCGAAGACAGACC_NS_12	66	CCTTCGAAGACAGACC_NS_12
67	CCTTTCTGTGATAAGT_NS_12	67	CCTTTCTGTGATAAGT_NS_12	67	CCTTTCTGTGATAAGT_NS_12
68	CGAACATGTAAGCACG_NS_12	68	CGAACATGTAAGCACG_NS_12	68	CGAACATGTAAGCACG_NS_12
69	CGATCGGAGACGCACA_NS_12	69	CGATCGGAGACGCACA_NS_12	69	CGATCGGAGACGCACA_NS_12
70	CGCCAAGCAACGATGG_NS_12	70	CGCCAAGCAACGATGG_NS_12	70	CGCCAAGCAACGATGG_NS_12
71	CGCGTTTGTACTCAAC_NS_12	71	CGCGTTTGTACTCAAC_NS_12	71	CGCGTTTGTACTCAAC_NS_12
72	CGGACACGTAGAAGGA_NS_07	72	CGGACACGTAGAAGGA_NS_07	72	CGGACACGTAGAAGGA_NS_07
73	CGTAGGCCACAGACTT_NS_07	73	CGTAGGCCACAGACTT_NS_07	73	CGTAGGCCACAGACTT_NS_07
74	CTAATGGAGGAATGGA_NS_12	74	CTAATGGAGGAATGGA_NS_12	74	CTAATGGAGGAATGGA_NS_12
75	CTAATGGGTAGAGGAA_NS_07	75	CTAATGGGTAGAGGAA_NS_07	75	CTAATGGGTAGAGGAA_NS_07
76	CTACACCGTAAACACA_NS_12	76	CTACACCGTAAACACA_NS_12	76	CTACACCGTAAACACA_NS_12
77	CTACATTTCCACTCCA_NS_12	77	CTACATTTCCACTCCA_NS_12	77	CTACATTTCCACTCCA_NS_12
78	CTACATTTCGTGGGAA_NS_12	78	CTACATTTCGTGGGAA_NS_12	78	CTACATTTCGTGGGAA_NS_12
79	CTACGTCGTTTGACTG_NS_12	79	CTACGTCGTTTGACTG_NS_12	79	CTACGTCGTTTGACTG_NS_12
80	CTCATTAGTGCAGTAG_NS_12	80	CTCATTAGTGCAGTAG_NS_12	80	CTCATTAGTGCAGTAG_NS_12
81	CTCGAAACATACTACG_NS_07	81	CTCGAAACATACTACG_NS_07	81	CTCGAAACATACTACG_NS_07
82	CTCGAGGTCCACTCCA_NS_12	82	CTCGAGGTCCACTCCA_NS_12	82	CTCGAGGTCCACTCCA_NS_12
83	CTCGTACCAAGCGAGT_NS_07	83	CTCGTACCAAGCGAGT_NS_07	83	CTCGTACCAAGCGAGT_NS_07
84	CTCGTCAGTAAAGTCA_NS_07	84	CTCGTCAGTAAAGTCA_NS_07	84	CTCGTCAGTAAAGTCA_NS_07
85	CTGAAACGTAGGCATG_NS_07	85	CTGAAACGTAGGCATG_NS_07	85	CTGAAACGTAGGCATG_NS_07
86	CTGCCTATCCTAGGGC_NS_12	86	CTGCCTATCCTAGGGC_NS_12	86	CTGCCTATCCTAGGGC_NS_12
87	CTGCTGTAGGGTCTCC_NS_07	87	CTGCTGTAGGGTCTCC_NS_07	87	CTGCTGTAGGGTCTCC_NS_07

88	CTTACCGAGGGAACGG_NS_12	88	CTTACCGAGGGAACGG_NS_12	88	CTTACCGAGGGAACGG_NS_12
89	CTTGGCTGTCGTCTTC_NS_12	89	CTTGGCTGTCGTCTTC_NS_12	89	CTTGGCTGTCGTCTTC_NS_12
90	CTTTGCGAGAGTACCG_NS_12	90	CTTTGCGAGAGTACCG_NS_12	90	CTTTGCGAGAGTACCG_NS_12
91	GAAATGACACACCGAC_NS_07	91	GAAATGACACACCGAC_NS_07	91	GAAATGACACACCGAC_NS_07
92	GAAATGACATACCATG_NS_12	92	GAAATGACATACCATG_NS_12	92	GAAATGACATACCATG_NS_12
93	GAACGGAAGTGGTCCC_NS_12	93	GAACGGAAGTGGTCCC_NS_12	93	GAACGGAAGTGGTCCC_NS_12
94	GAAGCAGCATCGACGC_NS_12	94	GAAGCAGCATCGACGC_NS_12	94	GAAGCAGCATCGACGC_NS_12
95	GACAGAGCAAGTAATG_NS_12	95	GACAGAGCAAGTAATG_NS_12	95	GACAGAGCAAGTAATG_NS_12
96	GACCAATCATCGTCGG_NS_07	96	GACCAATCATCGTCGG_NS_07	96	GACCAATCATCGTCGG_NS_07
97	GACTAACTCTTCGAGA_NS_12	97	GACTAACTCTTCGAGA_NS_12	97	GACTAACTCTTCGAGA_NS_12
98	GATCAGTCAGCTCGCA_NS_12	98	GATCAGTCAGCTCGCA_NS_12	98	GATCAGTCAGCTCGCA_NS_12
99	GATCGCGTCACGGTTA_NS_12	99	GATCGCGTCACGGTTA_NS_12	99	GATCGCGTCACGGTTA_NS_12
100	GATCGTAGTTTAGGAA_NS_07	100	GATCGTAGTTTAGGAA_NS_07	100	GATCGTAGTTTAGGAA_NS_07
101	GATTCAGGTCTCCACT_NS_07	101	GATTCAGGTCTCCACT_NS_07	101	GATTCAGGTCTCCACT_NS_07
102	GCATACATCTCACATT_NS_12	102	GCATACATCTCACATT_NS_12	102	GCATACATCTCACATT_NS_12
103	GCGGGTTCAAGGCTCC_NS_12	103	GCGGGTTCAAGGCTCC_NS_12	103	GCGGGTTCAAGGCTCC_NS_12
104	GCTCCTACAAGCCATT_NS_12	104	GCTCCTACAAGCCATT_NS_12	104	GCTCCTACAAGCCATT_NS_12
105	GGACAAGAGTCTCCTC_NS_07	105	GGACAAGAGTCTCCTC_NS_07	105	GGACAAGAGTCTCCTC_NS_07
106	GGACATTTCATATCGG_NS_12	106	GGACATTTCATATCGG_NS_12	106	GGACATTTCATATCGG_NS_12
107	GGAGCAACAATCACAC_NS_12	107	GGAGCAACAATCACAC_NS_12	107	GGAGCAACAATCACAC_NS_12
108	GGCAATTCACCGAAAG_NS_12	108	GGCAATTCACCGAAAG_NS_12	108	GGCAATTCACCGAAAG_NS_12
109	GGCTCGAAGCCGGTAA_NS_07	109	GGCTCGAAGCCGGTAA_NS_07	109	GGCTCGAAGCCGGTAA_NS_07
110	GGGAATGGTCACCCAG_NS_12	110	GGGAATGGTCACCCAG_NS_12	110	GGGAATGGTCACCCAG_NS_12
111	GGGCACTAGATAGTCA_NS_07	111	GGGCACTAGATAGTCA_NS_07	111	GGGCACTAGATAGTCA_NS_07
112	GGTGAAGCAGATCCAT_NS_07	112	GGTGAAGCAGATCCAT_NS_07	112	GGTGAAGCAGATCCAT_NS_07
113	GGTGAAGGTTAGGGTG_NS_07	113	GGTGAAGGTTAGGGTG_NS_07	113	GGTGAAGGTTAGGGTG_NS_07
114	GTAACGTCACAACTGT_NS_12	114	GTAACGTCACAACTGT_NS_12	114	GTAACGTCACAACTGT_NS_12
115	GTACTCCAGACGCTTT_NS_12	115	GTACTCCAGACGCTTT_NS_12	115	GTACTCCAGACGCTTT_NS_12
116	GTACTTTTCTGATACG_NS_12	116	GTACTTTTCTGATACG_NS_12	116	GTACTTTTCTGATACG_NS_12
117	GTAGTCAAGCCGATTT_NS_12	117	GTAGTCAAGCCGATTT_NS_12	117	GTAGTCAAGCCGATTT_NS_12
118	GTCAAGTAGCTAAACA_NS_12	118	GTCAAGTAGCTAAACA_NS_12	118	GTCAAGTAGCTAAACA_NS_12
119	GTCAAGTCAATGGTCT_NS_12	119	GTCAAGTCAATGGTCT_NS_12	119	GTCAAGTCAATGGTCT_NS_12
120	GTCACAAGTATTCGTG_NS_12	120	GTCACAAGTATTCGTG_NS_12	120	GTCACAAGTATTCGTG_NS_12
121	GTCATTTCATCCGCGA_NS_12	121	GTCATTTCATCCGCGA_NS_12	121	GTCATTTCATCCGCGA_NS_12
122	GTGTGCGGTAAGCACG_NS_12	122	GTGTGCGGTAAGCACG_NS_12	122	GTGTGCGGTAAGCACG_NS_12
123	GTTAAGCGTACATCCA_NS_12	123	GTTAAGCGTACATCCA_NS_12	123	GTTAAGCGTACATCCA_NS_12
124	GTTTCTAGTATCACCA_NS_12	124	GTTTCTAGTATCACCA_NS_12	124	GTTTCTAGTATCACCA_NS_12
125	TACACGAGTCCGAACC_NS_12	125	TACACGAGTCCGAACC_NS_12	125	TACACGAGTCCGAACC_NS_12
126	TACAGTGTCCTAGAAC_NS_12	126	TACAGTGTCCTAGAAC_NS_12	126	TACAGTGTCCTAGAAC_NS_12
127	TACGGGCTCTGCAGTA_NS_12	127	TACGGGCTCTGCAGTA_NS_12	127	TACGGGCTCTGCAGTA_NS_12
128	TATCAGGCAGTAACGG_NS_12	128	TATCAGGCAGTAACGG_NS_12	128	TATCAGGCAGTAACGG_NS_12
129	TATTACCTCGGCGCAT_NS_07	129	TATTACCTCGGCGCAT_NS_07	129	TATTACCTCGGCGCAT_NS_07
130	TCACGAAAGATAGCAT_NS_12	130	TCACGAAAGATAGCAT_NS_12	130	TCACGAAAGATAGCAT_NS_12
131	TCACGAAGTCAGGACA_NS_12	131	TCACGAAGTCAGGACA_NS_12	131	TCACGAAGTCAGGACA_NS_12
132	TCAGCAACACTAAGTC_NS_12	132	TCAGCAACACTAAGTC_NS_12	132	TCAGCAACACTAAGTC_NS_12
133	TCCCGATGTAGTGAAT_NS_12	133	TCCCGATGTAGTGAAT_NS_12	133	TCCCGATGTAGTGAAT_NS_12
134	TCGGTAAAGACTTTCG_NS_07	134	TCGGTAAAGACTTTCG_NS_07	134	TCGGTAAAGACTTTCG_NS_07
135	TCTCTAAAGCGATATA_NS_12	135	TCTCTAAAGCGATATA_NS_12	135	TCTCTAAAGCGATATA_NS_12
136	TGAAAGAAGCACAGGT_NS_12	136	TGAAAGAAGCACAGGT_NS_12	136	TGAAAGAAGCACAGGT_NS_12
137	TGAGCATGTTCCCTTG_NS_12	137	TGAGCATGTTCCCTTG_NS_12	137	TGAGCATGTTCCCTTG_NS_12

138	TGAGCCGGTATATGAG_NS_12
139	TGCCCATAGGCGTACA_NS_12
140	TGCGCAGAGATGTTAG_NS_12
141	TGCGCAGCAGATCGGA_NS_12
142	TGCGTGGCAAACCTAC_NS_07
143	TGGACGCTCTCAACTT_NS_12
144	TGGCTGGAGGCCCTTG_NS_07
145	TGGTTAGTCTGTCAAG_NS_07
146	TGTGTTTCAAGTACCT_NS_07
147	TTATGCTCACTGTTAG_NS_12
148	TTGTAGGTCCCTAACC_NS_12
149	TTTACTGAGTCAAGCG_NS_12
150	TTTACTGGTCGAATCT_NS_12
151	TTTGGTTCAGGTGGAT_NS_12
152	TTTGTCAAGCACCGTC_NS_12
153	AAAGTAGGTATCTGCA_NS_07
154	AACTGGTGTTCGTCTC_NS_07
155	AACTGGTTCAGTCAGT_NS_07
156	AAGCCGCAGAGACGAA_NS_07
157	AAGCCGCGTATAAACG_NS_07
158	AAGTCTGGTGGTCCGT_NS_07
159	ACACTGAAGTACGCGA_NS_07
160	ACAGCTACAATGGACG_NS_07
161	ACGAGGAGTCCGTGAC_NS_07
162	AGCTCCTCAGCGTTCG_NS_07
163	AGCTCTCCACGACGAA_NS_07
164	AGGGATGTCACATAGC_NS_07
165	AGTAGTCTCTGACCTC_NS_07
166	AGTCTTTTCTCATTCA_NS_07
167	AGTGAGGCACAGCGTC_NS_07
168	AGTTGGTGTTCCACTC_NS_07
169	ATAGACCGTGGACGAT_NS_07
170	ATCATCTGTATAGTAG_NS_07
171	ATGAGGGTCTCTGTCG_NS_07
172	ATGTGTGAGCCAGTTT_NS_07
173	ATTATCCCAGTTCATG_NS_07
174	ATTCTACGTCACCTAA_NS_07
175	ATTGGACCATGAAGTA_NS_07
176	CACCACTCATGTCGAT_NS_07
177	CACTCCATCTTTACAC_NS_07
178	CAGCATAAGGAGCGTT_NS_07
179	CAGCATACATCACGTA_NS_07
180	CAGGTGCCACGCTTTC_NS_07
181	CAGTAACCAATACGCT_NS_07
182	CATCAAGAGATCGATA_NS_07
183	CATCGAAAGTACGCGA_NS_07
184	CATGCCTGTGATAAAC_NS_07
185	CATGGCGGTGACTCAT_NS_07
186	CATTCGCGTCTAGGTT_NS_07
187	CCGTACTGTCTAACGT_NS_07

138	TGAGCCGGTATATGAG_NS_12
139	TGCCCATAGGCGTACA_NS_12
140	TGCGCAGAGATGTTAG_NS_12
141	TGCGCAGCAGATCGGA_NS_12
142	TGCGTGGCAAACCTAC_NS_07
143	TGGACGCTCTCAACTT_NS_12
144	TGGCTGGAGGCCCTTG_NS_07
145	TGGTTAGTCTGTCAAG_NS_07
146	TGTGTTTCAAGTACCT_NS_07
147	TTATGCTCACTGTTAG_NS_12
148	TTGTAGGTCCCTAACC_NS_12
149	TTTACTGAGTCAAGCG_NS_12
150	TTTACTGGTCGAATCT_NS_12
151	TTTGGTTCAGGTGGAT_NS_12
152	TTTGTCAAGCACCGTC_NS_12
153	AAAGTAGGTATCTGCA_NS_07
154	AACTGGTGTTCGTCTC_NS_07
155	AACTGGTTCAGTCAGT_NS_07
156	AAGCCGCAGAGACGAA_NS_07
157	AAGCCGCGTATAAACG_NS_07
158	AAGTCTGGTGGTCCGT_NS_07
159	ACACTGAAGTACGCGA_NS_07
160	ACAGCTACAATGGACG_NS_07
161	ACGAGGAGTCCGTGAC_NS_07
162	AGCTCCTCAGCGTTCG_NS_07
163	AGCTCTCCACGACGAA_NS_07
164	AGGGATGTCACATAGC_NS_07
165	AGTAGTCTCTGACCTC_NS_07
166	AGTCTTTTCTCATTCA_NS_07
167	AGTGAGGCACAGCGTC_NS_07
168	AGTTGGTGTTCCACTC_NS_07
169	ATAGACCGTGGACGAT_NS_07
170	ATCATCTGTATAGTAG_NS_07
171	ATGAGGGTCTCTGTCG_NS_07
172	ATGTGTGAGCCAGTTT_NS_07
173	ATTATCCCAGTTCATG_NS_07
174	ATTCTACGTCACCTAA_NS_07
175	ATTGGACCATGAAGTA_NS_07
176	CACCACTCATGTCGAT_NS_07
177	CACTCCATCTTTACAC_NS_07
178	CAGCATAAGGAGCGTT_NS_07
179	CAGCATACATCACGTA_NS_07
180	CAGGTGCCACGCTTTC_NS_07
181	CAGTAACCAATACGCT_NS_07
182	CATCAAGAGATCGATA_NS_07
183	CATCGAAAGTACGCGA_NS_07
184	CATGCCTGTGATAAAC_NS_07
185	CATGGCGGTGACTCAT_NS_07
186	CATTCGCGTCTAGGTT_NS_07
187	CCGTACTGTCTAACGT_NS_07

138	TGAGCCGGTATATGAG_NS_12
139	TGCCCATAGGCGTACA_NS_12
140	TGCGCAGAGATGTTAG_NS_12
141	TGCGCAGCAGATCGGA_NS_12
142	TGCGTGGCAAACCTAC_NS_07
143	TGGACGCTCTCAACTT_NS_12
144	TGGCTGGAGGCCCTTG_NS_07
145	TGGTTAGTCTGTCAAG_NS_07
146	TGTGTTTCAAGTACCT_NS_07
147	TTATGCTCACTGTTAG_NS_12
148	TTGTAGGTCCCTAACC_NS_12
149	TTTACTGAGTCAAGCG_NS_12
150	TTTACTGGTCGAATCT_NS_12
151	TTTGGTTCAGGTGGAT_NS_12
152	TTTGTCAAGCACCGTC_NS_12

188	CCTAGCTTCTTCGAGA_NS_07
189	CCTTCCCTCCCGACTT_NS_07
190	CGATGGCGTAACGACG_NS_07
191	CGCTATCTCACTCCTG_NS_07
192	CGGACTGTCACGATGT_NS_07
193	CGGAGCTCAGCCACCA_NS_07
194	CTAAGACTCTTAGCCC_NS_07
195	CTACACCTCTGGTTCC_NS_07
196	CTACCCAAGACACGAC_NS_07
197	CTACGTCAGAATAGGG_NS_07
198	CTAGAGTGTTACTGAC_NS_07
199	CTAGAGTTCACCACCT_NS_07
200	CTAGCCTCATCGGTTA_NS_07
201	CTGATAGCACGAAATA_NS_07
202	CTTTGCGTCCACGAAT_NS_07
203	GAACGGAGTGAGCGAT_NS_07
204	GACCAATTCAGCTGGC_NS_07
205	GACCTGGAGGACTGGT_NS_07
206	GAGCAGACAACTTGAC_NS_07
207	GAGCAGAGTCTGATTG_NS_07
208	GCACTCTAGCGTCTAT_NS_07
209	GCATGCGCAGCATGAG_NS_07
210	GCCTCTACAGATGGGT_NS_07
211	GCGACCAGTAAGAGGA_NS_07
212	GCGGGTTGTTTGGCGC_NS_07
213	GCTCCTACACATGACT_NS_07
214	GGAATAAGTTCGGCAC_NS_07
215	GGCTCGAGTGTGAATA_NS_12
216	GGGACCTGTCTCCCTA_NS_07
217	GGGTCTGGTAAGAGGA_NS_12
218	GGTGTTACACGGTAGA_NS_07
219	GTGAAGGGTCCGTCAG_NS_07
220	GTGTTAGTCAAACCGT_NS_07
221	GTTCGGGAGGTGTTAA_NS_07
222	TACCTATTCTCGGACG_NS_07
223	TACCTATTCTGGTGTA_NS_07
224	TACTCGCCATCGTCGG_NS_07
225	TATCAGGCAGTATAAG_NS_07
226	TCATTACCAGACGCTC_NS_07
227	TCCACACAGAAACCGC_NS_07
228	TCGAGGCTCCATGCTC_NS_07
229	TCGCGAGGTCCAGTAT_NS_07
230	TCTGAGAGTCACTGGC_NS_07
231	TCTGGAATCATAGCAC_NS_07
232	TGAGGGAAGCTGCAAG_NS_07
233	TGCCCTAGTCGACTGC_NS_12
234	TTCTACAAGGCCGAAT_NS_07
235	TTCTTAGTCCGTTGCT_NS_07
236	TTGGCAAGTCTGATTG_NS_07
237	TTTCCTCCAAGAGGCT_NS_07

188	CCTAGCTTCTTCGAGA_NS_07
189	CCTTCCCTCCCGACTT_NS_07
190	CGATGGCGTAACGACG_NS_07
191	CGCTATCTCACTCCTG_NS_07
192	CGGACTGTCACGATGT_NS_07
193	CGGAGCTCAGCCACCA_NS_07
194	CTAAGACTCTTAGCCC_NS_07
195	CTACACCTCTGGTTCC_NS_07
196	CTACCCAAGACACGAC_NS_07
197	CTACGTCAGAATAGGG_NS_07
198	CTAGAGTGTTACTGAC_NS_07
199	CTAGAGTTCACCACCT_NS_07
200	CTAGCCTCATCGGTTA_NS_07
201	CTGATAGCACGAAATA_NS_07
202	CTTTGCGTCCACGAAT_NS_07
203	GAACGGAGTGAGCGAT_NS_07
204	GACCAATTCAGCTGGC_NS_07
205	GACCTGGAGGACTGGT_NS_07
206	GAGCAGACAACTTGAC_NS_07
207	GAGCAGAGTCTGATTG_NS_07
208	GCACTCTAGCGTCTAT_NS_07
209	GCATGCGCAGCATGAG_NS_07
210	GCCTCTACAGATGGGT_NS_07
211	GCGACCAGTAAGAGGA_NS_07
212	GCGGGTTGTTTGGCGC_NS_07
213	GCTCCTACACATGACT_NS_07
214	GGAATAAGTTCGGCAC_NS_07
215	GGCTCGAGTGTGAATA_NS_12
216	GGGACCTGTCTCCCTA_NS_07
217	GGGTCTGGTAAGAGGA_NS_12
218	GGTGTTACACGGTAGA_NS_07
219	GTGAAGGGTCCGTCAG_NS_07
220	GTGTTAGTCAAACCGT_NS_07
221	GTTCGGGAGGTGTTAA_NS_07
222	TACCTATTCTCGGACG_NS_07
223	TACCTATTCTGGTGTA_NS_07
224	TACTCGCCATCGTCGG_NS_07
225	TATCAGGCAGTATAAG_NS_07
226	TCATTACCAGACGCTC_NS_07
227	TCCACACAGAAACCGC_NS_07
228	TCGAGGCTCCATGCTC_NS_07
229	TCGCGAGGTCCAGTAT_NS_07
230	TCTGAGAGTCACTGGC_NS_07
231	TCTGGAATCATAGCAC_NS_07
232	TGAGGGAAGCTGCAAG_NS_07
233	TGCCCTAGTCGACTGC_NS_12
234	TTCTACAAGGCCGAAT_NS_07
235	TTCTTAGTCCGTTGCT_NS_07
236	TTGGCAAGTCTGATTG_NS_07
237	TTTCCTCCAAGAGGCT_NS_07

238	AACGTTGGTCAAAGAT_NS_12	238	AACGTTGGTCAAAGAT_NS_12
239	AACTCAGTCCCACTTG_NS_12	239	AACTCAGTCCCACTTG_NS_12
240	ACACCAAGTTCAGACT_NS_12	240	ACACCAAGTTCAGACT_NS_12
241	ACAGCTAGTGGCTCCA_NS_12	241	ACAGCTAGTGGCTCCA_NS_12
242	ACGCAGCTCACCCTCA_NS_12	242	ACGCAGCTCACCCTCA_NS_12
243	ACGCAGCTCTTACCGC_NS_12	243	ACGCAGCTCTTACCGC_NS_12
244	ACGCCGAAGACCTTTG_NS_12	244	ACGCCGAAGACCTTTG_NS_12
245	ACTGAACGTGCACGAA_NS_12	245	ACTGAACGTGCACGAA_NS_12
246	ACTTTCACACTTCGAA_NS_12	246	ACTTTCACACTTCGAA_NS_12
247	AGACGTTGTTAAAGAC_NS_12	247	AGACGTTGTTAAAGAC_NS_12
248	AGATCTGCAGGGCATA_NS_12	248	AGATCTGCAGGGCATA_NS_12
249	AGCGTATAGGGTCTCC_NS_12	249	AGCGTATAGGGTCTCC_NS_12
250	AGGGAGTTCATAACCG_NS_12	250	AGGGAGTTCATAACCG_NS_12
251	AGTGAGGTCCATGAGT_NS_12	251	AGTGAGGTCCATGAGT_NS_12
252	ATCTGCCCACAAGACG_NS_12	252	ATCTGCCCACAAGACG_NS_12
253	CAACCTCTCAGTTTGG_NS_12	253	CAACCTCTCAGTTTGG_NS_12
254	CACAAACCAGGCTGAA_NS_12	254	CACAAACCAGGCTGAA_NS_12
255	CACAGTATCCTTTCGG_NS_12	255	CACAGTATCCTTTCGG_NS_12
256	CAGCGACCAGTATCTG_NS_12	256	CAGCGACCAGTATCTG_NS_12
257	CAGCTGGGTATGCTTG_NS_12	257	CAGCTGGGTATGCTTG_NS_12
258	CCCATACCAAGTCTAC_NS_12	258	CCCATACCAAGTCTAC_NS_12
259	CCTAGCTGTCCATCCT_NS_12	259	CCTAGCTGTCCATCCT_NS_12
260	CGAGCACAGGGTTTCT_NS_12	260	CGAGCACAGGGTTTCT_NS_12
261	CGATGTAAGGTGATAT_NS_12	261	CGATGTAAGGTGATAT_NS_12
262	CGTCCATGTTGAGTTC_NS_12	262	CGTCCATGTTGAGTTC_NS_12
263	CTCTGGTAGGGTGTTG_NS_12	263	CTCTGGTAGGGTGTTG_NS_12
264	CTGTTTACAGCTCGAC_NS_12	264	CTGTTTACAGCTCGAC_NS_12
265	GAACCTAGTAGCTCCG_NS_12	265	GAACCTAGTAGCTCCG_NS_12
266	GACGTTAAGCCATCGC_NS_12	266	GACGTTAAGCCATCGC_NS_12
267	GACGTTAGTGGTGTAG_NS_12	267	GACGTTAGTGGTGTAG_NS_12
268	GCATGCGGTAGTACCT_NS_12	268	GCATGCGGTAGTACCT_NS_12
269	GCGCGATAGCCATCGC_NS_12	269	GCGCGATAGCCATCGC_NS_12
270	GGCGTGTGTGGACGAT_NS_12	270	GGCGTGTGTGGACGAT_NS_12
271	GTGTGCGTCTACTCAT_NS_12	271	GTGTGCGTCTACTCAT_NS_12
272	TAAACCGAGGTGATAT_NS_12	272	TAAACCGAGGTGATAT_NS_12
273	TACGGATTCTTGCAAG_NS_12	273	TACGGATTCTTGCAAG_NS_12
274	TAGACCAGTACGCTGC_NS_12	274	TAGACCAGTACGCTGC_NS_12
275	TAGCCGGAGCCAGTTT_NS_12	275	TAGCCGGAGCCAGTTT_NS_12
276	TCAGCAAAGACGCAAC_NS_12	276	TCAGCAAAGACGCAAC_NS_12
277	TCAGCAACAGCCAATT_NS_12	277	TCAGCAACAGCCAATT_NS_12
278	TCGCGTTCACTCGACG_NS_12	278	TCGCGTTCACTCGACG_NS_12
279	TCGCGTTTCTCCAGGG_NS_12	279	TCGCGTTTCTCCAGGG_NS_12
280	TCTTCGGAGTGTACGG_NS_12	280	TCTTCGGAGTGTACGG_NS_12
281	TGCGCAGAGACAATAC_NS_12	281	TGCGCAGAGACAATAC_NS_12
282	TGGACGCCATCCCACT_NS_12	282	TGGACGCCATCCCACT_NS_12
283	TGTCCCAGTGTGACGA_NS_12	283	TGTCCCAGTGTGACGA_NS_12
284	TTAGGCATCGACCAGC_NS_12	284	TTAGGCATCGACCAGC_NS_12
285	TTGACTTTCTTCAACT_NS_12	285	TTGACTTTCTTCAACT_NS_12
286	AAACCTGGTACTTGAC_NS_07		
287	AAACGGGAGTAGGTGC_NS_03		

288	AAATGCCAGCTAGTCT_NS_03
289	AAATGCCCAAGTCTAC_NS_03
290	AACGTTGGTAACGTTC_NS_12
291	AACTTTCAGTGCGATG_NS_12
292	AACTTTCCAGCCTTTC_NS_03
293	ACACCCTCACCGAATT_NS_12
294	ACACTGAGTGGTCTCG_NS_07
295	ACATCAGAGGCAGGTT_NS_12
296	ACGAGGATCTTCGAGA_NS_03
297	ACGCCGAAGTACGTTC_NS_03
298	ACGGGTCTCTGCAGTA_NS_03
299	ACTATCTTCTAACTGG_NS_03
300	ACTGATGAGACCTAGG_NS_03
301	ACTTGTTAGTGGACGT_NS_03
302	AGATCTGGTCAGAATA_NS_07
303	AGATTGCAGACATAAC_NS_03
304	AGCGGTCAGCTCTCGG_NS_12
305	AGCTCTCTCTTGTCAT_NS_12
306	AGGCCACGTGATAAAC_NS_03
307	AGGCCGTTCCGTACAA_NS_17
308	AGGTCATGTCGGATCC_NS_03
309	AGTCTTTTCGGCTACG_NS_12
310	AGTTGGTAGGGAACGG_NS_03
311	ATCCACCGTGGCAAAC_NS_03
312	ATCCGAAAGCGCCTCA_NS_12
313	ATCCGAAAGGATTCGG_NS_07
314	ATGCGATAGTAGCGGT_NS_03
315	ATTACTCAGACCTTTG_NS_17
316	ATTACTCCAGACACTT_NS_12
317	ATTACTCGTAGAGCTG_NS_12
318	CAAGAAAAGGATGCGT_NS_12
319	CAAGAAACATAGAAAC_NS_17
320	CAAGATCGTGGGTATG_NS_17
321	CACATAGAGTGGAGAA_NS_12
322	CACATAGCAGGATCGA_NS_03
323	CACATAGTCACATGCA_NS_12
324	CACCAGGAGACAATAC_NS_17
325	CACCAGGGTCCAGTTA_NS_03
326	CAGAATCCAGGAATGC_NS_12
327	CAGAGAGGTCCCGACA_NS_17
328	CAGCCGATCCGCATCT_NS_12
329	CAGTAACAGACGACGT_NS_17
330	CATCAAGAGGAGTTTA_NS_03
331	CATCGAACATCATCCC_NS_07
332	CATTATCCAGGTCCAC_NS_12
333	CCACTACCACTGTTAG_NS_03
334	CCAGCGAAGAAACCGC_NS_12
335	CCCAGTTGTTCAGTAC_NS_12
336	CCCATACCAGTATGCT_NS_03
337	CCCTCCTGTTGGTAAA_NS_03

338	CCGTTCAGTGATGTCT_NS_03
339	CCTCTGAAGTGGGCTA_NS_07
340	CCTTCGACAAATTGCC_NS_17
341	CGAGCACTCGTTTGCC_NS_12
342	CGATCGGTCAGTCAGT_NS_03
343	CGCTATCTCTGGCGTG_NS_12
344	CGCTGGAAGTGCCATT_NS_03
345	CGGAGCTTCAGTGCAT_NS_17
346	CGGAGCTTCCTACAGA_NS_12
347	CGGCTAGAGTGCCATT_NS_12
348	CGTTAGAAGACAAAGG_NS_03
349	CGTTAGACAGTAACGG_NS_03
350	CTAATGGCACCTGGTG_NS_03
351	CTACACCGTTAGATGA_NS_03
352	CTACCCAGTAAACGCG_NS_17
353	CTACGTCGTCTAAACC_NS_03
354	CTAGCCTCAGGTGGAT_NS_12
355	CTAGTGACAGGGCATA_NS_03
356	CTCGTACAGTGCGTGA_NS_03
357	CTCTACGAGATCCTGT_NS_12
358	CTGAAGTAGGATGGTC_NS_12
359	CTGATAGTCCACGACG_NS_17
360	CTTGGCTTCACCTCGT_NS_12
361	GAAATGACAGATTGCT_NS_17
362	GAAATGACATCAGTCA_NS_12
363	GAATAAGTCCTGCAGG_NS_03
364	GAATGAAGTCTCTCGT_NS_03
365	GACAGAGCATTCCTCG_NS_03
366	GACCAATGTATCTGCA_NS_17
367	GACGTGCTCCGTTGTC_NS_03
368	GACGTTACAGACGCAA_NS_03
369	GACGTTACAGCGTCCA_NS_12
370	GACTAACGTAAACCTC_NS_12
371	GACTACAAGGCTAGGT_NS_03
372	GACTGCGAGATGTGTA_NS_03
373	GATCGCGAGATCTGAA_NS_03
374	GATCGCGAGTCCGTAT_NS_12
375	GATCGTAAGTACGCCC_NS_12
376	GATGAGGCACGCTTTC_NS_17
377	GATGCTAAGCGTCTAT_NS_17
378	GATTCAGTCTTAGCCC_NS_03
379	GCAGTTAGTCTGGAGA_NS_03
380	GCAGTTATCACAGGCC_NS_17
381	GCATACAAGGAGCGTT_NS_07
382	GCATACAGTTATCACG_NS_12
383	GCATACATCTGATTCT_NS_12
384	GCATGATCAGAAGCAC_NS_17
385	GCGAGAACATCCTTGC_NS_03
386	GCGCCAACAGTCACTA_NS_03
387	GCGCCAATCATGTGGT_NS_12

388	GCTTGAAAGATGAGAG_NS_03
389	GCTTGAAGTGGGTATG_NS_12
390	GGACATTGTTCCGTCT_NS_03
391	GGATTACGTAATTGGA_NS_17
392	GGCTGGTCACATGTGT_NS_12
393	GGGCACTGTTCCATGA_NS_17
394	GGGTCTGCACCATGTA_NS_17
395	GGTATTGCACGGCGTT_NS_12
396	GGTATTGTCGCGTAGC_NS_03
397	GGTATTGTCTTGTATC_NS_03
398	GTCAAGTGTGTGTGCC_NS_03
399	GTCAAGTTCGATGAGG_NS_12
400	GTCACGGTCAGCATGT_NS_03
401	GTCATTTGTGTCGCTG_NS_07
402	GTCCTCACATCTCCCA_NS_03
403	GTCTTCGTCGGCCGAT_NS_17
404	GTGCTTCAGTGTTAGA_NS_03
405	GTGGGTCGTTATCGGT_NS_17
406	GTTACAGCAATGGTCT_NS_17
407	GTTACAGGTGCTAGCC_NS_17
408	GTTCTCGCACATCCGG_NS_12
409	TAAACCGGTGACGGTA_NS_07
410	TACCTATTCTTAACCT_NS_03
411	TACGGATCAGGAATCG_NS_12
412	TACTTGTCACCTCGTT_NS_03
413	TAGTGGTCATGTCTCC_NS_12
414	TCAGATGGTTTCGCTC_NS_03
415	TCAGCAACAATGAAAC_NS_12
416	TCAGCAATCTTGCAAG_NS_17
417	TCCCGATTCTCTGCTG_NS_12
418	TCGAGGCGTTTGTTTC_NS_03
419	TCGCGAGAGGCAGTCA_NS_12
420	TCGTAGATCTCCCTGA_NS_12
421	TCTTTCCAGAAGGCCT_NS_12
422	TGCCAAATCTGACCTC_NS_12
423	TGCGCAGCAATCACAC_NS_12
424	TGCGCAGCACGGTAAG_NS_17
425	TGCTACCAGGCGTACA_NS_03
426	TGCTGCTCACCTATCC_NS_03
427	TGCTGCTGTGTGACGA_NS_12
428	TGGCCAGGTAGCCTCG_NS_17
429	TGGGCGTGTAAAGTCA_NS_12
430	TGGGCGTTCAGGATCT_NS_12
431	TGTATTCAGCTTATCG_NS_12
432	TGTATTCTCGGTCTAA_NS_03
433	TGTCCCAGTACCGAGA_NS_12
434	TGTCCCAGTACCGGCT_NS_07
435	TTAACTCAGTGTTAGA_NS_17
436	TTCGAAGTCAAACCGT_NS_12
437	TTCGAAGTCATACGGT_NS_17

Footnote:
The cells highlighted in orange are the same
