# Supplementary material for: Non-canonical NOTCH1 signaling regulates ferroptosis vulnerability in dormant lung cancer cells with stable resistance
Source: Cell Death Dis. 2025 Dec 26;17(1):1. doi: 10.1038/s41419-025-08355-9 (PMC12780219; doi:10.1038/s41419-025-08355-9)
Supplement: Supplementary file 10 — Supplementary Table 8 [file 41419_2025_8355_MOESM10_ESM.pdf]

Huang H et. al., Non-canonical NOTCH1 Signaling Regulates Ferroptosis Vulnerability in Dormant Lung Cancer Cells with Stable Resistance

Table S8. Primer information

Related to Fig. 3N, S1B, C

Application	Locus	Forward primer (5' to 3')	Reverse primer (5' to 3')
STR profiling	D5S818	ATGCTTTAGTGCTTTTTAGC	TCCTCTTTGGTATCCTTATG
	D7S820	CTCATTGACAGAATTGCAC	GGGTATGATAGAACACTTGTC
	D13S317	TCCTTCAACTTGGGTTGAGC	CTTTAGTGGGCATCCGTGAC
	D16S539	TCCCAAGCTCTTCCTCTTCC	AGCGTTTGTGTGTGCATCTG
	TPOX	GAGGAAGGGCTGTGTTTCAG	TAGGCCCTTCTGTCCTTGTC
	CSF1PO	AGGTTGCTAACCACCCTGTG	TGGACAGCATTTCCTGTGTC
	TH01	TCTAGCAGCAGCTCATGGTG	CACAGGGAACACAGACTCC
	VWA	TACATAGGTTAGATAGAGATAGGACAGATG	TGAGATGTGAAAGCCCTAGTGG
	Amelogenin	GTAAAAGCTACCACCTCATCCTG	CAGAGCTTAAACTGGGAAGCTG
SLC52A1		ATGTGAAGGTGGCTGCAAGC	AACACGTGGTAGATGCTGGT
SLC52A2		ACCTCGCTTCTTACGGTCAT	AGTCAGTGCCCAGAAGAAGG
SLC52A3		CCTGCCTAACAGGTCTCTGC	ACGAGGCCACAATGAGGACT
GAPDH		ACCCAGAAGACTGTGGATGG	TCTAGACGGCAGGTCAGGTC
Mycoplasma test*		GGCGAATGGGTGAGTAACACG	CGGATAACGCTTGCGACCTATG

Footnote:
*, the primers for mycoplasma test are obtained from the paper:
Development of a PCR method for mycoplasma testing of Chinese hamster ovary cell cultures used in the manufacture of recombinant therapeutic proteins
