# Supplementary material for: Non-canonical NOTCH1 signaling regulates ferroptosis vulnerability in dormant lung cancer cells with stable resistance
Source: Cell Death Dis. 2025 Dec 26;17(1):1. doi: 10.1038/s41419-025-08355-9 (PMC12780219; doi:10.1038/s41419-025-08355-9)
Supplement: Supplementary file 11 — Supplementary Table 9 [file 41419_2025_8355_MOESM11_ESM.pdf]

**Huang H et. al., Non-canonical NOTCH1 Signaling Regulates Ferroptosis Vulnerability in Dormant Lung Cancer Cells with Stable Resistance**

Table S9. Antibody information

| Antibody | Fluorochrome | Clone | Cat. no. | RRID | Manufacturer | Application |
|---|---|---|---|---|---|---|
| Human NOTCH1 | R-PE | MHN1-519 | 352105 | AB_10896419 | Biolegend | FACS (1:100) |
| Human NOTCH1 | APC | MHN1-519 | 352107 | AB_10897100 | Biolegend | FACS (1:100) |
| Human NOTCH1 (full length) | | D6F11 | 4380 | AB_10691684 | Cell Signaling | WB (1:1000) |
| Human cleaved NOTCH1 (cleaved at Val1744) | | D3B8 | 4147 | AB_2153348 | Cell Signaling | WB (1:1000) |
| Human NOTCH2 | R-PE | MHN2-25 | 348303 | AB_2153620 | Biolegend | FACS (1:100) |
| Human NOTCH3 | APC | MHN3-21 | 17-5787-42 | AB_10669568 | Thermo | FACS (1:20) |
| Human NOTCH4 | R-PE | MHN4-2 | 349003 | AB_10612931 | Biolegend | FACS (1:100) |
| Human CD82 | PE-Cy7 | ASL-24 | 342110 | AB_2750123 | Biolegend | FACS (1:200) |
| Human Ki-67 | Alexa Fluor 647 | B56 | 561126 | AB_10611874 | BD Biosciences | FACS (1:40) |
| Human HES1 | | D6P2U | 11988 | AB_2728766 | Cell Signaling | WB (1:1000) |
| Human p-STAT3 | | D3A7 | 9145 | AB_2491009 | Cell Signaling | WB (1:1000) |
| Human SLC52A1 | | | ER63589 | AB_3096357 | Huabio | WB (1:500) |
| Human SLC52A2 | | | CSB-PA881008LA01HU | | CUSABIO | FACS (1:100) |
| Human BAX (activated form) | | 6A7 | RHG13201 | | Antibody system | FACS (1:200) |
| Human GPX4 | | 230168A8 | 82949-1-RR | AB_3670692 | Proteintech | FACS (1:200) |
| Human AIFM2/ FSP1 | | 1A2B2 | 68049-1-Ig | AB_2918791 | Proteintech | FACS (1:100) |
| Human ACSL4 | | 1H5D3 | 66617-1-Ig | AB_2881977 | Proteintech | FACS (1:200) |
| Human p-MLKL (Ser358) | | EPR9514 | ab187091 | AB_2619685 | Abcam | FACS (1:100) |
| Human p-AKT (Ser473) | | 2E17 | 80455-1-RR | AB_2918892 | Proteintech | FACS (1:200) |
| Human GAPDH | | 3G7 | AT0002 | AB_2941027 | Engibody | WB (1:5000) |
| Human ATP1A1 | | 6P12 | AT0813 | | Engibody | WB (1:5000) |
| Mouse mTER-119 | APC | TER-119 | 116211 | AB_313712 | Biolegend | FACS (1:100) |
| Mouse mCD45 | APC | I3/2.3 | 147707 | AB_2563539 | Biolegend | FACS (1:100) |
| Mouse mH-2Kd | APC | SF1-1.1 | 116620 | AB_10645328 | Biolegend | FACS (1:100) |
| Anti-DYKDDDDK Tag | PerCP-Cy5.5 | L5 | 637325 | AB_2750064 | Biolegend | FACS (1:100) |
| Rabbit isotype antibody | | DA1E | 3900S | AB_1550038 | Cell Signaling | FACS (1:200) |
| Mouse isotype antibody | | 1F8D3 | 66360-1-Ig | AB_2827991 | Proteintech | FACS (1:200) |
| Mouse isotype antibody | Alexa Fluor 647 | MOPC-21 | 400130 | AB_2800436 | Biolegend | FACS (1:40) |
| Rat IgG2a isotype control | PerCP-Cy5.5 | B39-4 | E-AB-F09742J | | Elabscience | FACS (1:200) |
| Goat Anti-Rabbit secondary antibody | CoraLite Plus 555 | | RGAR003 | AB_3073507 | Proteintech | FACS (1:200) |
| Goat Anti-Rabbit secondary antibody | CoraLite Plus 594 | | RGAR004 | AB_3073508 | Proteintech | FACS (1:200) |
| Goat Anti-Mouse secondary antibody | CoraLite Plus 555 | | RGAM003 | AB_3068539 | Proteintech | FACS (1:200) |
| Goat Anti-Mouse secondary antibody | CoraLite Plus 647 | | RGAM005 | AB_3073503 | Proteintech | FACS (1:200) |
| Goat anti-Mouse IgG (H+L) | Alexa Fluor Plus 594 | | A32742 | AB_2762825 | Invitrogen | FACS (1:1000) |

**Footnote:**

| | |
|---|---|
| Biolegend | San Diego, CA |
| BD Biosciences | Franklin Lakes, NJ |
| Cell Signaling | Danvers, MA |
| Huabio | Hangzhou, China |
| Engibody | Dover, DE |
| Proteintech | Rosemont, IL |
| CUSABIO | Wuhan, China |
| Antibody system | Schiltigheim, France |
| Thermo | Waltham, MA |
