# Supplementary material for: Non-canonical NOTCH1 signaling regulates ferroptosis vulnerability in dormant lung cancer cells with stable resistance
Source: Cell Death Dis. 2025 Dec 26;17(1):1. doi: 10.1038/s41419-025-08355-9 (PMC12780219; doi:10.1038/s41419-025-08355-9)

Non-canonical NOTCH1 Signaling Regulates Ferroptosis Vulnerability in Dormant Lung Cancer Cells with Stable Resistance,

Huang H et al.

Supplementary original Western blots

Figure 5B	page 2
Supplementary Figure 1H	page 3
Supplementary Figure 1I	page 4
Supplementary Figure 5B	page 5
Supplementary Figure 5F	page 6

Figure 5B

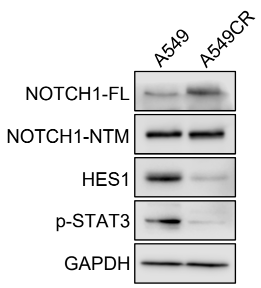

Original data

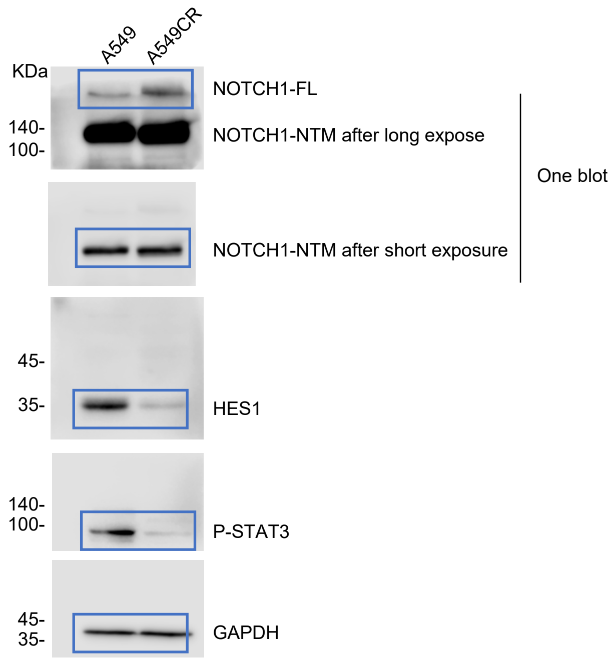

Supplementary Figure 1H

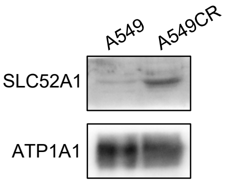

Original data

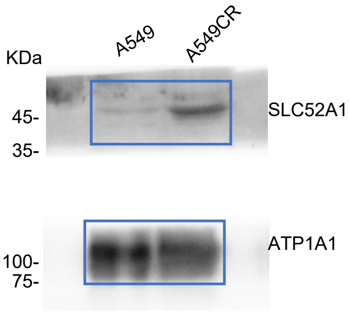

Supplementary Figure 1I

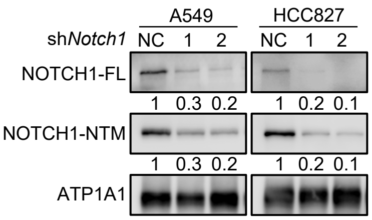

Original data

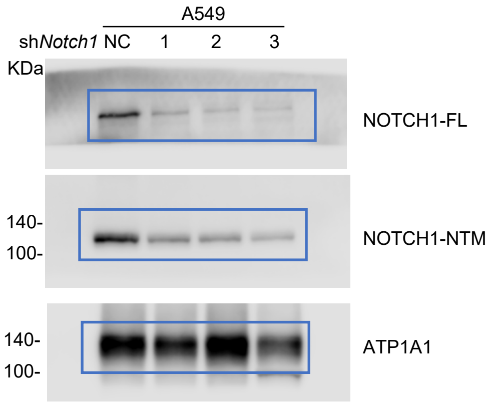

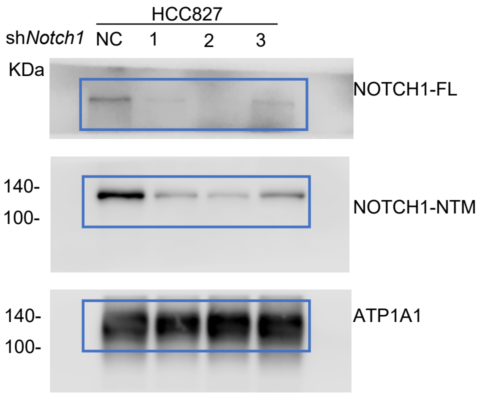

Supplementary Figure 5B

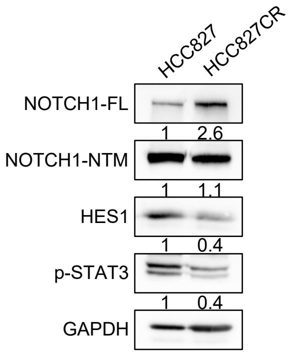

Original data

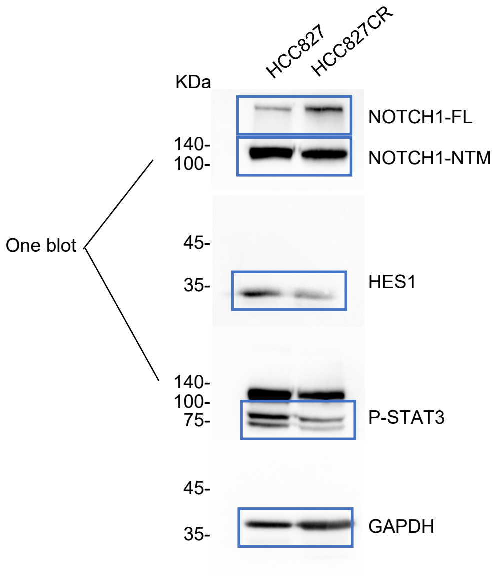

Supplementary Figure 5F

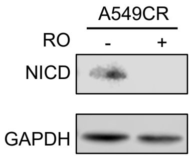

Original data

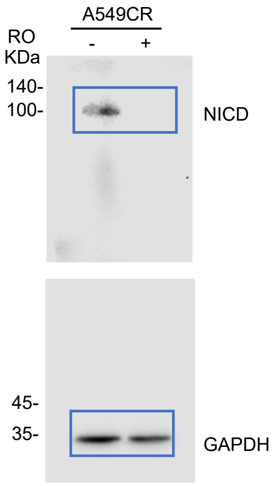

Supplement: Supplementary file 12 — Original Western blot data [file 41419_2025_8355_MOESM12_ESM.pdf]
